# Nitrosonium Tetrafluoroborate-Promoted α,α-Diacetoxylation of Aryl Methyl Ketones

**DOI:** 10.1021/acs.joc.6c00768

**Published:** 2026-06-10

**Authors:** Jyun-Jie Li, Chen-Hung Hsiao, Duen-Ren Hou

**Affiliations:** Department of Chemistry, 34911 National Central University, 300 Jhong-Da Road, Jhong-Li, Taoyuan 320317, Taiwan

## Abstract

α,α-Diacetoxylation of methyl aryl ketones was achieved using NOBF_4_ under mild, metal-free conditions. The reaction exhibits a broad substrate scope, tolerating aryl groups with diverse electronic properties, although slightly electron-deficient substrates afford higher efficiencies. Mechanistic studies support a radical pathway initiated by single-electron-transfer (SET) nitrosation of the enol form of the methyl aryl ketone.

## Graphical abstract

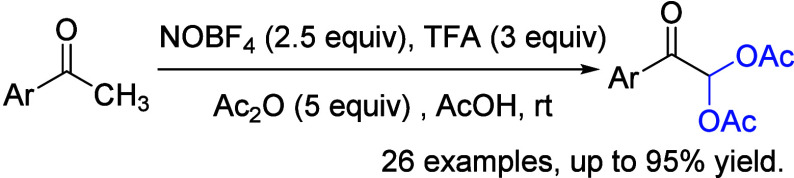

## Introduction

Geminal diacetates (*gem*-diacetates) have been widely used as alternative protecting groups for aldehydes because they are formed with high selectivity from aldehydes rather than ketones.^1^ Beyond their role as protecting groups, the excellent leaving-group ability of *gem*-diacetates enables their participation in nucleophilic substitution reactions for both C–C and C–heteroatom bond formation.^2^ They have also been utilized as acetyl donors in enzymatic alcohol acylation.^3^

Direct conversion of aldehydes into *gem*-diacetates can be achieved with acetic anhydride, and many catalysts have been developed for this purpose (Scheme 1).^4,5^ Alternative methods to access *gem*-diacetates include replacement of geminal dihalides with silver acetate^6^ and oxidation of cinnamic acids,^7^ aryl ketones,^8^ and 1,3-dicarbonyl compounds.^9^ Sulfur ylides derived from sulfonium salts of acetophenones have also been applied to prepare *gem*-diacetates.^10^ Although these reactions avoided the use of aldehydes as starting materials, their scope was constrained by the limited availability of suitable substrates. More broadly, oxidative transformations of ketones have emerged as valuable strategies for the direct functionalization of readily available carbonyl compounds.^11^ In particular, oxidative α-functionalization of aryl ketones provides efficient access to structurally diverse oxygenated molecules.^12^ A considerable amount of effort has therefore been devoted to the development of metal-free oxidants and radical-mediated processes for ketone oxidation.^13,14^ Despite these advances, direct conversion of simple acetophenones into *gem*-diacetates under mild conditions remains relatively underexplored. Recently, the use of the nitrosonium ion (NO^+^) as a versatile and mild oxidant in organic synthesis has attracted a considerable amount of attention.^15^ We have found that NO^+^ is capable of activating arenes and sulfur compounds to enable their functionalization or oxidation.^16^ Herein, we report a mild, metal-free, oxidative method for the preparation of *gem*-diacetates from readily accessible acetophenones.

**Scheme 1:**
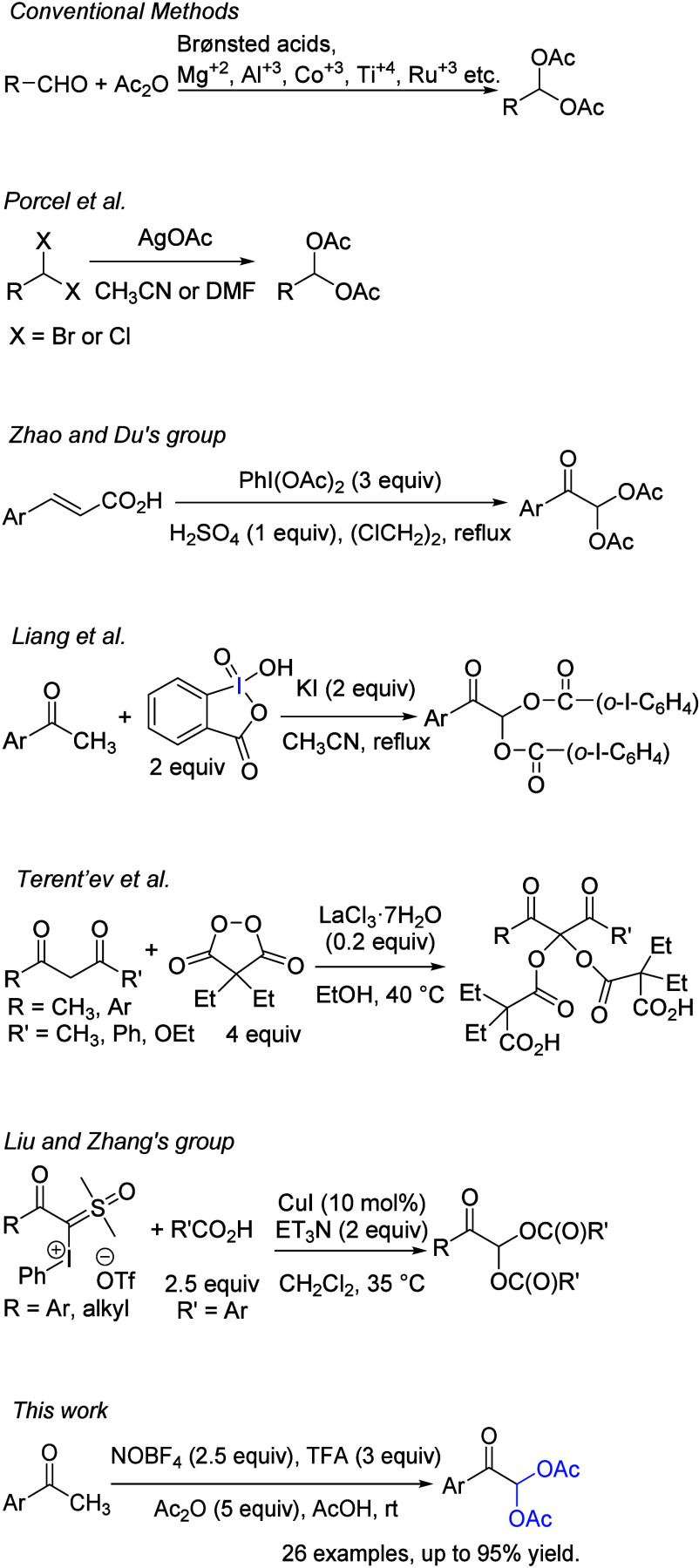
Approaches to Prepare *gem*-Diacetates

## Results and Discussion

The transformation is conducted using NOBF_4_, trifluoroacetic acid (TFA), and acetic anhydride in acetic acid (Table 1). The structure of *gem*-diacetate **2a** was assigned unambiguously by NMR spectroscopies and X-ray crystallography (Figure 1, CCDC 2520216). Notably, NOBF_4_ proved to be essential for the α,α-diacetoxylation of 1-(5-(*tert*-butyl)-2-methoxyphenyl)­ethan-1-one (**1a**) under these conditions. Two or more equivalents of NOBF_4_ was required to obtain product **2a** in high yields (entries 1–4, Table 1). Although TFA was not strictly required for the reaction to proceed, the use of 3 equiv of TFA afforded the highest yield (95%) of **2a** among the amounts examined (entries 4–7). When the reaction was conducted under an oxygen atmosphere, the yield of **2a** decreased due to the formation of nitration byproducts (entry 8). Notably, NOBF_4_ could be replaced by NO_2_BF_4_, and the reaction conducted under an oxygen atmosphere was less adversely affected than that using NOBF_4_, although a slightly longer reaction time (5 h) was required (entry 9). This difference may arise from an equilibrium between NO_2_^+^ and NO^+^ under the acidic conditions.^17^ In contrast, other nitrosonium precursors, including *tert-*butyl nitrite, sodium nitrite, sodium nitrate, nitric acid, and nitrosonium hydrogen sulfate, failed to produce **2a**; instead, starting material **1a** was recovered or underwent nitration or decomposition (entries 10–14, respectively). Substitution of TFA with hydrochloric acid or sulfuric acid resulted in poor yields of **2a** (entry 15 or 16, respectively). Additional results from the screening of acids and common organic solvents are summarized in Table S1.

**Table 1:**
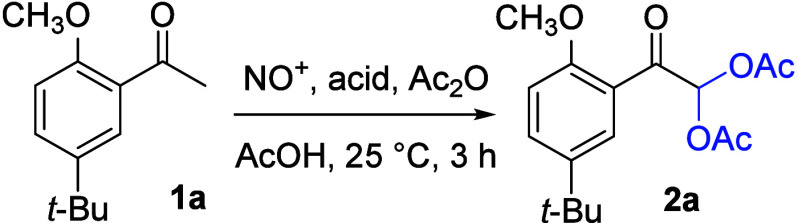
Reaction Conditions for the Formation of 1,1-Diyl Diacetate^a^

entry	NO^+^ source (equiv)	acid (equiv)	yield (%)
1	–	TFA (3.0)	0^b^
2	NOBF_4_ (1.0)	TFA (3.0)	42
3	NOBF_4_ (2.0)	TFA (3.0)	82
4	NOBF_4_ (2.5)	TFA (3.0)	91 (95)^c^
5	NOBF_4_ (3.0)	–	56
6	NOBF_4_ (3.0)	TFA (4.0)	73
7	NOBF_4_ (3.0)	TFA (2.0)	72
8	NOBF_4_ (2.5)	TFA (3.0)	43 (27)^e^
9	NO_2_BF_4_ (2.5)	TFA (3.0)	95 (88)^d^
10	*t*-C_4_H_9_-ONO (2.5)	TFA (3.0)	0^b^
11	NaNO_2_ (2.5)	TFA (3.0)	0^b^
12	NaNO_3_ (2.5)	TFA (3.0)	24^e^
13	HNO_3_ (2.5)	TFA (2.0)	–^g^
14	NOHSO_4_ (2.5)	TFA (3.0)	80^h^
15	NOBF_4_ (2.5)	HCl (3.0)^i^	28
16	NOBF_4_ (2.5)	H_2_SO_4_ (1.5)	26

a A source of nitrosonium ion was added to a solution of **1a** (61.9 mg, 0.3 mmol), TFA, acetic anhydride (153.1 mg, 1.5 mmol), and acetic acid (0.75 mL) at 25 °C. The mixture was stirred at 25 °C under a nitrogen atmosphere (balloon) for 3 h.

b Starting material **1a** was recovered.

c NOBF_4_ (0.90 mmol, 3.0 equiv) was added.

d The reaction was conducted under an oxygen atmosphere (balloon).

e 1-(5-(*tert*-Butyl)-2-methoxy-3-nitrophenyl)­ethan-1-one.

f The reaction time was 5 h.

g Decomposition.

h 4-(*tert*-Butyl)-1-methoxy-2-nitrobenzene.

i HCl_(g)_ in AcOH (1.0 M).

**Figure 1:**
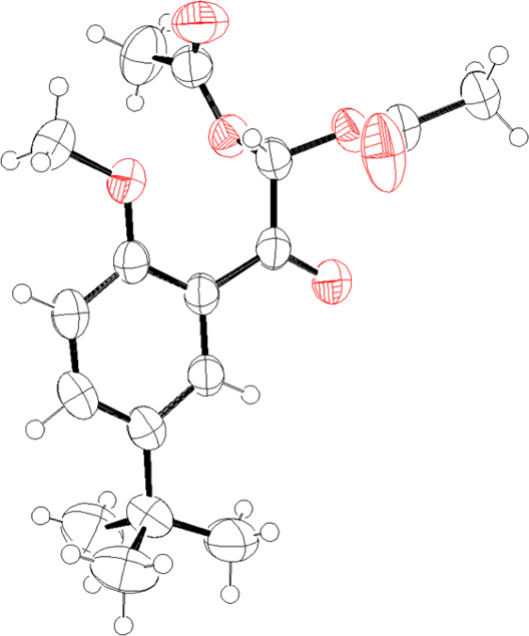
ORTEP diagram of compound **2a** with ellipsoids set to 50% probability.

With the optimized reaction conditions in hand (entry 4, Table 1), acetophenone (**1b**) and a series of its derivatives (**1c–1y**) were examined to prepare the corresponding *gem*-diacetates (Scheme 2). α,α-Diacetoxylated acetophenone **2b** was obtained in moderate yield (66%), as *gem*-diacetates **2c–2e** (42–75% yields) were derived from *p*-, *m*-, and *o*-methyl-substituted acetophenones, respectively. In contrast, *gem*-diacetate **2f** was produced in low yield, likely due to steric hindrance from the 2,4,6-trimethylphenyl group. A clear trend of increasing yields, from **2g** (37%) to **2h** (43%) and **2c** (75%), suggests an inverse correlation between the reaction efficiency and stability of the putative benzylic tertiary, secondary, and primary radicals; *i.e.*, substrates that more readily form benzylic radicals afford lower yields. In line with this interpretation, *gem*-diacetate **2i**, which cannot form a benzylic radical, was obtained in the highest yield (89%) among the examined substrates. Biphenyl and 2-naphthyl ketones afforded *gem*-diacetates **2j** and **2k**, respectively, in comparable, moderate yields. The low yields observed for electron-rich aryl ketones were often accompanied by nitrated byproducts, as shown in Table 1 (entries 8 and 12). Notably, all three fluoro-substituted acetophenones (*para*, *meta*, and *ortho*) gave higher yields of diacetates **2p–2r** (92%, 70%, and 78%, respectively) than those obtained from methoxy-substituted substrates (**2l–2n**, 46%, 39%, and 66%, respectively). A preference for electron-deficient aryl groups in this reaction was further evidenced by the yields of **2l** and **2o** (46% and 71%, respectively), consistent with their Hammett σ_para_ values (−0.12 for OMe and 0.31 for OAc).^18^ All halogenated acetophenones examined were compatible with the reaction, furnishing halogenated *gem*-diacetates **2p–2u**. Chloro-substituted diacetate **2s** was also prepared on a gram scale in 72% yield. Both methyl ester and carboxylic acid functionalities were tolerated, affording diacetates **2v** and **2w**, respectively. Higher yields observed for substrates bearing electron-withdrawing groups (EWGs), such as halogens, acetoxy, and carboxylate substituents, are consistent with the enhanced enol formation in EWG-substituted acetophenones reported by Guthrie.^19^ In contrast, *p*-nitroacetophenone was inert under the condition to form **2x**, likely because the strongly electron-withdrawing nitro group diminishes the reactivity of the resulting enol. Replacement of acetic acid and acetic anhydride with propanoic acid and its anhydride led to *gem*-dipropanoate **2y**. Finally, 2-acetylthiophene underwent α,α-diacetoxylation to afford *gem*-diacetate **2z**, albeit in low yield. Under these reaction conditions, propiophenone predominantly underwent overoxidation and C–C bond cleavage to give benzoic acid, while no reaction was observed for aliphatic ketones.

**Scheme 2:**
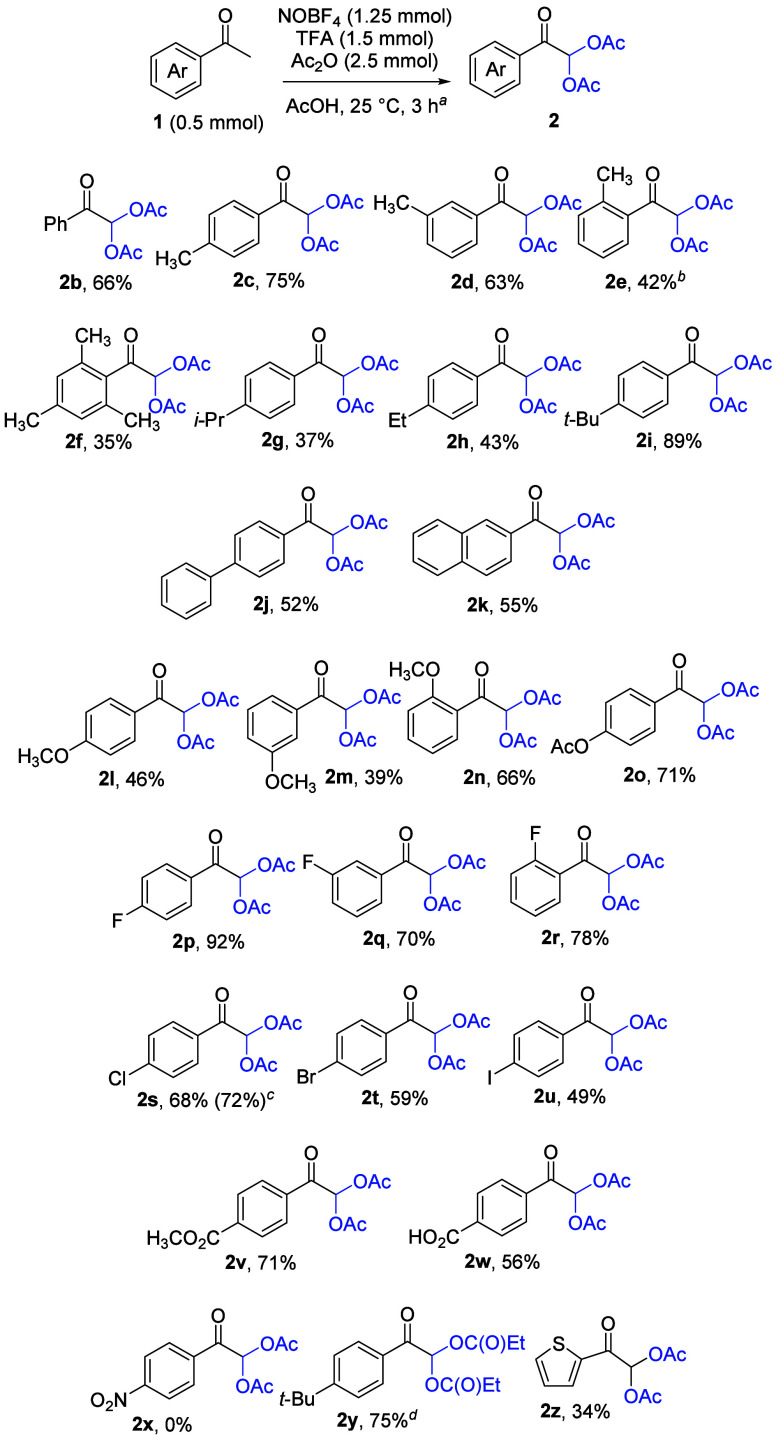
Substrate Scope of α,α-Diacetoxylation^a^

a NOBF4 (1.50 mmol) was added to a solution of aryl methyl ketone (0.50 mmol), TFA (1.5 mmol), acetic anhydride (2.5 mmol), and acetic acid (0.75 mL) at 25 °C. The mixture was stirred at 25 °C under a nitrogen atmosphere (balloon) for 3 h.
b NOBF_4_ (1.0 mmol) and TFA (1.0 mmol) were applied.
c On a gram scale.
d Propanoic anhydride (2.5 mmol) and propanoic acid (0.75 mL) were applied.

Hydrolysis of *gem*-diacetate **2b** afforded phenylglyoxal hydrate **3** (eq 1), which is in equilibrium with the corresponding aldehyde and undergoes rapid condensation with amines to furnish imine derivatives.^20^ In the absence of hydrolysis, *gem*-diacetate **2s** underwent reaction with *tert*-butyl isocyanide at the keto group to furnish adduct **4** (eq 2).
1

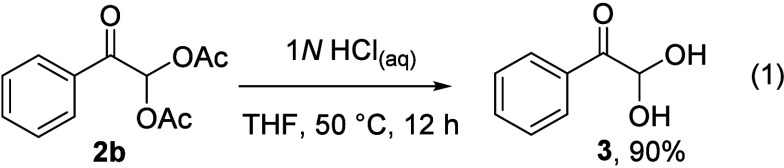



2

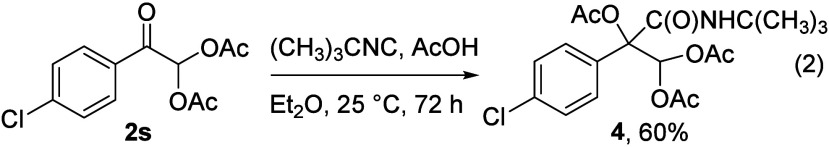




To probe the reaction mechanism, a series of control experiments were conducted. When α-hydroxyacetophenone (**1b-OH**) was subjected to the standard reaction conditions, only acetate **1b-OAc** was formed, which remained inert even with a prolonged reaction time (eq 3). In contrast, both enol acetate **5** and phenylglyoxal hydrate **3** were efficiently converted into *gem*-diacetates **2i** and **2b**, respectively, in good yields (eqs 4 and 5). These results indicate that the reaction is unlikely to proceed through monohydroxylated intermediates such as **1b-OH** or **1b-OAc**; instead, enol acetate **5**, phenylglyoxal hydrate **3**, or their equivalents are more plausible intermediates. Further evidence for a radical, single-electron-transfer (SET) pathway was obtained from inhibition experiments. The reaction was completely suppressed by the addition of 1 equiv of either 2,2,6,6-tetramethyl-1-piperidinyloxy (TEMPO) or butylated hydroxytoluene (BHT). In addition, TEMPO–enol adduct **6**, derived from the enol of **1i**, was detected by mass spectrometry (eq 6 and Figure S1). The involvement of radical intermediates also provides a rationale for the differing yields of **2g**, **2h**, **2c**, and **2i**, wherein substrates that more readily form benzylic radicals exhibit diminished product yields, as discussed above.
3

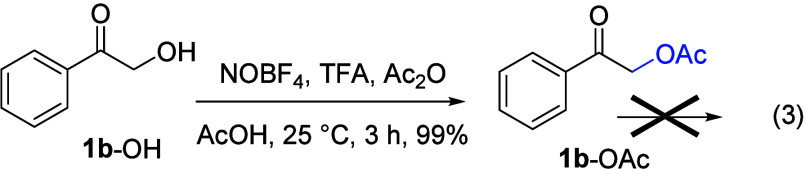



4

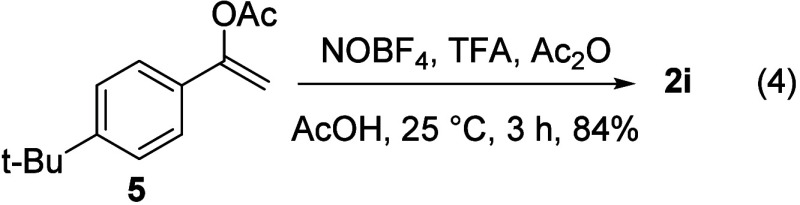



5

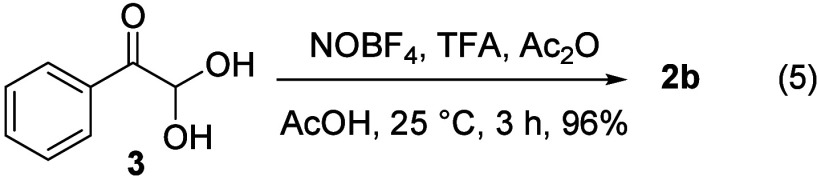



6

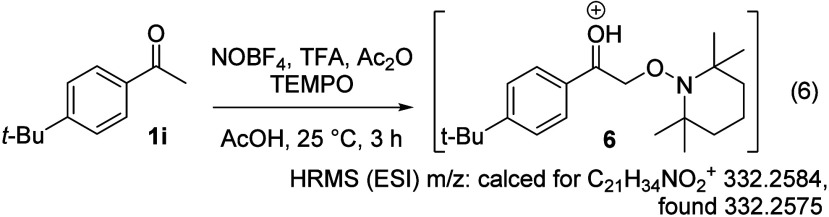




A reaction mechanism consistent with these observations is proposed in Scheme 3. The reaction of enols with nitrosonium ions to form α-nitroso ketones has been reported previously;^15d,21^ however, the possible involvement of a SET process has not been discussed. In the present system, the detection of TEMPO adduct **6**, together with the inhibitory effects of TEMPO and BHT, supports a stepwise SET process, generating cationic radical intermediate **A** and nitric oxide (NO•) from enol **1** and NO^+^. Subsequent nitrosation of **A** and proton transfer to form α-nitroso ketone **C** are followed by tautomerization and two sequential additions of acetic acid. After elimination of a hydroxylammonium ion (H_3_N^+^OH), *gem*-diacetate product **2** is formed under the acidic condition. The spontaneous reaction between H_3_N^+^OH and NO^+^ produces nitrous oxide (N_2_O) and hydronium ion (eq 7);^22^ therefore, an additional equivalent of NOBF_4_ is required to complete the reaction. The presence of N_2_O in the gaseous atmosphere of the reaction flask was confirmed by GC–MS analysis (Figure S2). The involvement of nitric oxide (NO•) rationalizes the diminished yield of **2a** when the reaction is conducted under an oxygen atmosphere (entry 8, Table 1), as oxygen readily reacts with nitric oxide.^23^

**Scheme 3:**
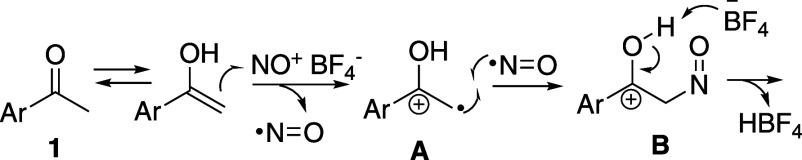
Proposed Mechanism to Form *gem*-Diacetates



7

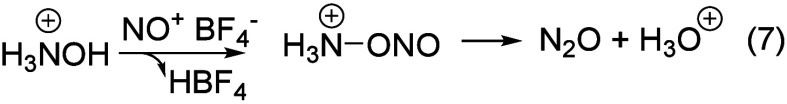




## Conclusion

In summary, we have developed a convenient, metal-free method for the preparation of *gem*-diacetates from methyl aryl ketones using NOBF_4_ as the oxidant under mild, room-temperature conditions. The transformation employs readily accessible aryl methyl ketones and displays a broad substrate scope, with higher reactivity for substrates that more readily form enols^19^ and reduced yields for those favoring competitive benzylic radical formation. Mechanistic studies indicate that the reaction proceeds via an α-nitroso ketone intermediate generated through enol nitrosation, consistent with a SET pathway.

## Experimental Section

### General Information

Reagents, such as NaNO_2_, NaNO_3_, NOBF_4_, NO_2_BF_4_, *tert*-butyl nitrite, sulfuric acid, TFA, acetic anhydride and acetic acid, were purchased from commercial sources (ACS grade) and used without further purification. Thin-layer chromatography (TLC) was conducted using precoated silica gel 60 F_254_ plates containing a fluorescent indicator; spots were examined under UV light or revealed with a KMnO_4_ solution. Purification by chromatography was conducted using silica gel (230–400 mesh). The ^1^H and ^13^C NMR spectra were recorded in a CDCl_3_ or D_2_O solution using a Bruker Avance 500 and 300 NMR spectrometer. Chemical shifts for ^1^H and ^13^C NMR spectra are reported in δ units (parts per million) with reference to residual solvent peaks. High-resolution mass spectrometry (HRMS) data were recorded on a JMS-700 quadrupole mass spectrometer. GC-MS was performed on a Thermo Scientific Trace 1300 GC+ISQ MS+ autosampler Triplus RSH with Xcalibur2.2 software and NIST2017+Wiley 10^th^ edition Library. The data for X-ray crystallography were recorded on a Bruker D8 QUEST CCD diffractometer with Mo Kα radiation (λ = 0.71073 Å), and the structures were determined with APEX3. Compounds **1a**,^24^ **1f**,^25^ **1v**,^26^ **1b-OH**,^27^ **3**,^28^ and **5** ^29^ were prepared according to the procedure described in the literature.

#### 2-(5-(*tert*-Butyl)-2-methoxyphenyl)-2-oxoethane-1,1-diyl Diacetate (**2a**)

Nitrosonium tetrafluoroborate (NOBF_4_, 175.2 mg, 1.5 mmol) was added to a solution of 1-(5-(*tert*-butyl)-2-methoxyphenyl)­ethan-1-one (**1a**, 103.0 mg, 0.50 mmol), trifluoroacetic acid (TFA, 171.1 mg, 1.50 mmol), acetic anhydride (255.3 mg, 2.50 mmol), and acetic acid (0.75 mL) at 25 °C. The reaction mixture was stirred at 25 °C under a nitrogen atmosphere (balloon) for 3 h, supplemented with NaOH_(aq)_ (3.0 M, 5.0 mL), and extracted with ethyl acetate (3 × 5 mL). The combined organic layers were dried over sodium sulfate filtered and concentrated. Product **2a** (153.1 mg, 0.48 mmol, 95%) was isolated as a colorless solid after column chromatography (SiO_2_, 1:4 EtOAc/hexanes; *R*_*f*_ = 0.32): mp 106.5–108.5 °C; ^1^H NMR (300 MHz, CDCl_3_) δ 7.90 (d, *J* = 2.6 Hz, 1H), 7.59 (s, 1H), 7.57 (dd, 1H, *J* = 8.7 Hz, *J* = 2.6 Hz), 6.89 (d, *J* = 8.7 Hz, 1H), 3.83 (s, 3H), 2.15 (s, 6H), 1.30 (s, 9H); ^13^C­{^1^H} NMR (75 MHz, CDCl_3_) δ 190.2, 169.1, 157.3, 144.0, 132.8, 128.5, 123.0, 111.3, 89.3, 55.8, 34.3, 31.4, 20.7; HRMS (ESI) *m*/*z* calcd for C_17_H_23_O_6_ (M + H)^+^ 323.1489, found 323.1475.

Single crystals of **2a** suitable for X-ray analysis were grown by slow evaporation of a solution of **2a** (127.0 mg) in ethyl acetate (∼2 mL) at room temperature.

1-(5-(*tert*-Butyl)-2-methoxy-3-nitrophenyl)­ethan-1-one (30.2 mg, 0.12 mmol, 24%) was isolated after column chromatography (SiO_2_, 1:4 EtOAc/hexanes; *R*_*f*_ = 0.4) as a yellow liquid when NOBF_4_ was replaced with sodium nitrate (entry 12, Table 1): ^1^H NMR (300 MHz, CDCl_3_) δ 7.91 (d, *J* = 2.5 Hz, 1H), 7.80 (d, *J* = 2.5 Hz, 1H), 3.9 (s, 3H), 2.65 (s, 3H), 1.33 (s, 9H); ^13^C­{^1^H} NMR (75 MHz, CDCl_3_) δ 199.2, 148.0, 135.3, 131.1, 126.2, 125.4, 122.6, 63.9, 34.9, 31.0, 30.7; HRMS (ESI) *m*/*z* calcd for C_13_H_18_NO_4_ (M + H)^+^ 252.1230, found 252.1230.

4-(*tert*-Butyl)-1-methoxy-2-nitrobenzene (83.8 mg, 0.40 mmol, 80%) was isolated after column chromatography (SiO_2_, 1:4 EtOAc/hexanes; *R*_*f*_ = 0.43) as a yellow liquid when NOBF_4_ was replaced with NOHSO_4_ (entry 14, Table 1): ^1^H NMR (300 MHz, CDCl_3_) δ 7.81 (d, *J* = 2.4 Hz, 1H), 7.53 (dd, *J* = 2.4 Hz, *J* = 8.7 Hz, 1H), 7.01 (d, *J* = 8.7 Hz, 1H), 3.90 (s, 3H), 1.28 (s, 9H); ^13^C­{^1^H} NMR (75 MHz, CDCl_3_) δ 150.8, 143.7, 139.1, 131.4, 122.5, 113.3, 56.5, 34.3, 31.1. The spectroscopic data were consistent with the reported values.^30^

#### 2-Phenyl-2-oxoethane-1,1-diyl Diacetate (**2b**)

Nitrosonium tetrafluoroborate (NOBF_4_, 146.0 mg, 1.25 mmol) was added to a solution of acetophenone (**1b**, 60.1 mg, 0.50 mmol), trifluoroacetic acid (TFA, 171.1 mg, 1.50 mmol), acetic anhydride (255.3 mg, 2.50 mmol), and acetic acid (0.75 mL) at 25 °C. The reaction mixture was stirred at 25 °C under a nitrogen atmosphere (balloon) for 3 h, supplemented with NaOH_(aq)_ (3.0 M, 5.0 mL), and extracted with ethyl acetate (3 × 5 mL). The combined organic layers were dried over sodium sulfate, filtered, and concentrated. Product **2b** (77.3 mg, 0.33 mmol, 66%) was isolated as a colorless liquid after column chromatography (SiO_2_, 1:4 EtOAc/hexanes; *R*_*f*_ = 0.47): ^1^H NMR (300 MHz, CDCl_3_) δ 7.94–7.91 (m, 2H), 7.62–7.60 (m, 2H), 7.52–7.46 (m, 2H), 2.18 (s, 6H); ^13^C­{^1^H} NMR (75 MHz, CDCl_3_) δ 188.9, 168.9, 134.4, 133.3, 129.0, 128.6, 86.4, 20.7. The spectroscopic data were consistent with the reported values.^7^

#### 2-Oxo-2-*p*-tolylethane-1,1-diyl Diacetate (**2c**)

The procedure to prepare **2b** was followed. Starting with 4′-methylacetophenone (**1c**, 67.1 mg, 0.50 mmol), compound **2c** (94.6 mg, 0.38 mmol, 75%) was isolated as a light-yellow liquid after column chromatography (SiO_2_, 1:4 EtOAc/hexanes; *R*_*f*_ = 0.39): ^1^H NMR (500 MHz, CDCl_3_) δ 7.83 (d, *J* = 8.1 Hz, 2H), 7.60 (s, 1H), 7.28 (d, *J* = 8.1 Hz, 2H), 2.42 (s, 3H), 2.17 (s, 6H); ^13^C­{^1^H} NMR (126 MHz, CDCl_3_) δ 188.5, 168.8, 145.5, 130.3, 129.7, 129.1, 86.4, 21.9, 20.7. The spectroscopic data were consistent with the reported values.^7^

#### 2-Oxo-2-*m*-tolylethane-1,1-diyl Diacetate (**2d**)

The procedure to prepare **2b** was followed. Starting with 3′-methylacetophenone (**1d**, 67.1 mg, 0.50 mmol), compound **2d** (78.7 mg, 0.32 mmol, 63%) was isolated as a light-yellow liquid after column chromatography (SiO_2_, 1:4 EtOAc/hexanes; *R*_*f*_ = 0.42): ^1^H NMR (300 MHz, CDCl_3_) δ 7.74–7.68 (m, 2H), 7.60 (s, 1H), 7.44–7.33 (m, 2H), 2.39 (s, 3H), 2.16 (s, 6H); ^13^C­{^1^H} NMR (75 MHz, CDCl_3_) δ 189.0, 168.8, 138.9, 135.2, 133.3, 129.4, 128.8, 126.1, 86.3, 21.4, 20.7. The spectroscopic data were consistent with the reported values.^7^

#### 2-Oxo-2-*o*-tolylethane-1,1-diyl Diacetate (**2e**)

The procedure to prepare **2b** was followed. Starting with 2′-methylacetophenone (**1e**, 67.1 mg, 0.50 mmol), compound **2e** (52.4 mg, 0.21 mmol, 42%) was isolated as a light-yellow liquid after column chromatography (SiO_2_, 1:4 EtOAc/hexanes; *R*_*f*_ = 0.45): ^1^H NMR (300 MHz, CDCl_3_) δ 7.63 (d, *J* = 7.7 Hz, 1H), 7.50 (s, 1H), 7.45 (t, *J* = 7.1 Hz, 1H), 7.32–7.29 (m, 2H), 2.54 (s, 3H), 2.16 (s, 6H); ^13^C­{^1^H} NMR (126 MHz, CDCl_3_) δ 192.0, 168.9, 140.3, 133.4, 132.5, 132.3, 128.8, 125.8, 87.2, 21.1, 20.6; HRMS (ESI) *m*/*z* calcd for C_13_H_14_O_5_Na (M + Na)^+^ 273.0733, found 273.0732.

#### 2-(2,4,6-Trimethylphenyl)-2-oxoethane-1,1-diyl Diacetate (**2f**)

The procedure to prepare **2b** was followed. Starting with 1-mesitylethan-1-one (**1f**, 81.0 mg, 0.50 mmol), compound **2f** (49.2 mg, 0.18 mmol, 35%) was isolated as a light-yellow liquid after column chromatography (SiO_2_, 1:4 EtOAc/hexanes; *R*_*f*_ = 0.45): ^1^H NMR (500 MHz, CDCl_3_) δ 7.28 (s, 1H), 6.83 (s, 2H), 2.47 (s, 3H), 2.27 (s, 6H), 2.10 (s, 6H); ^13^C­{^1^H} NMR (126 MHz, CDCl_3_) δ 199.6, 168.5, 139.8, 134.6, 130.9, 128.6, 88.4, 32.3, 21.2, 20.6; HRMS (ESI) *m*/*z* calcd for C_15_H_18_O_5_Na (M + Na)^+^ 301.1046, found 301.1040.

#### 2-(4-Isopropylphenyl)-2-oxoethane-1,1-diyl Diacetate (**2g**)

The procedure to prepare **2b** was followed. Starting with 4′-isopropylacetophenone (**1g**, 81.0 mg, 0.50 mmol), compound **2g** (51.0 mg, 0.18 mmol, 37%) was isolated as a light-yellow liquid after column chromatography (SiO_2_, 1:4 EtOAc/hexanes; *R*_*f*_ = 0.45): ^1^H NMR (300 MHz, CDCl_3_) δ 7.87 (d, *J* = 8.4 Hz, 2H), 7.62 (s, 1H), 7.34 (d, *J* = 8.3 Hz, 2H), 2.97 (septet, *J* = 6.9 Hz, 1H), 2.18 (s, 6H), 1.27 (d, *J* = 6.9 Hz, 6H); ^13^C­{^1^H} NMR (75 MHz, CDCl3) δ 188.4, 168.9, 156.2, 131.1, 129.3, 127.2, 86.3, 34.5, 23.7, 20.8; HRMS (ESI) *m*/*z* calcd for C_15_H_18_O_5_Na (M + Na)^+^ 301.1046, found 301.1038.

#### 2-(4-Ethylphenyl)-2-oxoethane-1,1-diyl Diacetate (**2h**)

The procedure to prepare **2b** was followed. Starting with 4′-ethylacetophenone (**1h**, 74.1 mg, 0.50 mmol), compound **2h** (57.4 mg, 0.22 mmol, 43%) was isolated as a light-yellow liquid after column chromatography (SiO_2_, 1:4 EtOAc/hexanes; *R*_*f*_ = 0.49): ^1^H NMR (300 MHz, CDCl_3_) δ 7.85 (d, *J* = 8.3 Hz, 2H), 7.61 (s, 1H), 7.31 (d, *J* = 8.3 Hz, 2H), 2.71 (q, *J* = 7.6 Hz, 2H), 2.17 (s, 6H), 1.25 (t, *J* = 7.6 Hz, 3H); ^13^C­{^1^H} NMR (75 MHz, CDCl_3_) δ 188.4, 168.8, 151.7, 130.9, 129.2, 128.5, 86.3, 29.2, 20.7, 15.1; HRMS (ESI) *m*/*z* calcd for C_14_H_16_O_5_Na (M + Na)^+^ 287.0889, found 287.0880.

#### 2-(4-*tert*-Butylphenyl)-2-oxoethane-1,1-diyl Diacetate (**2i**)

The procedure to prepare **2b** was followed. Starting with 4′-*tert*-butylacetophenone (**1i**, 88.1 mg, 0.50 mmol), compound **2i** (130.6 mg, 0.447 mmol, 89%) was isolated as a light-yellow liquid after column chromatography (SiO_2_, 1:4 EtOAc/hexanes; *R*_*f*_ = 0.42): ^1^H NMR (300 MHz, CDCl_3_) δ 7.88 (d, *J* = 8.6 Hz, 2H), 7.62 (s, 1H), 7.5 (d, *J* = 8.6 Hz, 2H), 2.17 (s, 6H), 1.34 (s, 9H); ^13^C­{^1^H} NMR (75 MHz, CDCl_3_) δ 188.4, 168.8, 158.4, 130.6, 129.0, 126.0, 86.3, 35.4, 31.1, 20.7. The spectroscopic data were consistent with the reported values.^6c^

#### 2-(Biphenyl-4-yl)-2-oxoethane-1,1-diyl Diacetate (**2j**)

The procedure to prepare **2b** was followed. Starting with 4-acetylbiphenyl (**1j**, 98.1 mg, 0.50 mmol), compound **2j** (80.9 mg, 0.259 mmol, 52%) was isolated as a yellow liquid after column chromatography (SiO_2_, 1:4 EtOAc/hexanes; *R*_*f*_ = 0.45): ^1^H NMR (300 MHz, CDCl_3_) δ 8.02 (d, *J* = 8.4 Hz, 2H), 7.73–7.62 (m, 5H), 7.50–7.41 (m, 3H), 2.20 (s, 6H); ^13^C­{^1^H} NMR (75 MHz, CDCl_3_) δ 188.5, 168.9, 147.1, 139.6, 131.9, 129.6, 129.1, 128.7, 127.6, 127.4, 86.4, 20.7. The spectroscopic data were consistent with the reported values.^6a^

#### 2-(Naphthalen-2-yl)-2-oxoethane-1,1-diyl Diacetate (**2k**)

The procedure to prepare **2b** was followed. Starting with 2-acetonaphthone (**1k**, 85.1 mg, 0.50 mmol), compound **2k** (78.7 mg, 0.28 mmol, 55%) was isolated as a light-yellow solid after column chromatography (SiO_2_, 1:4 EtOAc/hexanes; *R*_*f*_ = 0.39): mp 94.0–96.0 °C; ^1^H NMR (300 MHz, CDCl_3_) δ 8.47 (s, 1H), 8.00–7.86 (m, 4H), 7.79 (s, 1H), 7.66–7.45 (m, 2H), 2.20 (s, 6H); ^13^C­{^1^H} NMR (75 MHz, CDCl_3_) δ 188.9, 168.9, 136.1, 132.5, 131.2, 130.7, 130.0, 129.3, 129.0, 128.0, 127.2, 124.0, 86.4, 20.7. The spectroscopic data were consistent with the reported values.^7^

#### 2-(4-Methoxyphenyl)-2-oxoethane-1,1-diyl Diacetate (**2l**)

The procedure to prepare **2b** was followed. Starting with 4′-methoxyacetophenone (**1l**, 75.1 mg, 0.50 mmol), compound **2l** (61.6 mg, 0.232 mmol, 46%) was isolated as a light-yellow liquid after column chromatography (SiO_2_, 1:4 EtOAc/hexanes; *R*_*f*_ = 0.32): ^1^H NMR (300 MHz, CDCl_3_) δ 7.92 (d, *J* = 8.9 Hz, 2H), 7.60 (s, 1H), 6.95 (d, *J* = 8.9 Hz, 2H), 3.87 (s, 3H), 2.17 (s, 6H); ^13^C­{^1^H} NMR (75 MHz, CDCl_3_) δ 187.1, 168.8, 164.5, 131.3, 126.1, 114.2, 86.1, 55.6, 20.6. The spectroscopic data were consistent with the reported values.^7^

#### 2-(3-Methoxyphenyl)-2-oxoethane-1,1-diyl Diacetate (**2m**)

The procedure to prepare **2b** was followed. Starting with **1m** (75.1 mg, 0.50 mmol), compound **2m** (52.1 mg, 0.20 mmol, 39%) was isolated as a light-yellow liquid after column chromatography (SiO_2_, 1:4 EtOAc/hexanes; *R*_*f*_ = 0.39): ^1^H NMR (300 MHz, CDCl_3_) δ 7.60 (s, 1H), 7.49–7.47 (m, 2H), 7.39 (t, *J* = 7.9 Hz, 1H), 7.18–7.14 (m, 1H), 3.85 (s, 3H), 2.18 (s, 6H); ^13^C­{^1^H} NMR (75 MHz, CDCl_3_) δ 188.8, 168.8, 160.1, 134.6, 130.0, 121.5, 121.2, 113.0, 86.4, 55.6, 20.7; HRMS (ESI) *m*/*z* calcd for C_13_H_14_O_6_Na (M + Na)^+^ 289.0682, found 289.0678.

#### 2-(2-Methoxyphenyl)-2-oxoethane-1,1-diyl Diacetate (**2n**)

The procedure to prepare **2b** was followed. Starting with 2′-methoxyacetophenone (**1n**, 75.1 mg, 0.50 mmol), compound **2n** (88.2 mg, 0.33 mmol, 66%) was isolated as a light-yellow liquid after column chromatography (SiO_2_, 1:4 EtOAc/hexanes; *R*_*f*_ = 0.29): ^1^H NMR (300 MHz, CDCl_3_) δ 7.86 (dd, *J* = 7.8 Hz, *J* = 1.8 Hz, 1H), 7.58 (s, 1H),7.57–7.51 (m, 1H), 7.08–7.02 (m, 1H), 6.95 (d, *J* = 8.4 Hz, 1H), 3.85 (s, 3H), 2.14 (s, 6H); ^13^C­{^1^H} NMR (75 MHz, CDCl_3_) δ 190.3, 169.0, 159.1, 135.4, 131.8, 124.0, 121.3, 111.5, 89.2, 55.8, 20.7. The spectroscopic data were consistent with the reported values.^7^

#### 2-(4-Acetoxyphenyl)-2-oxoethane-1,1-diyl Diacetate (**2o**)

The procedure to prepare **2b** was followed. Starting with 4′-hydroxyacetophenone (**1o**, 68.1 mg, 0.50 mmol), compound **2o** (105.2 mg, 0.357 mmol, 71%) was isolated as a light-yellow liquid after column chromatography (SiO_2_, 1:4 EtOAc/hexanes; *R*_*f*_ = 0.29): ^1^H NMR (300 MHz, CDCl_3_) δ 7.95 (d, *J* = 8.7 Hz, 2H), 7.56 (s, 1H), 7.2 (d, *J* = 8.7 Hz, 2H), 2.30 (s, 3H), 2.15 (s, 6H); ^13^C­{^1^H} NMR (75 MHz, CDCl_3_) δ 187.8, 168.8, 155.3, 137.2, 130.7 (2C), 122.3 (2C), 86.3, 21.2, 20.7; HRMS (ESI) *m*/*z* calcd for C_14_H_14_O_7_Na (M + Na)^+^ 317.0631, found 317.0621.

#### 2-(4-Fluorophenyl)-2-oxoethane-1,1-diyl Diacetate (**2p**)

The procedure to prepare **2b** was followed. Starting with 4′-fluoroacetophenone (**1p**, 69.1 mg, 0.50 mmol), compound **2p** (116.6 mg, 0.46 mmol, 92%) was isolated as a light-yellow liquid after column chromatography (SiO_2_, 1:4 EtOAc/hexanes; *R*_*f*_ = 0.45): ^1^H NMR (300 MHz, CDCl_3_) δ 7.97 (dd, *J* = 8.6 Hz, *J* = 5.4 Hz, 2H), 7.56 (d, *J* = 0.4 Hz, 1H), 7.16 (t, *J* = 11.2 Hz, 2H), 2.17 (d, *J* = 0.5 Hz, 6H); ^13^C­{^1^H} NMR (75 MHz, CDCl_3_) δ 187.5, 168.8, 166.5 (d, *J*_C–F_ = 255.6 Hz), 131.8 (d, *J*_C–F_ = 9.5 Hz), 129.7, 116.3 (d, *J*_C–F_ = 22.0 Hz), 86.4, 20.7. The spectroscopic data were consistent with the reported values.^7^

#### 2-(3-Fluorophenyl)-2-oxoethane-1,1-diyl Diacetate (**2q**)

The procedure to prepare **2b** was followed. Starting with 3′-fluoroacetophenone (**1q**, 69.1 mg, 0.50 mmol), compound **2q** (88.4 mg, 0.348 mmol, 70%) was isolated as a light-yellow liquid after column chromatography (SiO_2_, 1:4 EtOAc/hexanes; *R*_*f*_ = 0.39): ^1^H NMR (300 MHz, CDCl_3_) δ 7.71–7.68 (m, 1H), 7.65–7.61 (m, 1H), 7.53 (s, 1H), 7.51–7.44 (m, 1H), 7.35–7.29 (m, 1H), 2.17 (s, 6H); ^13^C­{^1^H} NMR (75 MHz, CDCl_3_) δ 188.0, 168.8, 162.9 (d, *J*_C–F_ = 247.2 Hz), 135.2 (d, *J*_C–F_ = 6.6 Hz), 130.8 (d, *J*_C–F_ = 7.6 Hz), 124.7, 121.5 (d, *J*_C–F_ = 21.4 Hz), 115.8 (d, *J*_C–F_ = 22.7 Hz), 86.5, 20.7; HRMS (ESI) *m*/*z* calcd for C_12_H_11_FO_5_Na (M + Na)^+^ 277.0482, found 277.0471.

#### 2-(2-Fluorophenyl)-2-oxoethane-1,1-diyl Diacetate (**2r**)

The procedure to prepare **2b** was followed. Starting with 2′-fluoroacetophenone (**1r**, 69.1 mg, 0.50 mmol), compound **2r** (98.8 mg, 0.39 mmol, 78%) was isolated as a light-yellow liquid after column chromatography (SiO_2_, 1:4 EtOAc/hexanes; *R*_*f*_ = 0.39): ^1^H NMR (300 MHz, CDCl_3_) δ 7.94–7.88 (m, 1H), 7.63–7.56 (m, 1H), 7.41 (d, *J* = 2.4 Hz, 1H), 7.31–7.25 (m, 1H), 7.18–7.11 (m, 1H), 2.16 (s, 6H); ^13^C­{^1^H} NMR (75 MHz, CDCl_3_) δ 187.7, 168.8, 161.9 (d, *J*_C–F_ = 253.6 Hz), 136.1 (d, *J*_C–F_ = 9.3 Hz), 131.5, 125.1, 122.2 (d, *J*_C–F_ = 13.5 Hz), 116.7 (d, *J*_C–F_ = 23.3 Hz), 88.7 (d, *J*_C–F_ = 7.1 Hz), 20.5; HRMS (ESI) *m*/*z* calcd for C_12_H_11_FO_5_Na (M + Na)^+^ 277.0482, found 277.0480.

#### 2-(4-Chlorophenyl)-2-oxoethane-1,1-diyl Diacetate (**2s**)

The procedure to prepare **2b** was followed. Starting with 4′-chloroacetophenone (**1s**, 77.3 mg, 0.50 mmol), compound **2s** (90.9 mg, 0.34 mmol, 68%) was isolated as a light-yellow liquid after column chromatography (SiO_2_, 1:4 EtOAc/hexanes; *R*_*f*_ = 0.45): ^1^H NMR (300 MHz, CDCl_3_) δ 7.87 (d, *J* = 8.6 Hz, 2H), 7.54 (s, 1H), 7.46 (d, *J* = 8.6 Hz, 2H), 2.16 (s, 6H); ^13^C­{^1^H} NMR (75 MHz, CDCl_3_) δ 188.0, 168.8, 141.0, 131.6, 130.4, 129.4, 86.4, 20.7. The spectroscopic data were consistent with the reported values.^7^ A gram-scale reaction was conducted with **1s** (927.5 mg, 6.0 mmol), NOBF_4_ (1.75 g, 15.0 mmol), TFA (1.71 g, 15.0 mmol), and acetic anhydride (3.06 g, 30 mmol) in acetic acid (9 mL) to afford product **2s** (1.17 g, 4.3 mmol, 72%) after column chromatography.

#### 2-(4-Bromophenyl)-2-oxoethane-1,1-diyl Diacetate (**2t**)

The procedure to prepare **2b** was followed. Starting with 4′-bromoacetophenone (**1t**, 99.5 mg, 0.50 mmol), compound **2t** (93.7 mg, 0.30 mmol, 59%) was isolated as a light-yellow solid after column chromatography (SiO_2_, 1:4 EtOAc/hexanes; *R*_*f*_ = 0.45): mp 70.0–72.0 °C; ^1^H NMR (300 MHz, CDCl_3_) δ 7.79 (d, *J* = 8.6 Hz, 2H), 7.64 (d, *J* = 8.6 Hz, 2H), 7.54 (s, 1H), 2.17 (s, 6H); ^13^C­{^1^H} NMR (75 MHz, CDCl_3_) δ 188.2, 168.8, 132.4, 132.0, 130.4, 129.8, 86.4, 20.7. The spectroscopic data were consistent with the reported values.^7^

#### 2-(4-Iodophenyl)-2-oxoethane-1,1-diyl Diacetate (**2u**)

The procedure to prepare **2b** was followed. Starting with 4′-iodoacetophenone (**1u**, 123.0 mg, 0.50 mmol), compound **2u** (88.3 mg, 0.244 mmol, 49%) was isolated as a light-yellow solid after column chromatography (SiO_2_, 1:4 EtOAc/hexanes; *R*_*f*_ = 0.45): mp 121.0–123.5 °C; ^1^H NMR (500 MHz, CDCl_3_) δ 7.86–7.85 (m, 2H), 7.64–7.62 (m, 2H), 7.52 (d, *J* = 0.9 Hz, 1H), 2.16 (s, 6H); ^13^C­{^1^H} NMR (126 MHz, CDCl_3_) δ 188.5, 168.7, 138.4, 132.6, 130.2, 102.8, 86.4, 20.7; HRMS (ESI) *m*/*z* calcd for C_12_H_11_IO_5_Na (M + Na)^+^ 384.9543, found 384.9531.

#### 2-(4′-Methoxycarbonylphenyl)-2-oxoethane-1,1-diyl Diacetate (**2v**)

The procedure to prepare **2b** was followed. Starting with methyl 4-acetylbenzoate (**1v**, 89.1 mg, 0.50 mmol), compound **2v** (104.3 mg, 0.355 mmol, 71%) was isolated as a light-yellow solid after column chromatography (SiO_2_, 1:4 EtOAc/hexanes; *R*_*f*_ = 0.29): mp 110.0–112.0 °C; ^1^H NMR (300 MHz, CDCl_3_) δ 8.13 (d, *J* = 8.4 Hz, 2H), 7.97 (d, *J* = 8.4 Hz, 2H), 7.57 (s, 1H), 3.94 (s, 3H), 2.16 (s, 6H); ^13^C­{^1^H} NMR (75 MHz, CDCl_3_) δ 188.8, 168.8, 166.0, 136.6, 134.9, 130.1, 128.9, 86.6, 52.7, 20.7; HRMS (ESI) *m*/*z* calcd for C_14_H_14_NaO_7_ (M + Na)^+^ 317.0631, found 317.0623.

#### 2-(4′-Carboxylphenyl)-2-oxoethane-1,1-diyl Diacetate (**2w**)

The procedure to prepare **2b** was followed. Starting with methyl 4-acetylbenzoic acid (**1w**, 82.1 mg, 0.50 mmol), compound **2w** (78.4 mg, 0.28 mmol, 56%) was isolated as a light-yellow solid after column chromatography (SiO_2_, 1:4 EtOAc/hexanes; *R*_*f*_ = 0.29): mp 270.0–274.0 °C; ^1^H NMR (300 MHz, CDCl_3_) δ 8.22 (d, *J* = 8.2 Hz, 2H), 8.02 (d, *J* = 8.2 Hz, 2H), 7.59 (s, 1H), 2.19 (s, 6H); ^13^C­{^1^H} NMR (126 MHz, CDCl_3_) δ 188.8, 170.4, 168.8, 137.4, 134.0, 130.7, 129.0, 86.7, 20.7; HRMS (ESI) *m*/*z* calcd for C_13_H_12_O_7_Na (M + Na)^+^ 303.0475, found 303.0476.

#### 2-(4-(*tert*-Butyl)­phenyl)-2-oxoethane-1,1-diyl Dipropionate (**2y**)

Nitrosonium tetrafluoroborate (NOBF_4_, 116.9 mg, 1.0 mmol) was added to a solution of 4-*tert*-butylacetophenone (**1i**, 88.1 mg, 0.50 mmol), trifluoroacetic acid (TFA, 114.0 mg, 1.0 mmol), propanic anhydride (325.4 mg, 2.50 mmol), and propanic acid (0.75 mL) at 25 °C. The reaction mixture was stirred at 25 °C under a nitrogen atmosphere (balloon) for 3 h, supplemented with NaOH_(aq)_ (3.0 M, 5.0 mL), and extracted with ethyl acetate (3 × 5 mL). The combined organic layers were dried over sodium sulfate, filtered, and concentrated. Product **2y** (120.3 mg, 0.38 mmol, 75%) was isolated as a light-yellow liquid after column chromatography (SiO_2_, 1:4 EtOAc/hexanes; *R*_*f*_ = 0.49): ^1^H NMR (300 MHz, CDCl_3_) δ 7.90 (d, *J* = 7.9 Hz, 2H), 7.67 (s, 1H), 7.52 (d, *J* = 7.9 Hz, 2H), 2.48 (q, *J* = 7.2 Hz, 4H), 1.36 (s, 9H), 1.19 (t, *J* = 7.2 Hz, 6H); ^13^C­{^1^H} NMR (75 MHz, CDCl_3_) δ 188.5, 172.3, 158.2, 130.6, 128.9, 125.9, 86.2, 35.3, 31.0, 27.2, 8.7; HRMS (ESI) *m*/*z* calcd for C_18_H_24_NaO_5_ (M + Na)^+^ 343.1515, found 343.1504.

#### 2-(Thiophen-2-yl)-2-oxoethane-1,1-diyl Diacetate (**2z**)

The procedure to prepare **2b** was followed. Starting with 2-acetylthiophene (**1z**, 63.1 mg, 0.5 mmol), compound **2z** (41.4 mg, 0.17 mmol, 34%) was isolated as a light-yellow liquid after column chromatography (SiO_2_, 1:4 EtOAc/hexanes; *R*_*f*_ = 0.38): ^1^H NMR (300 MHz, CDCl_3_) δ 7.83–7.82 (m, 1H), 7.77–7.75 (m, 1H), 7.43 (s, 1H), 7.18–7.15 (m, 1H), 2.18 (s, 6H); ^13^C­{^1^H} NMR (75 MHz, CDCl_3_) δ 182.0, 168.7, 139.3, 135.9, 134.2, 128.6, 86.7, 20.7; HRMS (ESI) *m*/*z* calced for C_10_H_10_O_5_SNa (M + Na)^+^ 265.0141, found 265.0144.

#### 3-(*tert*-Butylamino)-2-(4-chlorophenyl)-3-oxopropane-1,1,2-triyl Triacetate (**4**)

*tert*-Butyl isocyanide (43.6 mg, 0.525 mmol) was added to a solution of **2s** (135.3 mg, 0.50 mmol), acetic acid (31.5 mg, 0.52 mmol), and diethyl ether (1 mL) at 25 °C. The reaction mixture was stirred at 25 °C for 72 h and concentrated. The crude product was purified by column chromatography (SiO_2_, EtOAc; *R*_*f*_ = 0.3) to give **4** (124.0 mg, 0.30 mmol, 60%) isolated as a colorless liquid: ^1^H NMR (300 MHz, CDCl_3_) δ 7.58 (s, 1H), 7.44–7.34 (m, 4H), 5.65 (s, 1H), 2.17 (s, 3H), 2.02 (s, 3H), 2.01 (s, 3H), 1.30 (s, 9H); ^13^C­{^1^H} NMR (75 MHz, CDCl_3_) δ 168.4, 168.1, 167.7, 165.9, 135.0, 132.6, 131.2, 128.8, 128.3, 86.9, 83.2, 51.9, 28.4, 21.3, 20.7, 20.6; HRMS (ESI) *m*/*z* calcd for C_19_H_24_ClNO_7_Na (M + Na)^+^ 436.1133, found 436.1117.

#### Hydrolysis of **2b** to Afford Phenylglyoxal Hydrate **3**

A reaction mixture of **2b** (22.3 mg, 0.10 mmol), hydrochloric acid (1 N, 1 mL), and THF (2 mL) was stirred at 50 °C (oil bath) for 12 h, neutralized with NaHCO_3(aq)_ to pH 7, and extracted with ether (3 × 6 mL). The combined organic layers were concentrated to give **3** (12.2 mg, 0.09 mmol, 90%) as a viscous liquid: ^1^H NMR (300 MHz, CDCl3) δ 8.13–8.11 (m, 2H), 7.66–7.55 (m, 1H), 7.52–7.49 (m, 2H), 5.98 (s, 1H), 4.27 (br, 2H); ^13^C­{^1^H} NMR (75 MHz, CDCl_3_) δ 194.6, 189.7, 134.8, 130.3, 130.0, 128.9, 87.3. The spectroscopic data were consistent with the reported values.^31^

#### Reaction Using α-Hydroxyacetophenone (**1b-OH**)

Nitrosonium tetrafluoroborate (NOBF_4_, 146.0 mg, 1.25 mmol) was added to a solution of **1b-OH** (68.1 mg, 0.50 mmol), trifluoroacetic acid (TFA, 171.1 mg, 1.50 mmol), acetic anhydride (255.3 mg, 2.50 mmol), and acetic acid (0.75 mL) at 25 °C. The reaction mixture was stirred at 25 °C under a nitrogen atmosphere (balloon) for 3 h, supplemented with NaOH_(aq)_ (3.0 M, 5.0 mL), and extracted with ethyl acetate (3 × 5 mL). The combined organic layers were dried over sodium sulfate, filtered, and concentrated. Product **1b-OAc** (89.0 mg, 0.50 mmol, 99%) was isolated as a colorless liquid after column chromatography (SiO_2_, 1:4 EtOAc/hexanes; *R*_*f*_ = 0.36): ^1^H NMR (300 MHz, CDCl_3_) δ 7.93–7.89 (m, 2H), 7.63–7.57 (m, 1H), 7.51–7.45 (m, 2H), 5.34 (s, 2H), 2.22 (s, 3H).^32^ No further reaction was observed when compound **1b-OAc** was resubjected to the condition described above.

#### Reaction Using (4-*tert*-Butylphenyl)­vinyl Acetate (**5**)

Nitrosonium tetrafluoroborate (NOBF_4_, 146.0 mg, 1.25 mmol) was added to a solution of **5** (109.2 mg, 0.50 mmol), trifluoroacetic acid (TFA, 171.1 mg, 1.50 mmol), acetic anhydride (255.3 mg, 2.50 mmol), and acetic acid (0.75 mL) at 25 °C. The reaction mixture was stirred at 25 °C under a nitrogen atmosphere (balloon) for 3 h, supplemented with NaOH_(aq)_ (3.0 M, 5.0 mL), and extracted with ethyl acetate (3 × 5 mL). The combined organic layers were dried over sodium sulfate, filtered, and concentrated. Product **2i** (122.8 mg, 0.42 mmol, 84%) was isolated as a colorless liquid after column chromatography (SiO_2_, 1:4 EtOAc/hexanes; *R*_*f*_ = 0.42).

#### Reaction Using Phenylglyoxal Hydrate (**3**)

Nitrosonium tetrafluoroborate (NOBF_4_, 146.0 mg, 1.25 mmol) was added to a solution of **3** (67.1 mg, 0.50 mmol), trifluoroacetic acid (TFA, 171.1 mg, 1.50 mmol), acetic anhydride (255.3 mg, 2.50 mmol), and acetic acid (0.75 mL) at 25 °C. The reaction mixture was stirred at 25 °C under a nitrogen atmosphere (balloon) for 3 h, supplemented with NaOH_(aq)_ (3.0 M, 5.0 mL), and extracted with ethyl acetate (3 × 5 mL). The combined organic layers were dried over sodium sulfate, filtered, and concentrated. Product **2b** (113.9 mg, 0.482 mmol, 96%) was isolated as a colorless liquid after column chromatography (SiO_2_, 1:4 EtOAc/hexanes; *R*_*f*_ = 0.47).

## Supplementary Material





## Data Availability

The data underlying this study are available in the published article and its .

## Notes

FAIR data, including the primary NMR FID files, for compounds **2a–2w**, **2y**, **2z**, **3**, and **4** (ZIP)
  Screening of acids and solvents, mass and GC-mass analytical data, and ^1^H and ^13^C NMR spectra (PDF)

## References

[1] Kochhar K. S., Bal B. S., Deshpande R. P., Rajadhyaksha S. N., Pinnick H. W. (1983). Protecting groups in organic synthesis.
Part 8. Conversion of aldehydes into geminal diacetates. J. Org. Chem..

[2] Sandberg M., Sydnes L. K. (2000). The Chemistry of
Acylals. 3. Cyanohydrin
Esters from Acylals with Cyanide Reagents. Org.
Lett..

[3] Koszelewski D., Brodzka A., Madej A., Trzepizur D., Ostaszewski R. (2021). Evaluation of gem-Diacetates as Alternative
Reagents
for Enzymatic Regio- and Stereoselective Acylation of Alcohols. J. Org. Chem..

[4] Vekariya R. H., Patel H. D. (2015). Sulfonated polyethylene glycol (PEG-OSO3H)
as a polymer supported biodegradable and recyclable catalyst in green
organic synthesis: recent advances. RSC Adv..

[5] Khan A. T., Choudhury L. H., Ghosh S. (2006). Silica supported perchloric
acid (HClO4-SiO2): A highly efficient and reusable catalyst for geminal
diacylation of aldehydes under solvent-free conditions. J. Mol. Catal. A: Chem..

[6] Nolla-Saltiel R., Carrillo-Arcos U. A., Porcel S. (2014). Silver Acetate Mediated Acetoxylations of Alkyl Halides. Synthesis.

[7] Liu L., Zhang-Negrerie D., Du Y., Zhao K. (2015). Hypervalent Iodine
Mediated C–C Double Bond Activation: A Cascade Access to α-Keto
Diacetates from Readily Available Cinnamic Acids. Synthesis.

[8] Pan Z.-l., Liu X.-y., Liang Y.-m. (2004). A new useful entry of IBX: the synthesis
and structure of α-(2-iodobenzoyloxy)­ketones. Tetrahedron Lett..

[9] Terent’ev A. O., Vil V. A., Gorlov E. S., Nikishin G. I., Pivnitsky K. K., Adam W. (2016). Lanthanide-Catalyzed Oxyfunctionalization
of 1,3-Diketones, Acetoacetic
Esters, And Malonates by Oxidative C–O Coupling with Malonyl
Peroxides. J. Org. Chem..

[10] Wei J., Zou W.-X., Hu Q., Bao M.-Z., Shen D.-T., Xiao L., Song J.-L., Liu X., Zhang S.-S. (2024). Metal-free
cascade O–H double insertion between I­(III)/S­(VI)-ylides, carboxylic
acids, and alcohols: modular access to unsymmetrical α,α-O,O-substituted
ketones. Org. Chem. Front..

[11] a Uyanik, M. ; Ishihara, K. Catalytic Oxidation in Organic Synthesis; Georg Thieme Verlag KG: Stuttgart, Germany, 2018; Vol. 10.

[12] Parrales G. M., Hollin N. C., Song F., Lyu Y., Martin A.-M. O., Strom A. E. (2024). Mechanism of Iron-Catalyzed Oxidative
α-Amination of Ketones with Sulfonamides. J. Org. Chem..

[13] Basdevant B., Legault C. Y. (2015). Enantioselective
Iodine­(III)-Mediated Synthesis of α-Tosyloxy Ketones: Breaking
the Selectivity Barrier. Org. Lett..

[14] Caminos D. A., Puiatti M., Bardagí J. I., Peñéñory A. B. (2017). Anions involved in the initiation
of the thermally induced SRN1 reaction for α-arylation of ketones. RSC Adv..

[15] De Angelis L., Pei C., Narro A. L., Wherritt D., Koenigs R. M., Doyle M. P. (2023). Polyfunctionalization
of vicinal carbon centers and synthesis of unsymmetric 1,2,3,4-tetracarbonyl
compounds. Nat. Commun..

[16] Chen P.-H., Hsu S.-J., Hou D.-R. (2024). Nitrosonium
Ion Catalyzed Oxidative Bromination of Arenes. Adv. Synth. Catal..

[17] Ziouane Y., Leturcq G. (2018). New Modeling of Nitric
Acid Dissociation Function of Acidity and Temperature. ACS Omega.

[18] Ritchie C. D., Sager W. F. (1964). An Examination of
Structure-Reactivity Relationships. Prog. Phys.
Org. Chem..

[19] Guthrie J. P. (1979). The enol
content of simple carbonyl compounds: a kinetic approach. Can. J. Chem..

[20] Takahashi K. (1968). The Reaction
of Phenylglyoxal with Arginine Residues in Proteins. J. Biol. Chem..

[21] a Williams, D. L. H. Chapter 4 - Aliphatic and alicyclic C-nitrosation. In Nitrosation Reactions and the Chemistry of Nitric Oxide; Williams, D. L. H. , Ed.; Elsevier Science: Amsterdam, 2004; pp 79–92.

[22] Bennett M. R., Maya L., Brown G. M., Posey F. A. (1982). Oxidation of hydroxylamine
by nitrous and nitric acids. Inorg. Chem..

[23] Galliker B., Kissner R., Nauser T., Koppenol W. H. (2009). Intermediates in
the Autoxidation of Nitrogen Monoxide. Chem.
- Eur. J..

[24] Tazoe K., Uchikawa Y., Feng X., Yamato T. (2012). Synthesis and Structure
of 2,3-Bis­(5-tert-butyl-2-methoxyphenyl)­buta-1,3-diene by Bromine
Elimination of (Z)-1,4-Dibromo-2,3-bis­(5-tert-butyl-2-methoxyphenyl)-2-butene. Synth. Commun..

[25] Jana A., Das K., Kundu A., Thorve P. R., Adhikari D., Maji B. (2020). A Phosphine-Free
Manganese Catalyst Enables Stereoselective Synthesis of (1 + n)-Membered
Cycloalkanes from Methyl Ketones and 1,n-Diols. ACS Catal..

[26] Hou Z., Nakanishi I., Kinoshita T., Takei Y., Yasue M., Misu R., Suzuki Y., Nakamura S., Kure T., Ohno H., Murata K., Kitaura K., Hirasawa A., Tsujimoto G., Oishi S., Fujii N. (2012). Structure-Based Design
of Novel Potent Protein Kinase CK2 (CK2) Inhibitors with Phenyl-azole
Scaffolds. J. Med. Chem..

[27] Liu X., Chen H. B., Pan Z. G., Xu J. H., Li H. X. (2011). Microwave-assisted
synthesis of α-hydroxy aromatic ketones from α-bromo aromatic
ketones in water. Chin. Chem. Lett..

[28] Liu Y., Zhang Y., Xiao W., Wang H., Yang H., Yang X., Fu H. (2025). Sodium Trifluoromethanesulfinate-Promoted
Photocatalytic C–H Acylation of N-Heterocycles with 4-Acyl-1,4-dihydropyridines
in Air. Org. Lett..

[29] Feng Z., Zhu B., Dong B., Cheng L., Li Y., Wang Z., Wu J. (2021). Visible-Light-Promoted
Synthesis of α-CF2H-Substituted Ketones
by Radical Difluoromethylation of Enol Acetates. Org. Lett..

[30] Zeegers P. J., Thompson M. J. (1992). 13C NMR spectra of substituted o-nitroanisoles and
n-butyl o-nitrophenyl ethers. Magn. Reson. Chem..

[31] Samanta S., Mondal S., Hajra A. (2018). A convergent
synthesis of vinyloxyimidazopyridine
via Cu­(i)-catalyzed three-component coupling. Org. Biomol. Chem..

[32] Zhou X., Ma H., Cao J., Liu X., Huang G. (2016). Novel and efficient
transformation of enamides into α-acyloxy ketones via an acyl
intramolecular migration process. Org. Biomol.
Chem..

