# Correction to “Nitrosonium Tetrafluoroborate-Promoted α,α-Diacetoxylation of Aryl Methyl Ketones”

**DOI:** 10.1021/acs.joc.6c01624

**Published:** 2026-08-10

**Authors:** Jyun-Jie Li, Chen-Hung Hsiao, Duen-Ren Hou

During the production process, Scheme 3 (Proposed Mechanism) was inadvertently truncated, resulting in the omission of the final two lines of the scheme. The complete Scheme 3 is provided below:

**Scheme 3:**
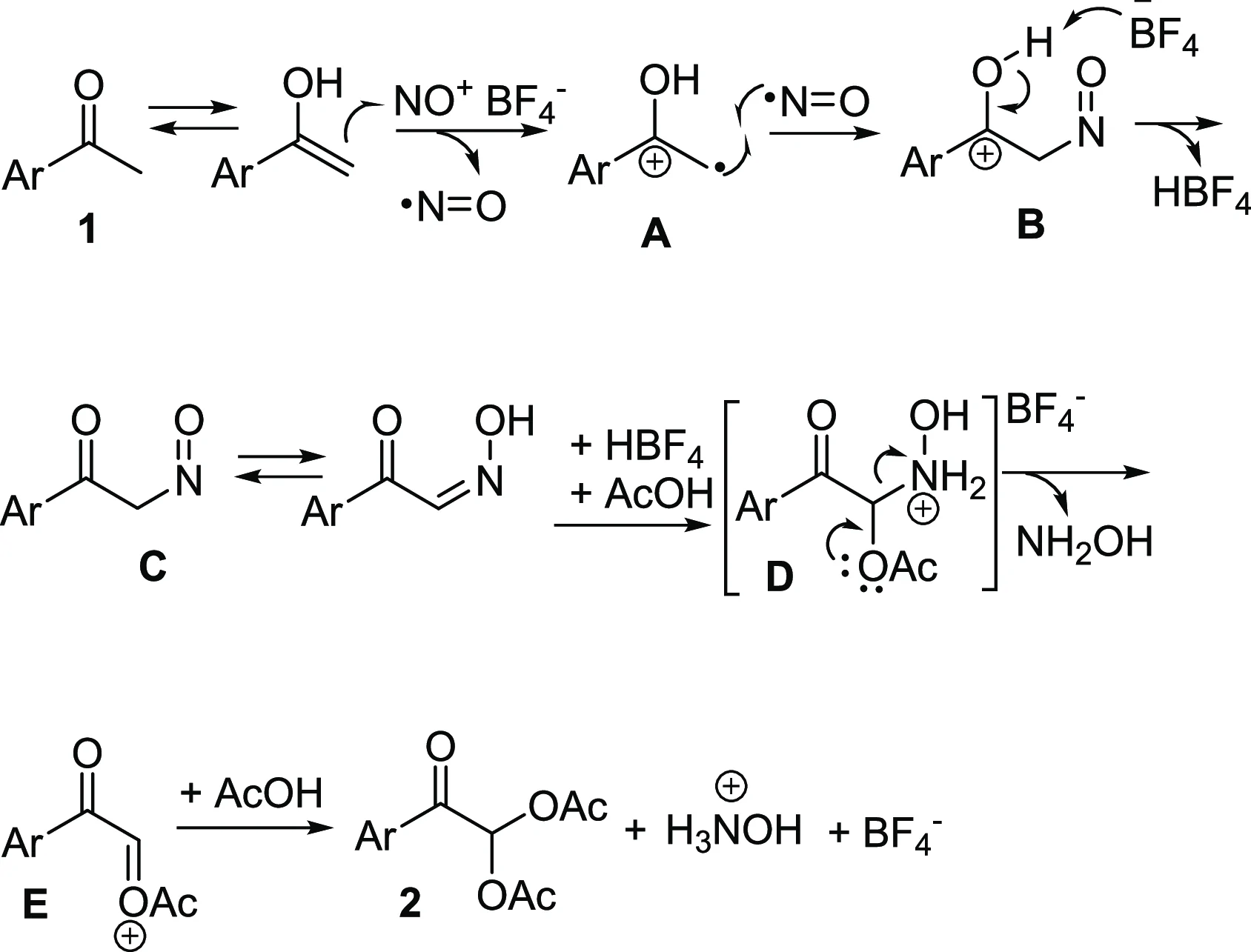
Proposed Mechanism to Form *gem*-Diacetates

