# Supplementary figures and images for: Nitrosonium Tetrafluoroborate-Promoted α,α-Diacetoxylation of Aryl Methyl Ketones
Source: J Org Chem. 2026 Jun 10;91(24):8347–55. doi: 10.1021/acs.joc.6c00768 (PMC13288677; doi:10.1021/acs.joc.6c00768)

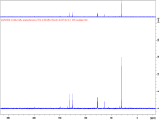

Supplement: Supplementary file 1 [file jo6c00768_si_001.zip › FID/1-(5-(tert-butyl)-2-methoxy-3-nitrophenyl)ethan-1-one/13C/pdata/1/thumb.png]

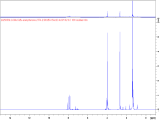

Supplement: Supplementary file 1 [file jo6c00768_si_001.zip › FID/1-(5-(tert-butyl)-2-methoxy-3-nitrophenyl)ethan-1-one/1H/pdata/1/thumb.png]

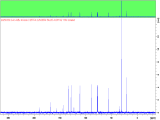

Supplement: Supplementary file 1 [file jo6c00768_si_001.zip › FID/2a/13C/pdata/1/thumb.png]

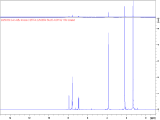

Supplement: Supplementary file 1 [file jo6c00768_si_001.zip › FID/2a/1H/pdata/1/thumb.png]

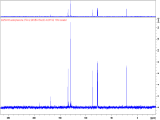

Supplement: Supplementary file 1 [file jo6c00768_si_001.zip › FID/2b/13C/pdata/1/thumb.png]

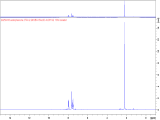

Supplement: Supplementary file 1 [file jo6c00768_si_001.zip › FID/2b/1H/pdata/1/thumb.png]

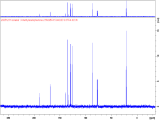

Supplement: Supplementary file 1 [file jo6c00768_si_001.zip › FID/2d/13C/pdata/1/thumb.png]
