# Supplementary material for: Nitrosonium Tetrafluoroborate-Promoted α,α-Diacetoxylation of Aryl Methyl Ketones
Source: J Org Chem. 2026 Jun 10;91(24):8347–55. doi: 10.1021/acs.joc.6c00768 (PMC13288677; doi:10.1021/acs.joc.6c00768)
Supplement: Supplementary file 2 [file jo6c00768_si_002.pdf]

Supporting Information

Nitrosonium Tetrafluoroborate Promoted α,α-Diacetoxylation of Aryl Methyl Ketones

Jyun-Jie Li, Chen-Hung Hsiao and Duen-Ren Hou*

Department of Chemistry, National Central University, No. 300 Jhong-Da Rd., Jhong-li, Taoyuan, Taiwan, 320317.

Table of Contents

Table S1. Acid and Solvent Screening for α,α-diacetoxylation……………….S5

Figure S1. Mass Analysis of TEMPO-adduct **6**…………………………………S6

Figure S2. GC-mass analysis for the reaction of **1i** to **2i**.……………………......S7

Table S2. Crystal data and structure refinement for **2a** (CCDC 2520216)…..S8

^{1}H NMR of compound **2a**..S16

^{13}C{^{1}H} NMR of compound **2a**..S17

^{1}H NMR of 1-(5-(*tert*-butyl)-2-methoxy-3-nitrophenyl)ethan-1-one…….........S18

^{13}C{^{1}H} NMR of 1-(5-(*tert*-butyl)-2-methoxy-3-nitrophenyl)ethan-1-one.......S19

^{1}H NMR of compound **2b**..S20

^{13}C{^{1}H} NMR of compound **2b**..S21

^{1}H NMR of compound **2c**..S22

^{13}C{^{1}H} NMR of compound **2c**..S23

^{1}H NMR of compound **2d**..S24

^{13}C{^{1}H} NMR of compound **2d**..S25

^{1}H NMR of compound **2e**..S26

^{13}C{^{1}H} NMR of compound **2e**..S27

^{1}H NMR of compound **2f**..S28

^{13}C{^{1}H} NMR of compound **2f**..S29

^{1}H NMR of compound **2g**..S30

^{13}C{^{1}H} NMR of compound **2g**..S31

^{1}H NMR of compound **2h**..S32

^{13}C{^{1}H} NMR of compound **2h**..S33

^{1}H NMR of compound **2i**..S34

$^{13}C\{^{1}H\}$ NMR of compound **2i**..S35

^{1}H NMR of compound **2j**..S36

$^{13}C\{^{1}H\}$ NMR of compound **2j**..S37

^{1}H NMR of compound **2k**..S38

$^{13}C\{^{1}H\}$ NMR of compound **2k**..S39

^{1}H NMR of compound **2l**..S40

$^{13}C\{^{1}H\}$ NMR of compound **2l**..S41

^{1}H NMR of compound **2m**..S42

$^{13}C\{^{1}H\}$ NMR of compound **2m**..S43

^{1}H NMR of compound **2n**..S44

$^{13}C\{^{1}H\}$ NMR of compound **2n**..S45

^{1}H NMR of compound **2o**..S46

$^{13}C\{^{1}H\}$ NMR of compound **2o**..S47

^{1}H NMR of compound **2p**..S48

$^{13}C\{^{1}H\}$ NMR of compound **2p**..S49

^{1}H NMR of compound **2q**..S50

$^{13}C\{^{1}H\}$ NMR of compound **2q**..S51

^{1}H NMR of compound **2r**..S52

$^{13}C\{^{1}H\}$ NMR of compound **2r**..S53

^{1}H NMR of compound **2s**..S54

$^{13}C\{^{1}H\}$ NMR of compound **2s**..S55

^{1}H NMR of compound **2t**..S56

$^{13}C\{^{1}H\}$ NMR of compound **2t**..S57

^{1}H NMR of compound **2u**..S58

$^{13}C\{^{1}H\}$ NMR of compound **2u**..S59

^{1}H NMR of compound **2v**..S60

$^{13}C\{^{1}H\}$ NMR of compound **2v**..S61

^{1}H NMR of compound **2w**..S62

$^{13}C\{^{1}H\}$ NMR of compound **2w**..S63

^{1}H NMR of compound **2y**..S64

$^{13}C\{^{1}H\}$ NMR of compound **2y**..S65

^{1}H NMR of compound **2z**..S66

$^{13}C\{^{1}H\}$ NMR of compound **2z**..S67

^{1}H NMR of compound **3**..S68

$^{13}C\{^{1}H\}$ NMR of compound **3**..S69

^{1}H NMR of compound **4**..S70

$^{13}C\{^{1}H\}$ NMR of compound **4**..S71

Table S1. Acid and Solvent Screening for α,α-diacetoxylation.[a]

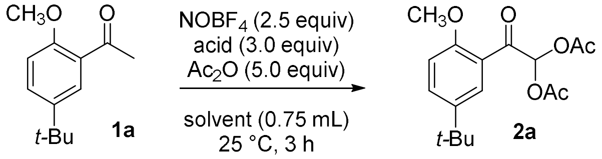

entry	solvent	acid	yield (%)[b]
1	CH_2Cl_2	TFA	30
2	$ClCH_2CH_2Cl$	TFA	27
3	CH_3NO_2	TFA	15
4	CH_3CN	TFA	7
5	DMSO	TFA	0[c]
6	ethyl acetate	TFA	80[d]
7	ethanol	TFA	0[e]
8	THF	TFA	0[e]
9	acetic acid	HNO_3	0[c]
10	acetic acid	TsOH	70[f]
11	acetic acid	Oxalic acid	40[f]

[a]NOBF4 (0.75 mmol) was added to a solution of **1a** (61.9 mg, 0.3 mmol), TFA, acetic anhydride (153.1 mg, 1.5 mmol) and solvent (0.75 mL) at 25 °C. The reaction mixture was stirred at 25 °C under an atmosphere of nitrogen (balloon) for 3 h. [b]Yields were determined by ^{1}H NMR using dibromomethane as the internal standard. [c]Decomposed. [d]1-(5-(*tert*-Butyl)-2-methoxy-3-nitrophenyl)ethan-1-one. [e]Starting material **1a** recovered. [f]4-(*tert*-Butyl)-1-methoxy-2-nitrobenzene.

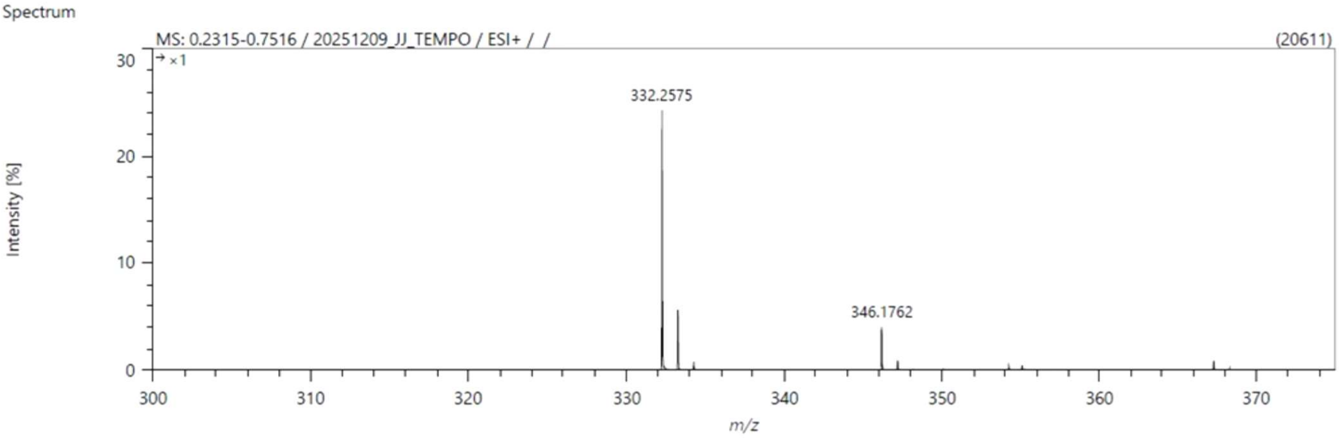

Elemental Composition

Parameters	
Tolerance:	±10.00 ppm
Electron:	Odd/Even
Charge:	+1
DBE:	-99.0 - 999.0

Elements Set 2:

Symbol	C	H	O	N
Min	0	0	2	1
Max	400	1000	2	1

Results

Mass	Formula	Calculated Mass	Mass Difference [mDa]	Mass Difference [ppm]	DBE
332.25751	C21 H34 N O2	332.25841	-0.90	-2.71	5.5

Figure S1. Mass Analysis of TEMPO-adduct **6**

Experimental procedure: nitrosonium tetrafluoroborate ($NOBF_4$, 116.1 mg, 1.0 mmol) was added to a solution of 4'-*tert*-butylacetophenone (**1i**, 88.1 mg, 0.50 mmol), trifluoroacetic acid (TFA, 114.1 mg, 1.0 mmol), acetic anhydride (255.3 mg, 2.50 mmol), and acetic acid (0.75 mL). Subsequently, TEMPO (2,2,6,6-tetramethylpiperidin-1-oxyl, 78.1 mg, 0.50 mmol) was added at 25 °C. The resulting reaction mixture was stirred at 25 °C under a nitrogen atmosphere (balloon) for 3 h and then analyzed by mass spectrometry.

Compared with air blank (red).

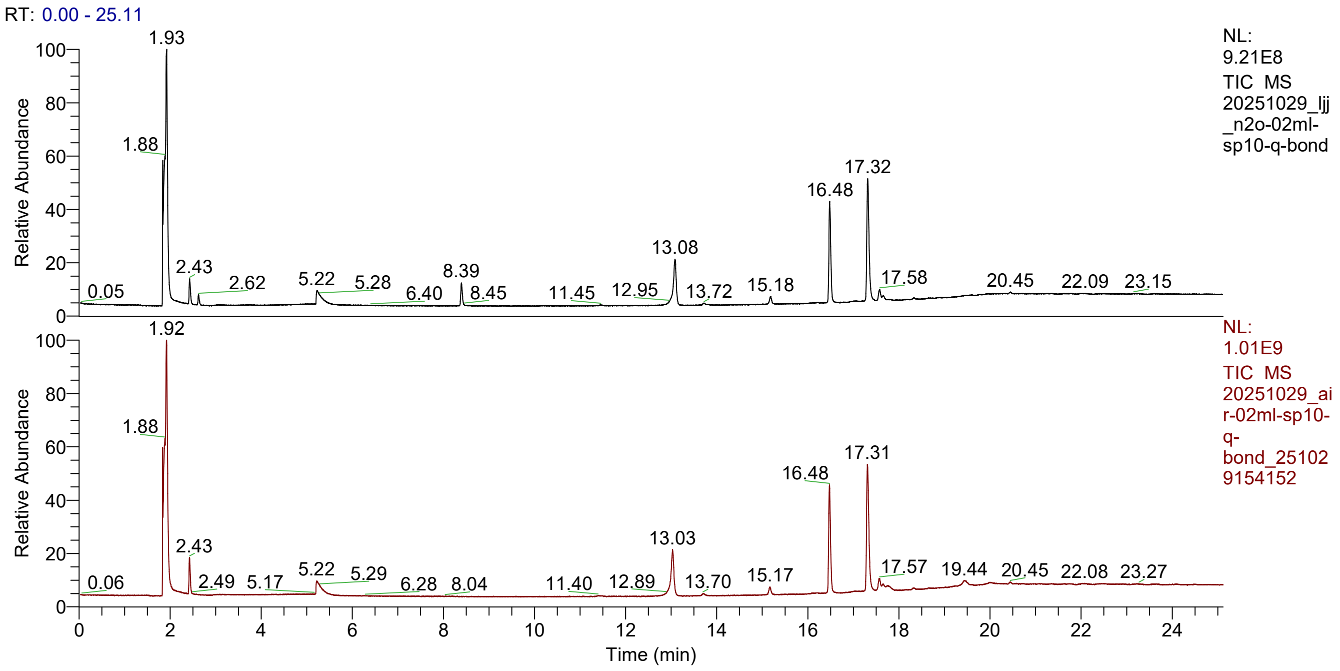

Retention time **2.62 min**:

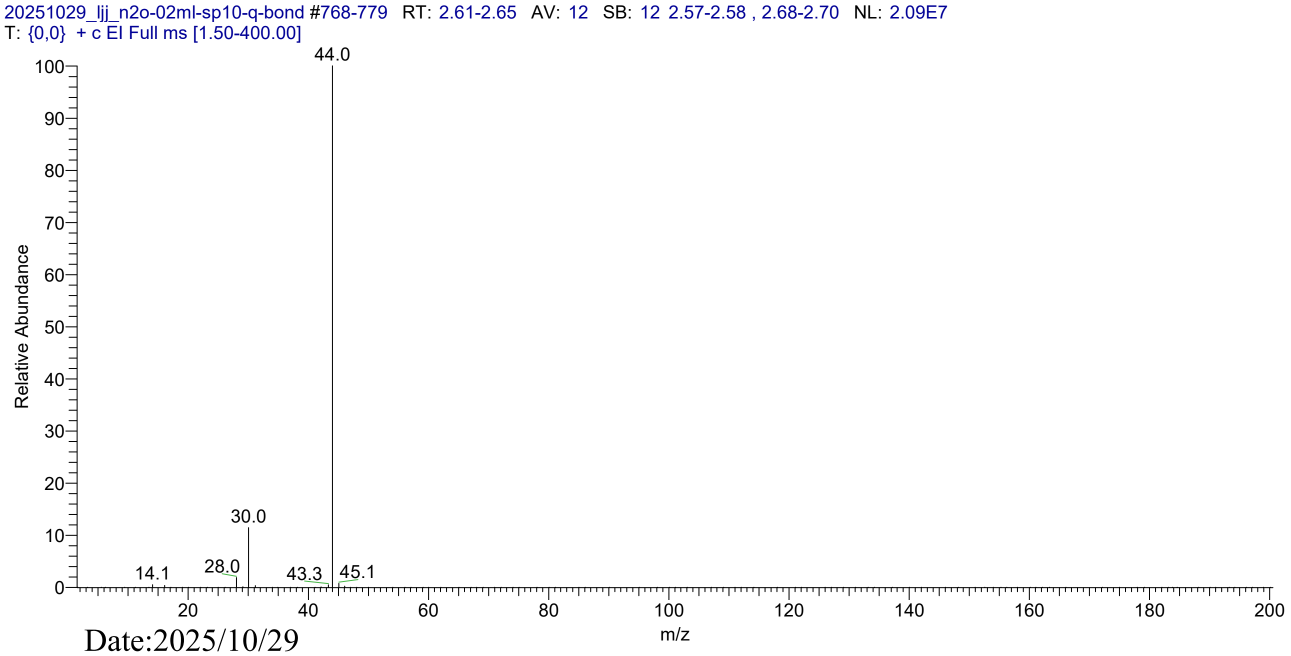

Date:2025/10/29

Sample Name: LJJ_N2O

Column: RT-Q-Bond 30m I.D.:0.32mm film:10μm Carrier gas: He

Flow rate: 1.3mL/min Injection volume: 0.2mL gas Split 10:1

Inlet temperature: 180 Temperature program:

#	Rate (°C/min)	Temperature (°C)	Hold Time (min)
Initial		40.0	0.00
1	10.0	240.0	5.00

Solvent delay: no

Mass range: 1.5-400

Figure S2. GC-mass analysis for the reaction of **1i** to **2i**

Table S2. Crystal data and structure refinement for **2a** (CCDC 2520216)

Crystal data	
Chemical formula	$C_{17}H_{22}O_6$
M_r	322.35
Crystal system, space group	Triclinic, *P*¯1
Temperature (K)	301
a, *b*, *c* (Å)	7.6566 (7), 10.4978 (7), 12.2172 (12)
〈, ®, © (°)	73.489 (3), 75.099 (3), 69.339 (2)
V ($Å^3$)	867.37 (13)
Z	2
Radiation type	Mo *K*〈
µ (mm^{-1})	0.09
Crystal size (mm)	0.21 × 0.11 × 0.06
Data collection	
Diffractometer	Bruker D8 QUEST
Absorption correction	Multi-scan<br>*SADABS2016*/2 was used for absorption correction.
T_{min}, T_{max}	0.681, 0.746
No. of measured, independent and observed [*I* > 2 ſ(*I*)] reflections	18682, 3174, 2540
R_{int}	0.040
(sin ⋁/⅃)$_{max}$ ($Å^{-1}$)	0.602
Refinement	
R[F^2 > 2 ſ(F^2)], *wR*(F^2), *S*	0.059, 0.175, 1.09

No. of reflections	3174
No. of parameters	214
H-atom treatment	H-atom parameters constrained
⊗⟩$_{max}$, ⊗⟩$_{min}$ (e Å^{-3})	0.26, -0.20

Computer programs: *APEX5* v2023.9-2 (Bruker AXS), *SAINT* V8.40B (Bruker AXS LLC, 2019), XT, VERSION 2018/2, *SHELXL2019*/1 (Sheldrick, 2019), shelXle (C.B. Huebschle, rev 1380).

Bond lengths [Å]

O1—C8	1.356 (3)
O1—C11	1.423 (3)
O2—C12	1.213 (3)
O3—C16	1.372 (3)
O3—C13	1.409 (2)
O4—C16	1.186 (3)
O5—C14	1.340 (3)
O5—C13	1.426 (2)
O6—C14	1.194 (3)
C1—C2	1.519 (4)
C1—C5	1.525 (3)
C1—C4	1.533 (4)
C1—C3	1.535 (4)
C2—H2A	0.9600
C2—H2B	0.9600
C2—H2C	0.9600
C3—H3A	0.9600

C3—H3B	0.9600
C3—H3C	0.9600
C4—H4A	0.9600
C4—H4B	0.9600
C4—H4C	0.9600
C5—C10	1.383 (3)
C5—C6	1.401 (3)
C6—C7	1.371 (3)
C6—H6	0.9300
C7—C8	1.388 (3)
C7—H7	0.9300
C8—C9	1.407 (3)
C9—C10	1.397 (3)
C9—C12	1.488 (3)
C10—H10	0.9300
C11—H11A	0.9600
C11—H11B	0.9600
C11—H11C	0.9600
C12—C13	1.530 (3)
C13—H13	0.9800
C14—C15	1.487 (3)
C15—H15A	0.9600
C15—H15B	0.9600
C15—H15C	0.9600
C16—C17	1.482 (3)
C17—H17A	0.9600

C17—H17B	0.9600
C17—H17C	0.9600

Bond angles [°]

C8—O1—C11	119.45 (18)
C16—O3—C13	115.49 (17)
C14—O5—C13	114.93 (15)
C2—C1—C5	112.1 (2)
C2—C1—C4	108.8 (3)
C5—C1—C4	108.2 (2)
C2—C1—C3	109.6 (3)
C5—C1—C3	110.5 (2)
C4—C1—C3	107.5 (2)
C1—C2—H2A	109.5
C1—C2—H2B	109.5
H2A—C2—H2B	109.5
C1—C2—H2C	109.5
H2A—C2—H2C	109.5
H2B—C2—H2C	109.5
C1—C3—H3A	109.5
C1—C3—H3B	109.5
H3A—C3—H3B	109.5
C1—C3—H3C	109.5
H3A—C3—H3C	109.5
H3B—C3—H3C	109.5
C1—C4—H4A	109.5
C1—C4—H4B	109.5

H4A—C4—H4B	109.5
C1—C4—H4C	109.5
H4A—C4—H4C	109.5
H4B—C4—H4C	109.5
C10—C5—C6	115.8 (2)
C10—C5—C1	123.6 (2)
C6—C5—C1	120.61 (19)
C7—C6—C5	122.79 (19)
C7—C6—H6	118.6
C5—C6—H6	118.6
C6—C7—C8	120.5 (2)
C6—C7—H7	119.7
C8—C7—H7	119.7
O1—C8—C7	123.02 (19)
O1—C8—C9	118.15 (17)
C7—C8—C9	118.83 (19)
C10—C9—C8	118.61 (18)
C10—C9—C12	115.95 (18)
C8—C9—C12	125.37 (19)
C5—C10—C9	123.4 (2)
C5—C10—H10	118.3
C9—C10—H10	118.3
O1—C11—H11A	109.5
O1—C11—H11B	109.5
H11A—C11—H11B	109.5
O1—C11—H11C	109.5

H11A—C11—H11C	109.5
H11B—C11—H11C	109.5
O2—C12—C9	120.8 (2)
O2—C12—C13	118.53 (18)
C9—C12—C13	120.61 (18)
O3—C13—O5	106.36 (15)
O3—C13—C12	105.82 (17)
O5—C13—C12	110.60 (16)
O3—C13—H13	111.3
O5—C13—H13	111.3
C12—C13—H13	111.3
O6—C14—O5	122.7 (2)
O6—C14—C15	125.0 (2)
O5—C14—C15	112.34 (19)
C14—C15—H15A	109.5
C14—C15—H15B	109.5
H15A—C15—H15B	109.5
C14—C15—H15C	109.5
H15A—C15—H15C	109.5
H15B—C15—H15C	109.5
O4—C16—O3	123.0 (2)
O4—C16—C17	126.8 (2)
O3—C16—C17	110.2 (2)
C16—C17—H17A	109.5
C16—C17—H17B	109.5
H17A—C17—H17B	109.5

C16—C17—H17C	109.5
H17A—C17—H17C	109.5
H17B—C17—H17C	109.5

Bond torsion angles [°]

C2—C1—C5—C10	10.1 (3)
C4—C1—C5—C10	-109.9 (3)
C3—C1—C5—C10	132.7 (3)
C2—C1—C5—C6	-171.2 (3)
C4—C1—C5—C6	68.8 (3)
C3—C1—C5—C6	-48.6 (3)
C10—C5—C6—C7	1.2 (3)
C1—C5—C6—C7	-177.6 (2)
C5—C6—C7—C8	0.0 (3)
C11—O1—C8—C7	-2.0 (3)
C11—O1—C8—C9	177.79 (19)
C6—C7—C8—O1	177.7 (2)
C6—C7—C8—C9	-2.1 (3)
O1—C8—C9—C10	-176.84 (17)
C7—C8—C9—C10	3.0 (3)
O1—C8—C9—C12	6.2 (3)
C7—C8—C9—C12	-174.0 (2)
C6—C5—C10—C9	-0.3 (3)
C1—C5—C10—C9	178.49 (19)
C8—C9—C10—C5	-1.8 (3)
C12—C9—C10—C5	175.44 (19)
C10—C9—C12—O2	-5.5 (3)

C8—C9—C12—O2	171.5 (2)
C10—C9—C12—C13	176.13 (18)
C8—C9—C12—C13	-6.8 (3)
C16—O3—C13—O5	-91.2 (2)
C16—O3—C13—C12	151.09 (17)
C14—O5—C13—O3	163.82 (17)
C14—O5—C13—C12	-81.7 (2)
O2—C12—C13—O3	107.1 (3)
C9—C12—C13—O3	-74.6 (2)
O2—C12—C13—O5	-7.7 (3)
C9—C12—C13—O5	170.66 (18)
C13—O5—C14—O6	2.4 (3)
C13—O5—C14—C15	-178.52 (18)
C13—O3—C16—O4	5.8 (3)
C13—O3—C16—C17	-173.75 (19)

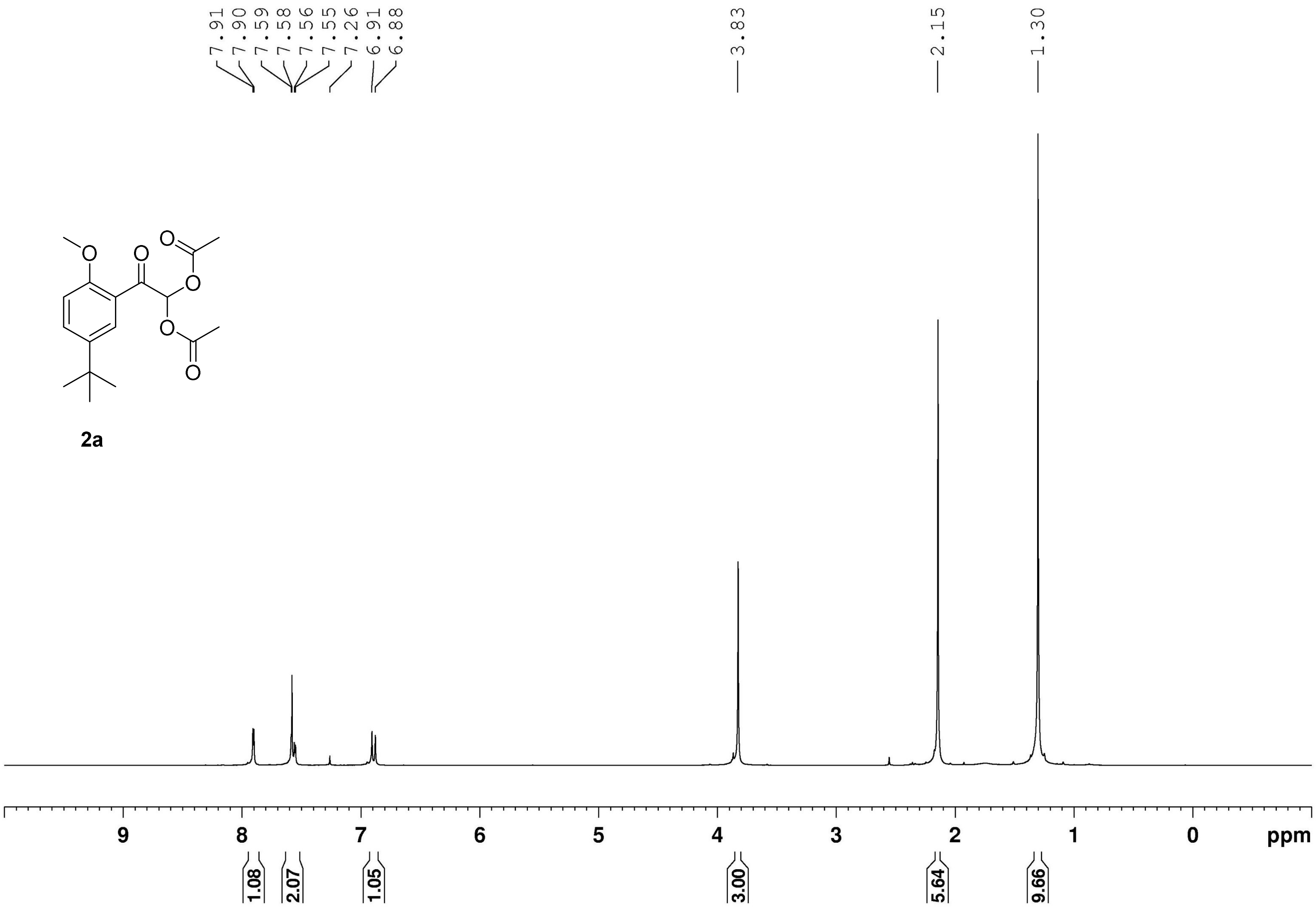

^{1}H NMR of compound **2a** (300 MHz, $CDCl_3$)

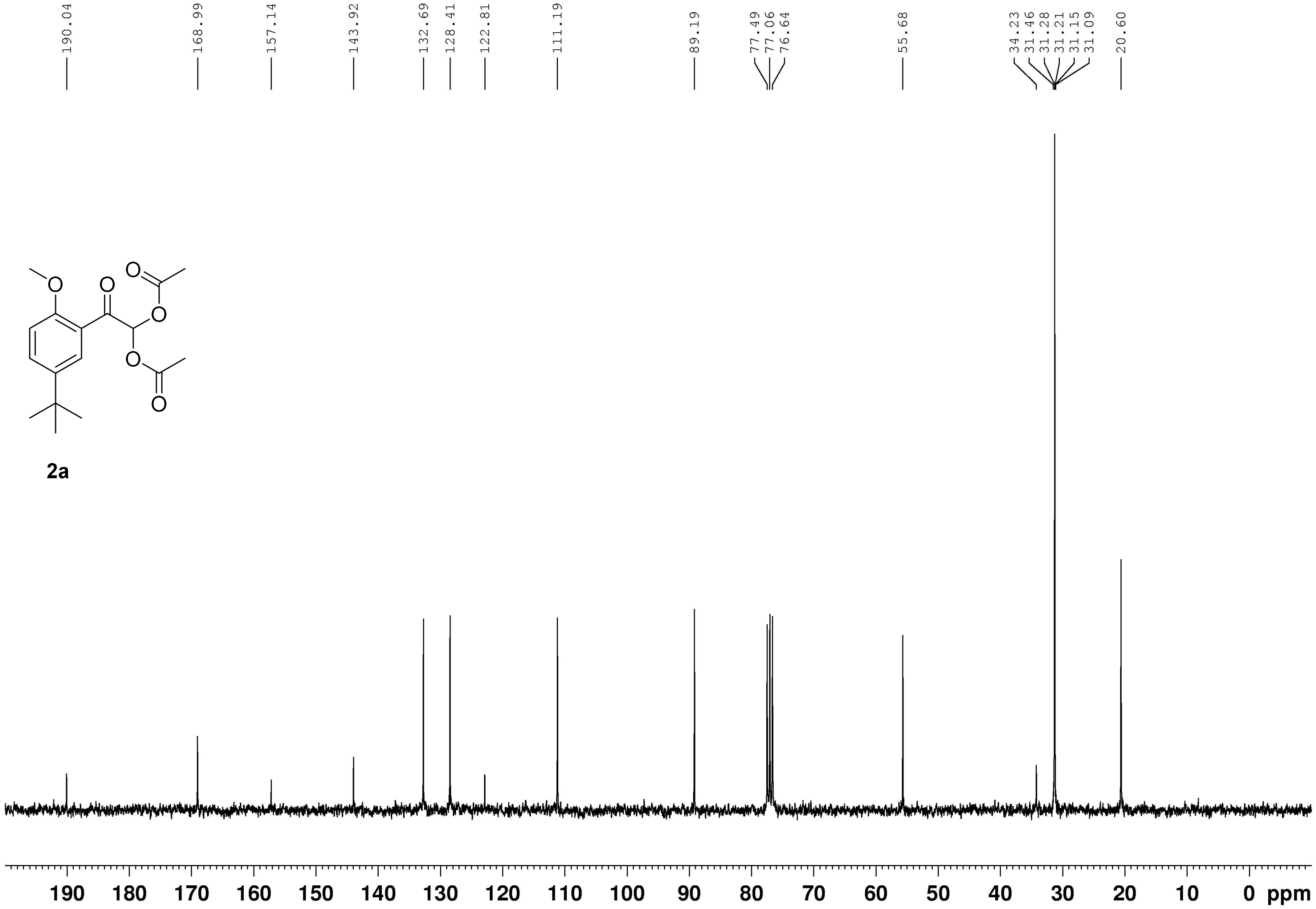

$^{13}C\{^{1}H\}$ NMR of compound **2a** (75 MHz, $CDCl_3$)

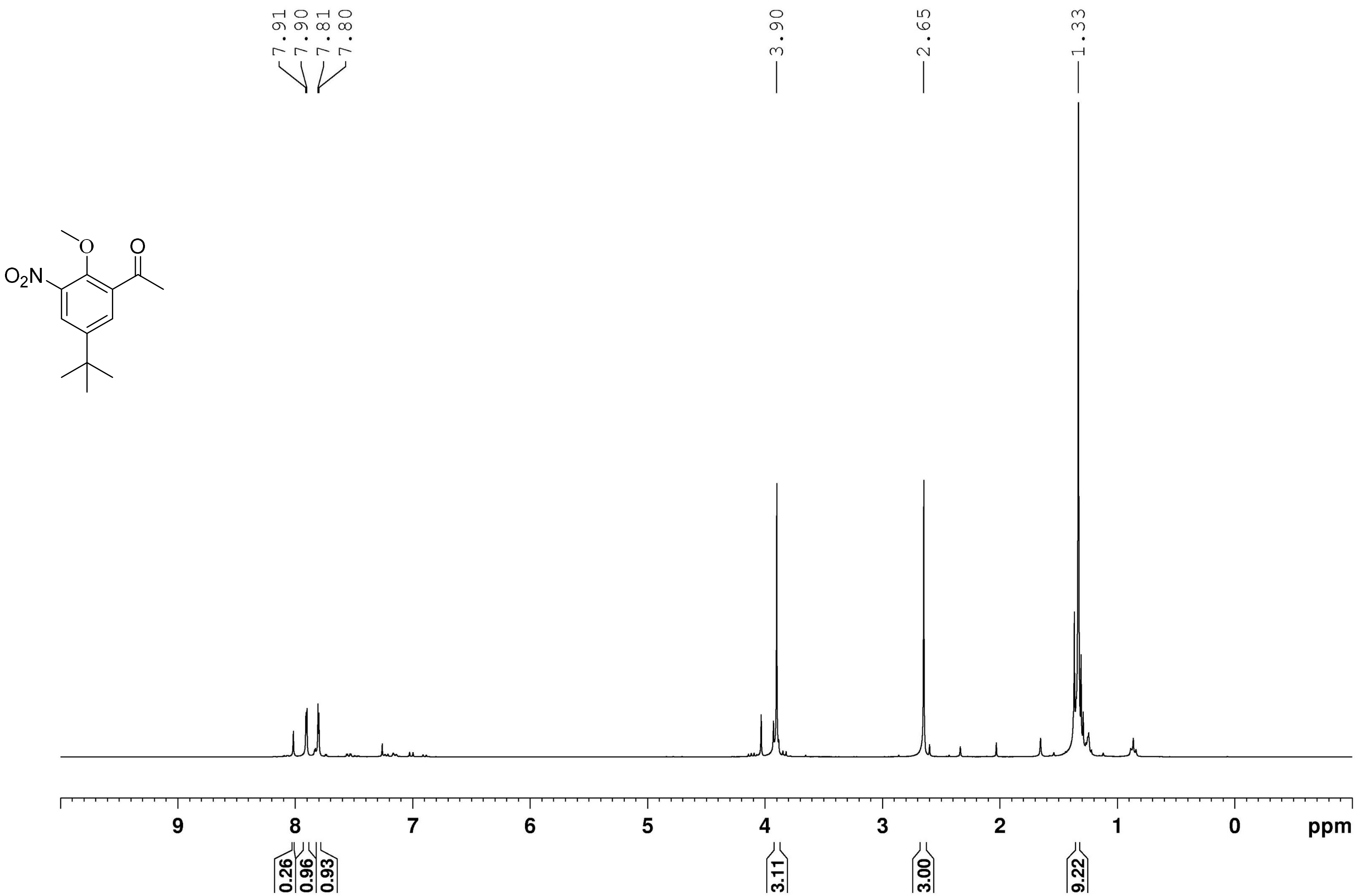

^{1}H NMR of 1-(5-(*tert*-butyl)-2-methoxy-3-nitrophenyl)ethan-1-one (300 MHz, $CDCl_3$)

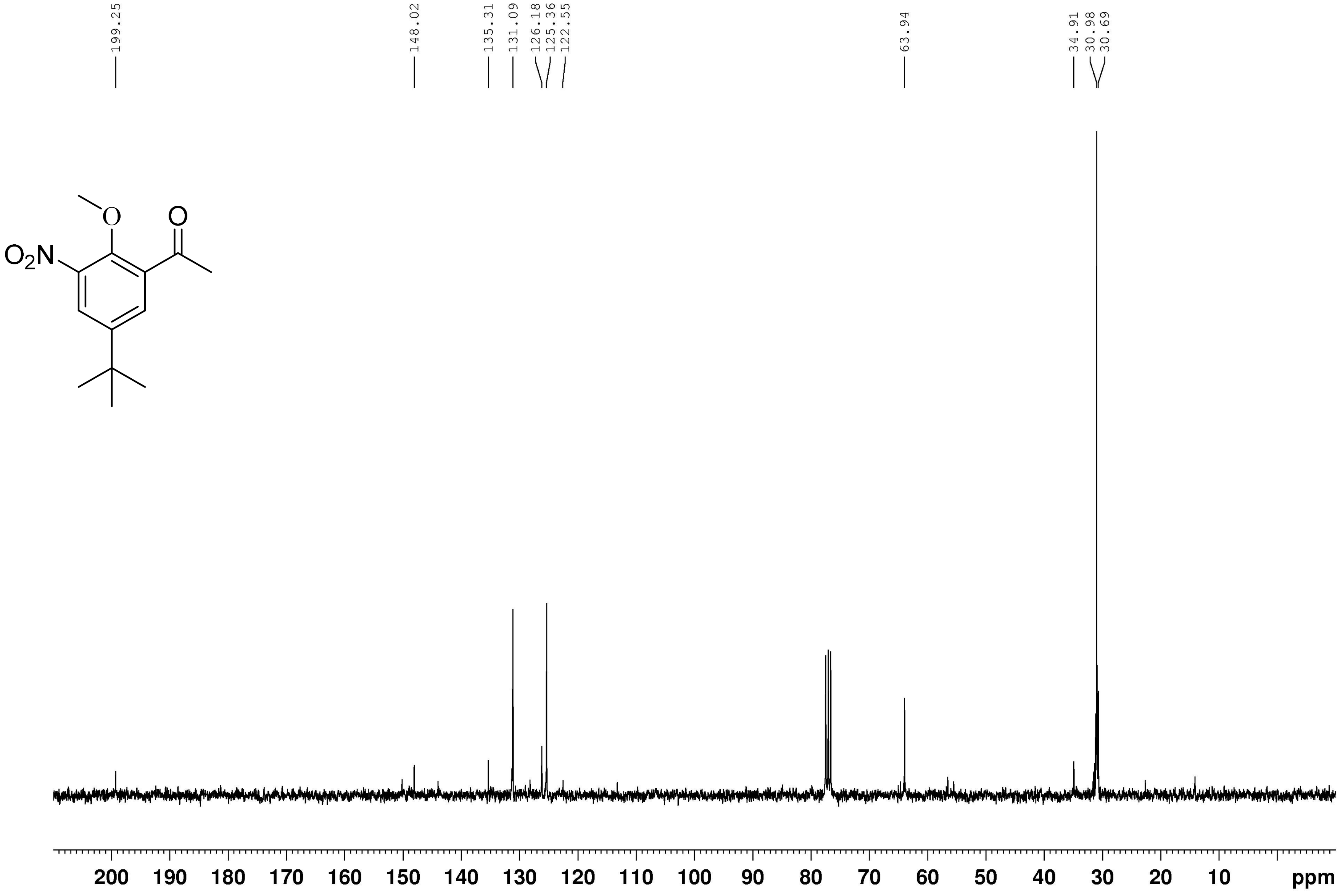

^{13}C NMR of 1-(5-(*tert*-butyl)-2-methoxy-3-nitrophenyl)ethan-1-one (75 MHz, $CDCl_3$)

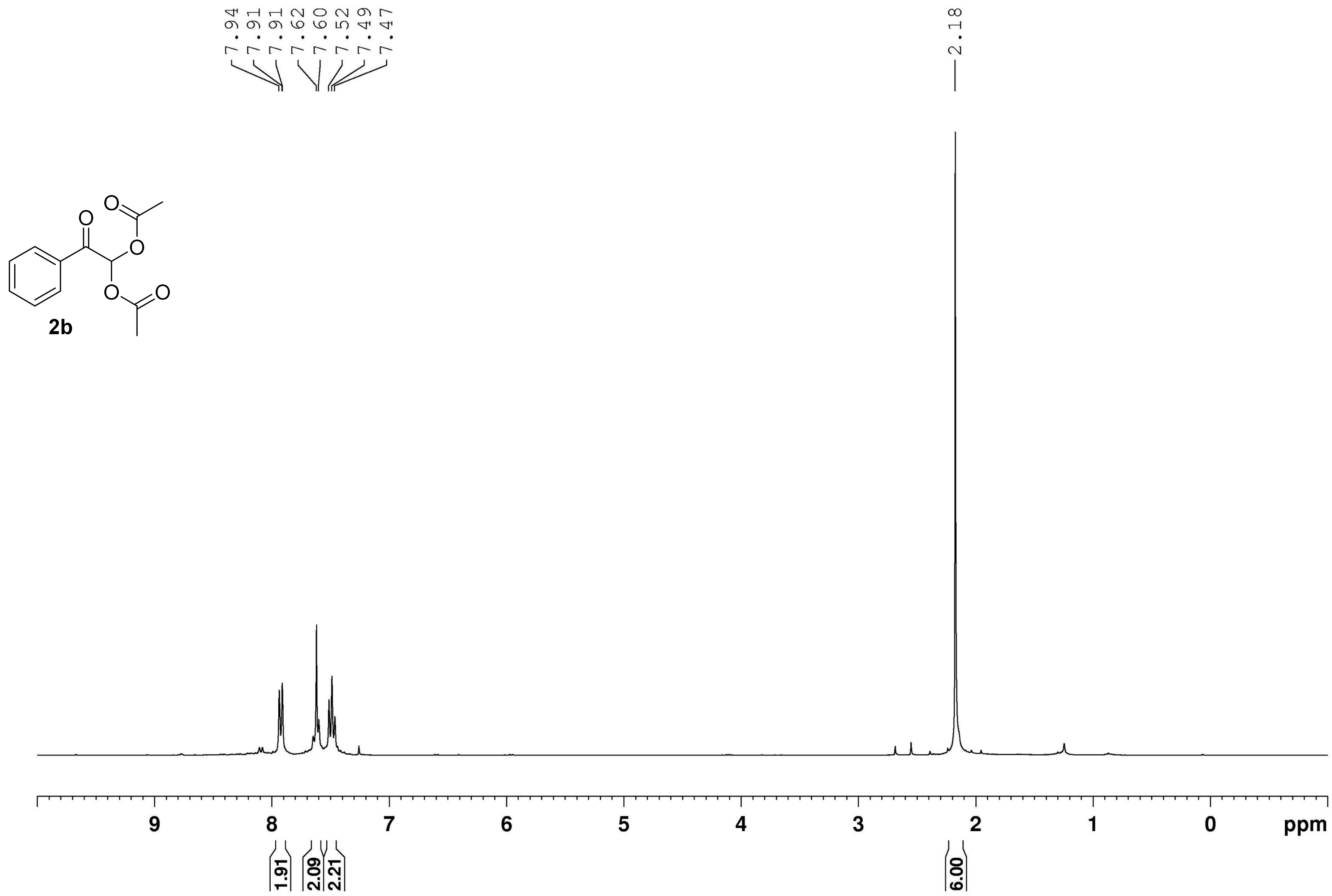

^{1}H NMR of compound **2b** (300 MHz, $CDCl_3$)

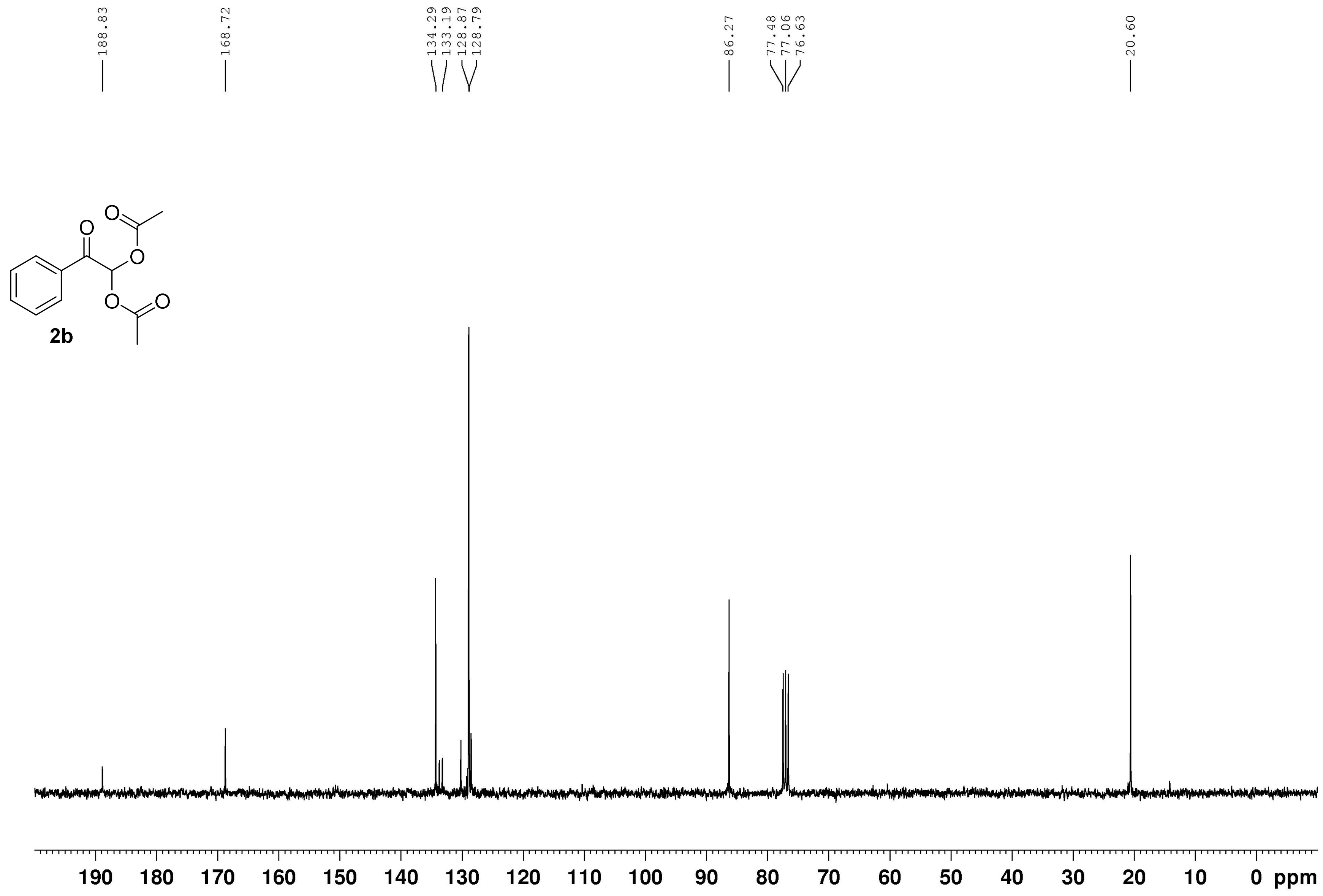

$^{13}C\{^{1}H\}$ NMR of compound **2b** (75 MHz, $CDCl_3$)

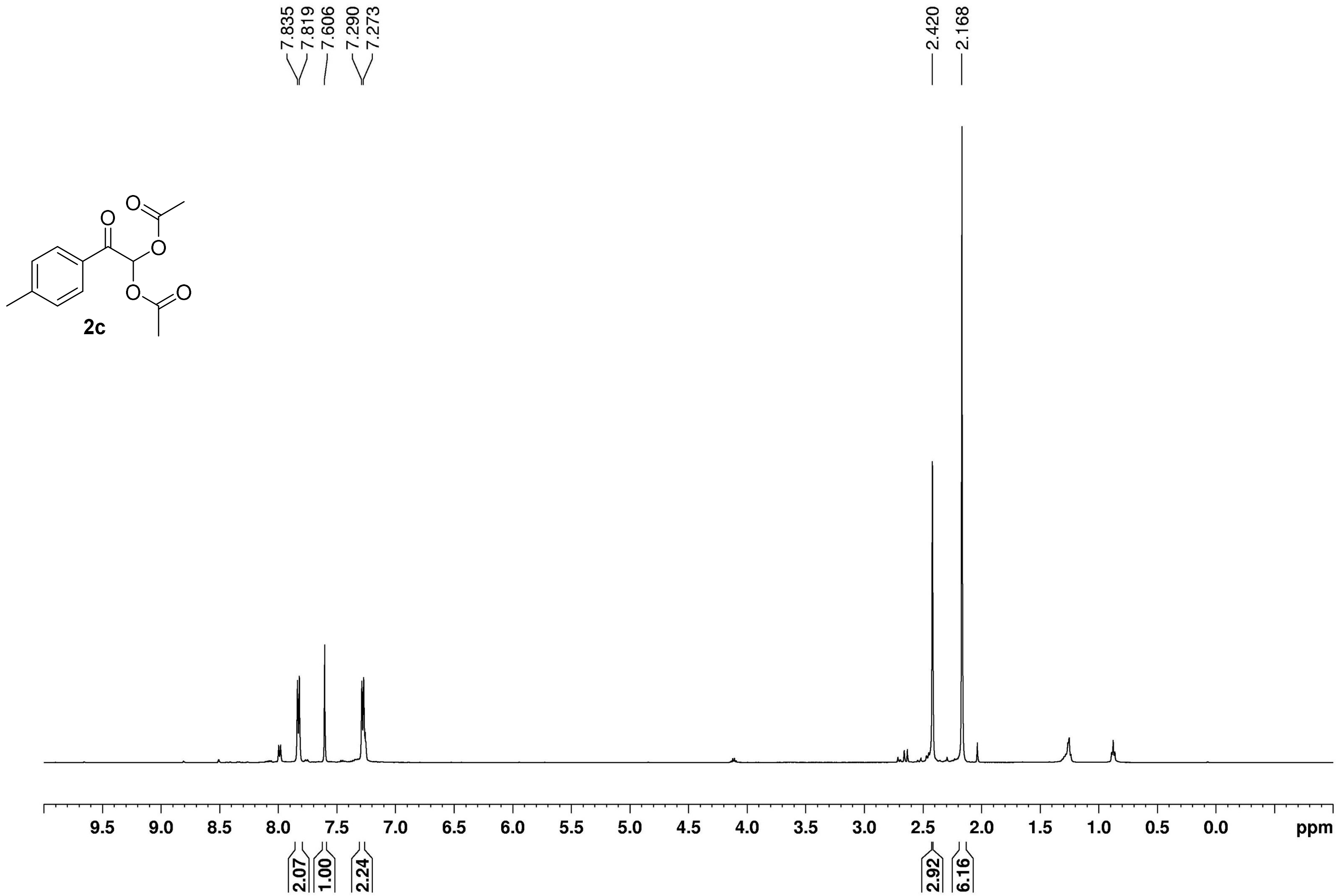

^{1}H NMR of compound **2c** (500 MHz, $CDCl_3$)

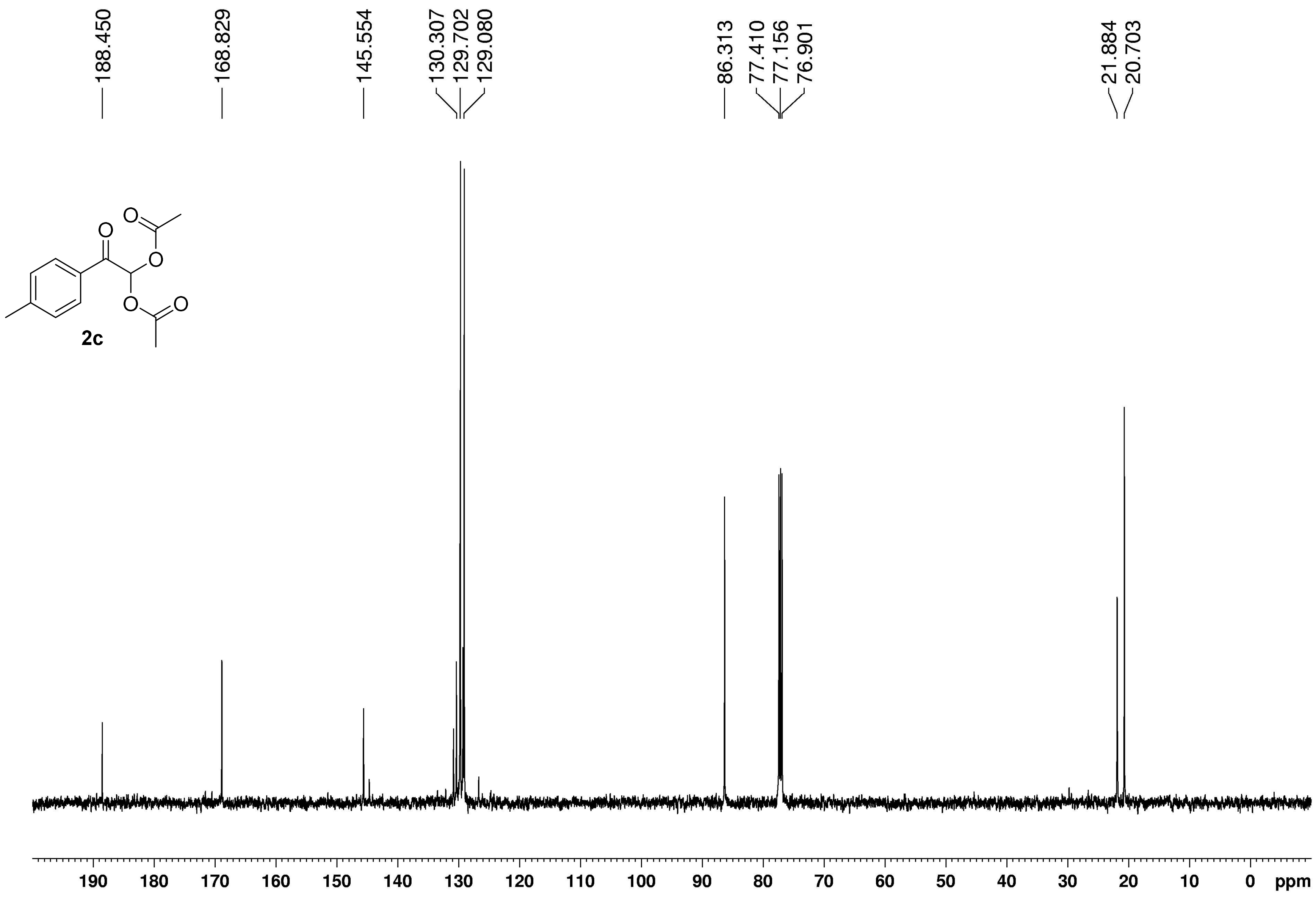

$^{13}C\{^1H\}$ NMR of compound **2c** (126 MHz, $CDCl_3$)

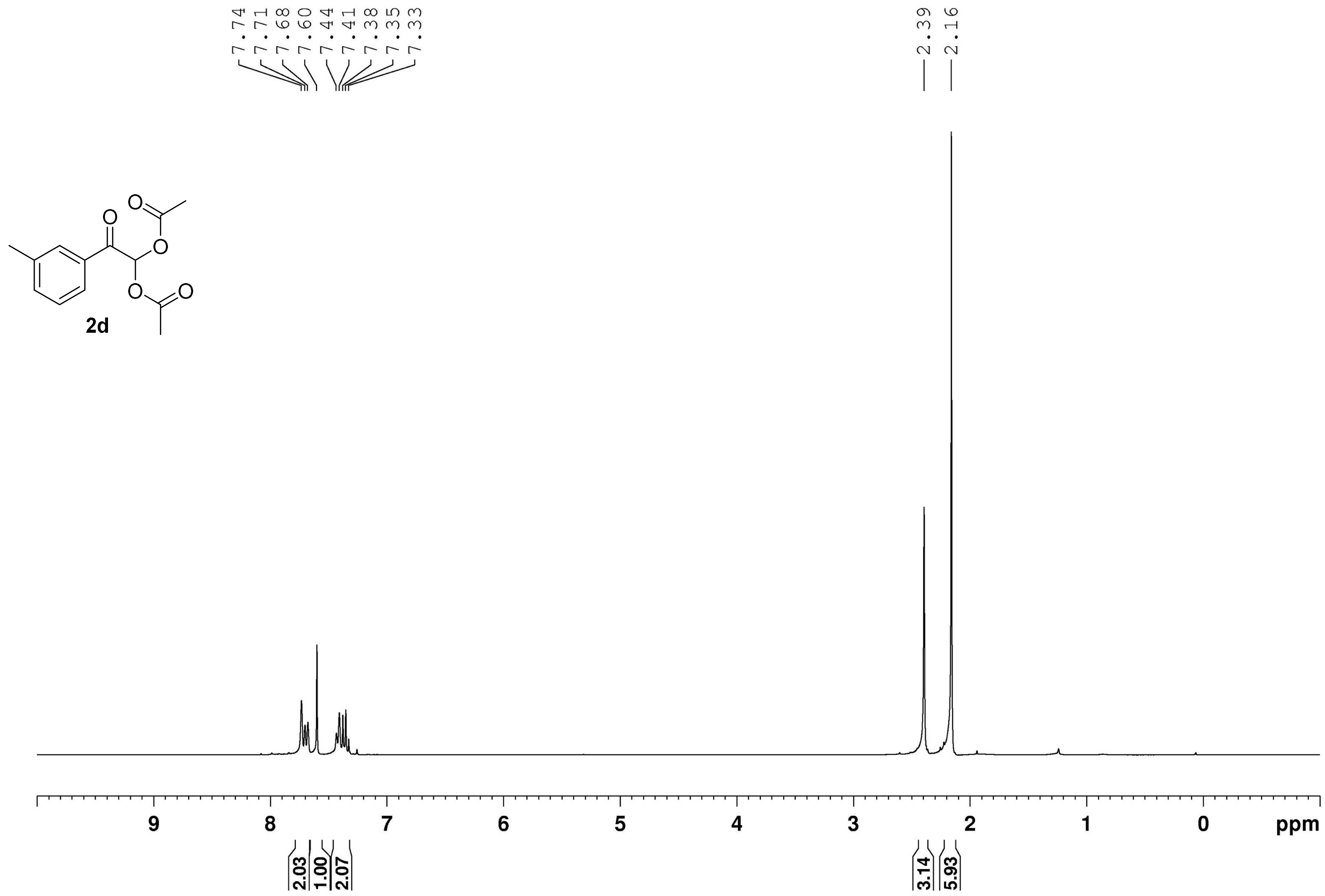

^{1}H NMR of compound **2d** (300 MHz, $CDCl_3$)

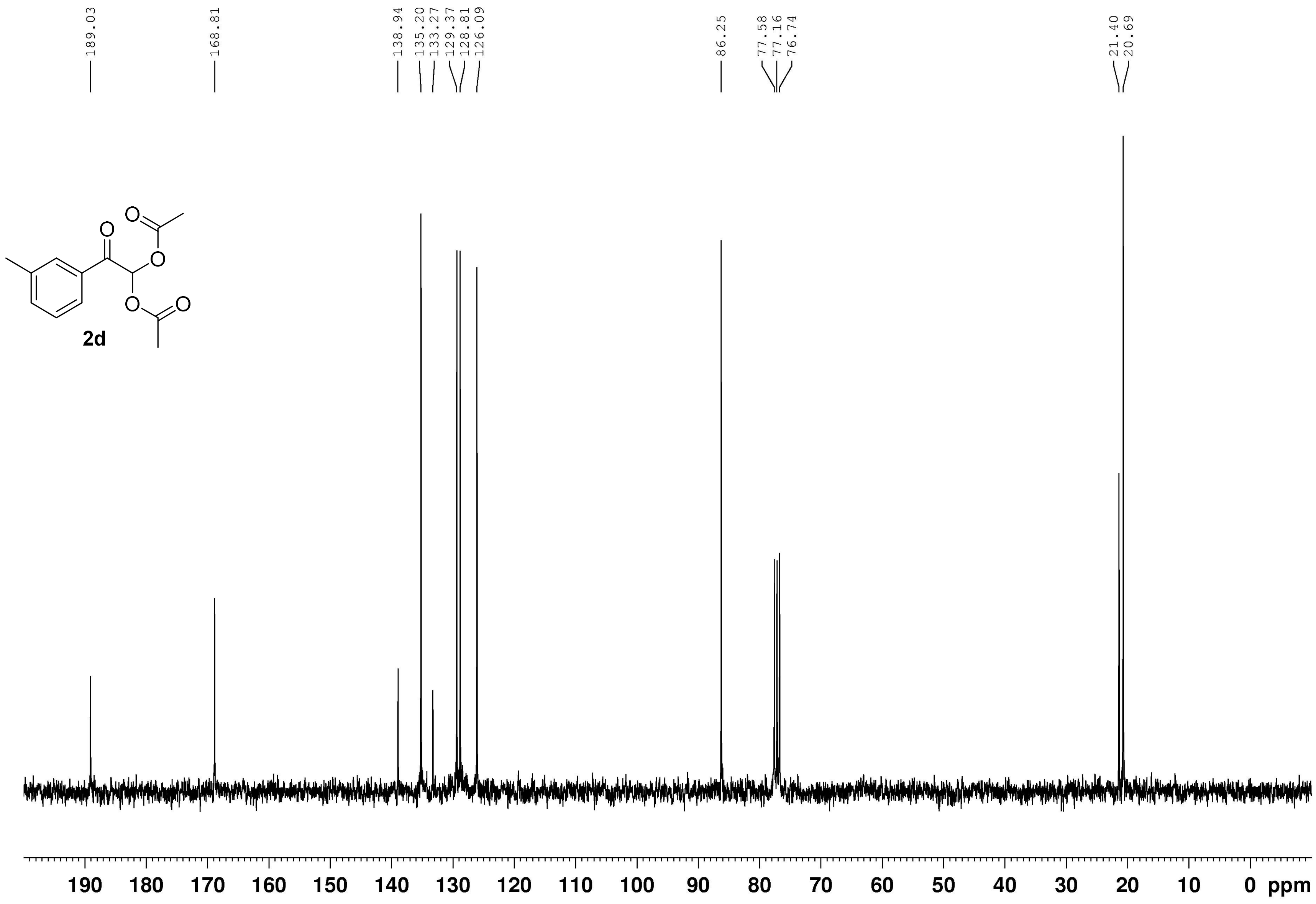

$^{13}C\{^{1}H\}$ NMR of compound **2d** (75 MHz, $CDCl_3$)

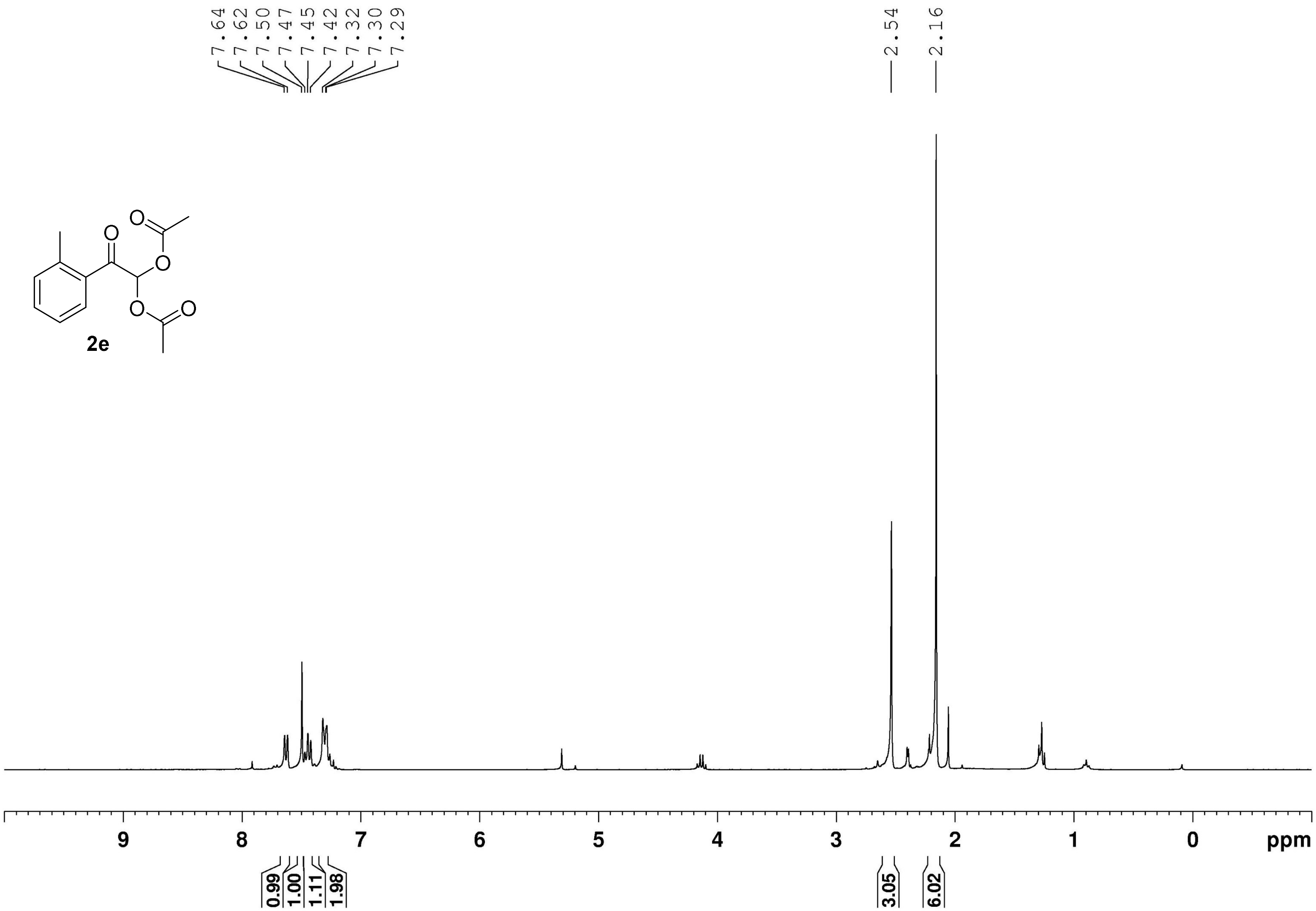

^{1}H NMR of compound **2e** (300 MHz, $CDCl_3$)

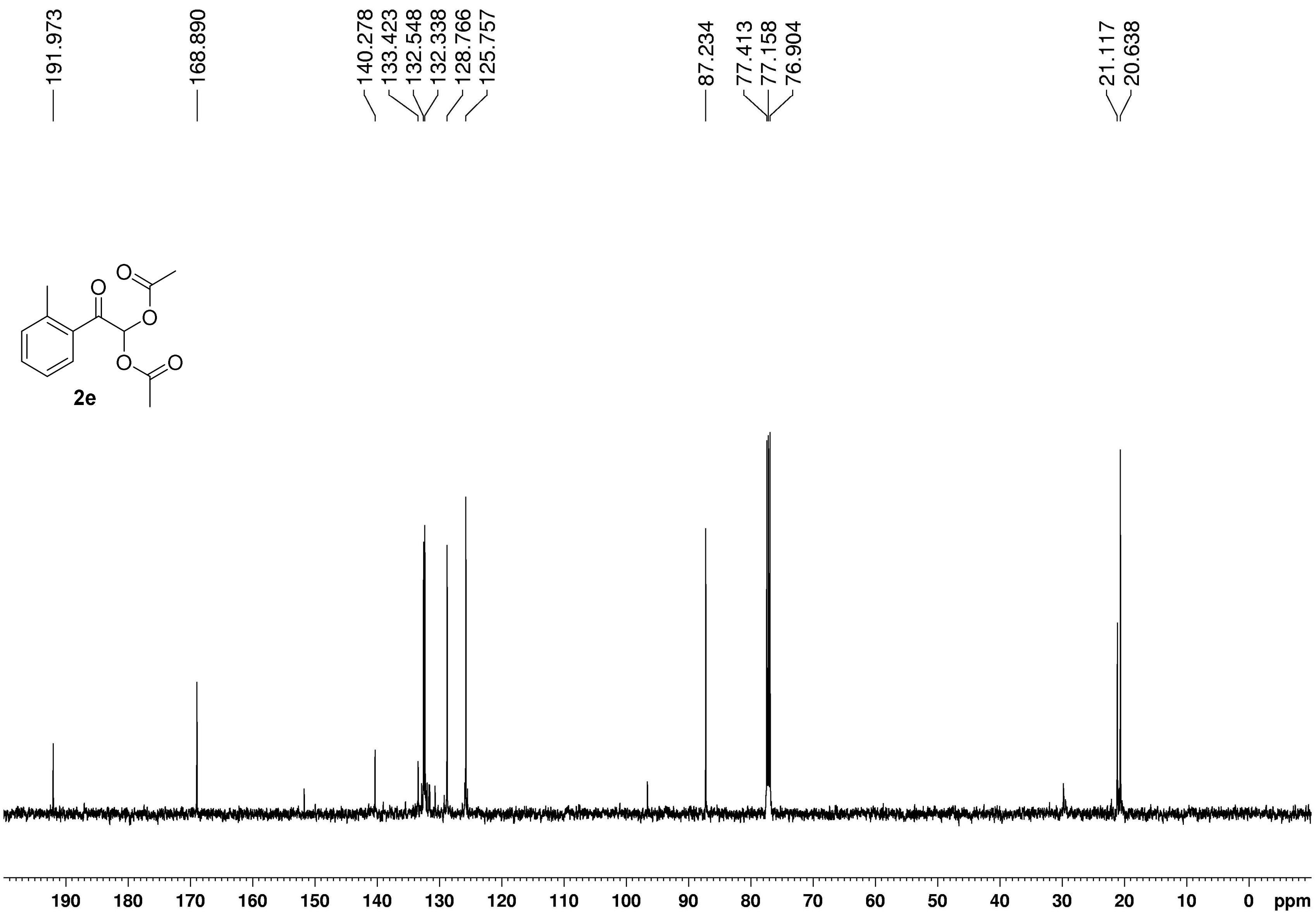

$^{13}C\{^{1}H\}$ NMR of compound **2e** (126 MHz, $CDCl_3$)

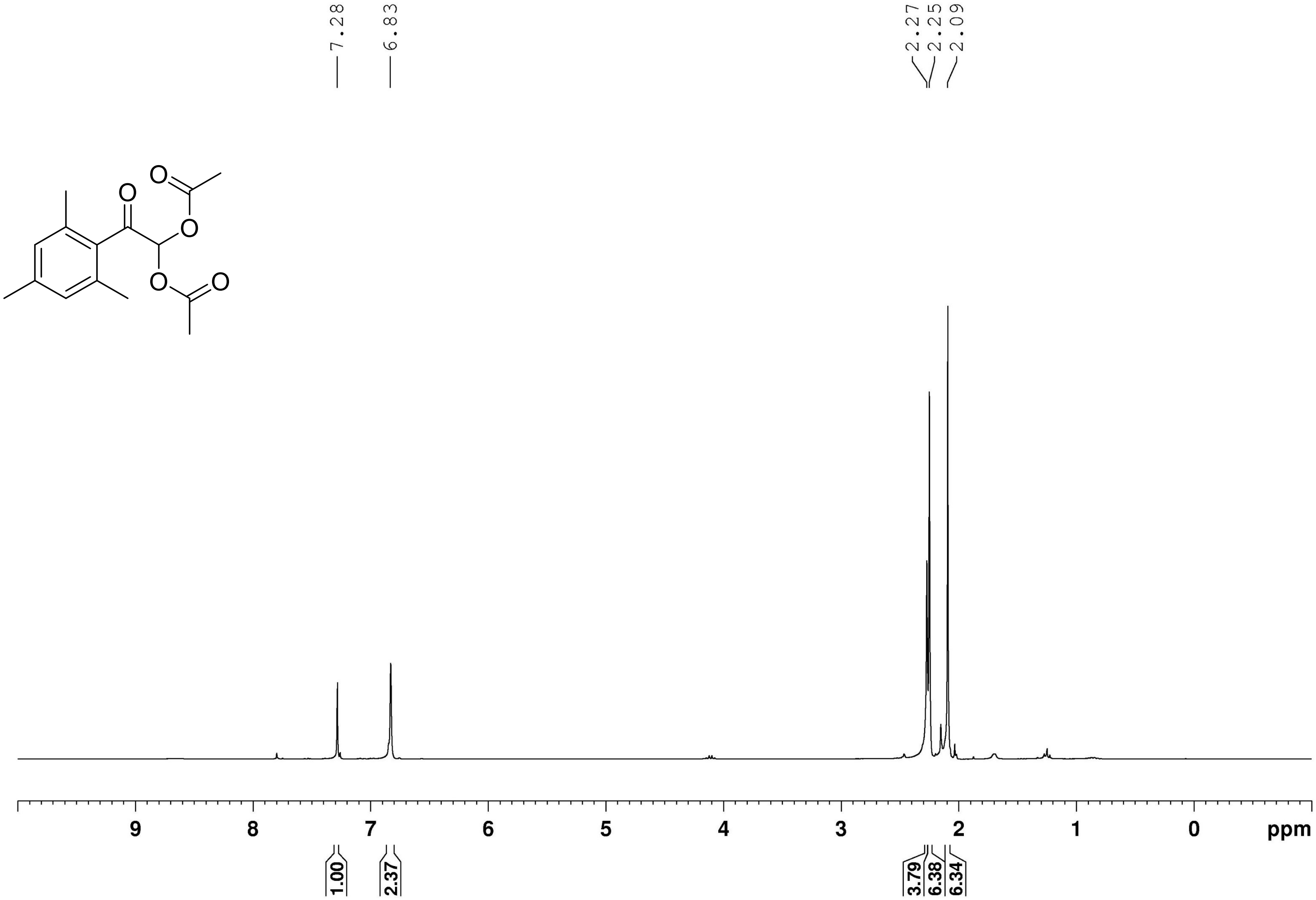

^{1}H NMR of compound **2f** (300 MHz, $CDCl_3$)

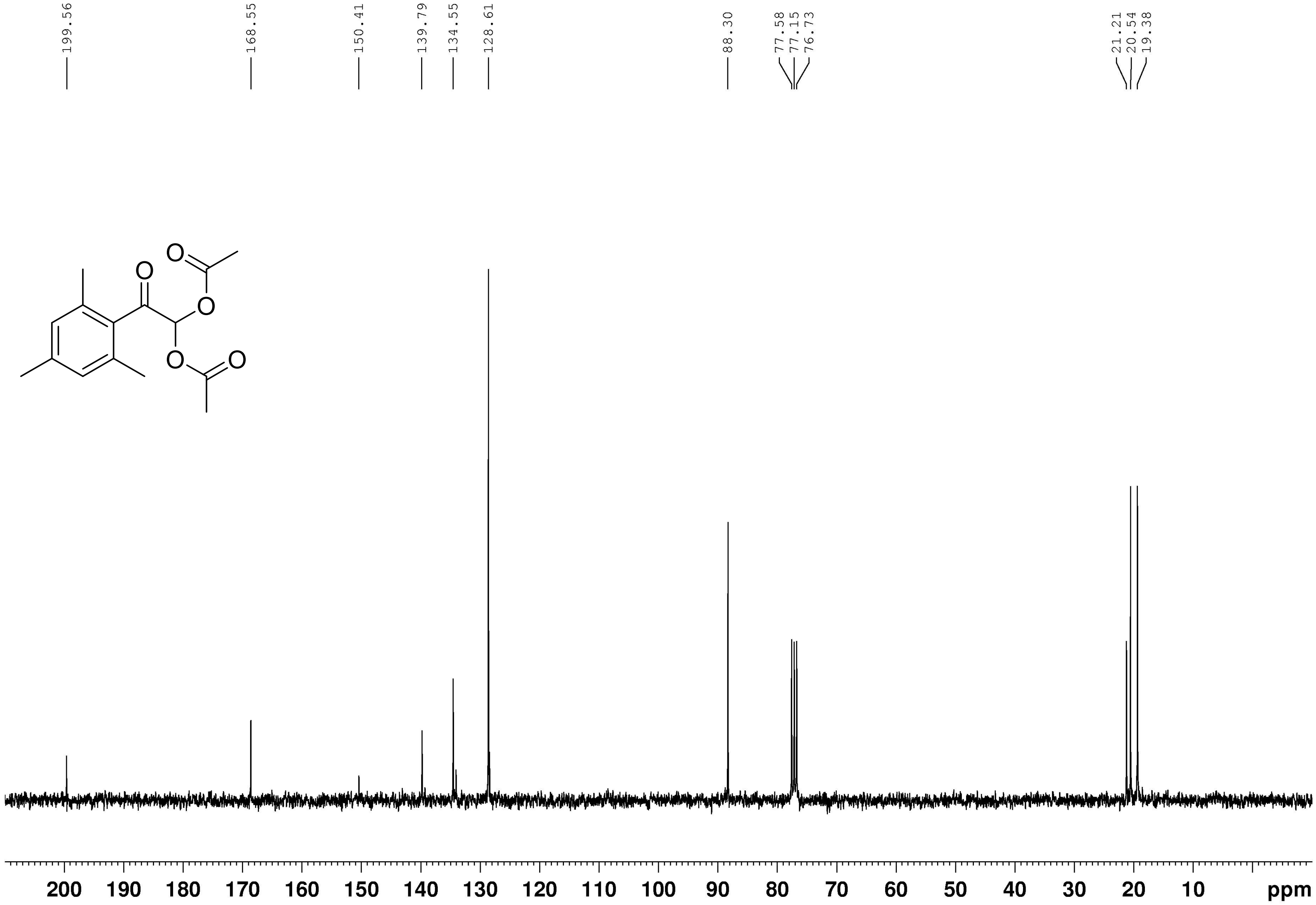

$^{13}C\{^{1}H\}$ NMR of compound **2f** (75 MHz, $CDCl_3$)

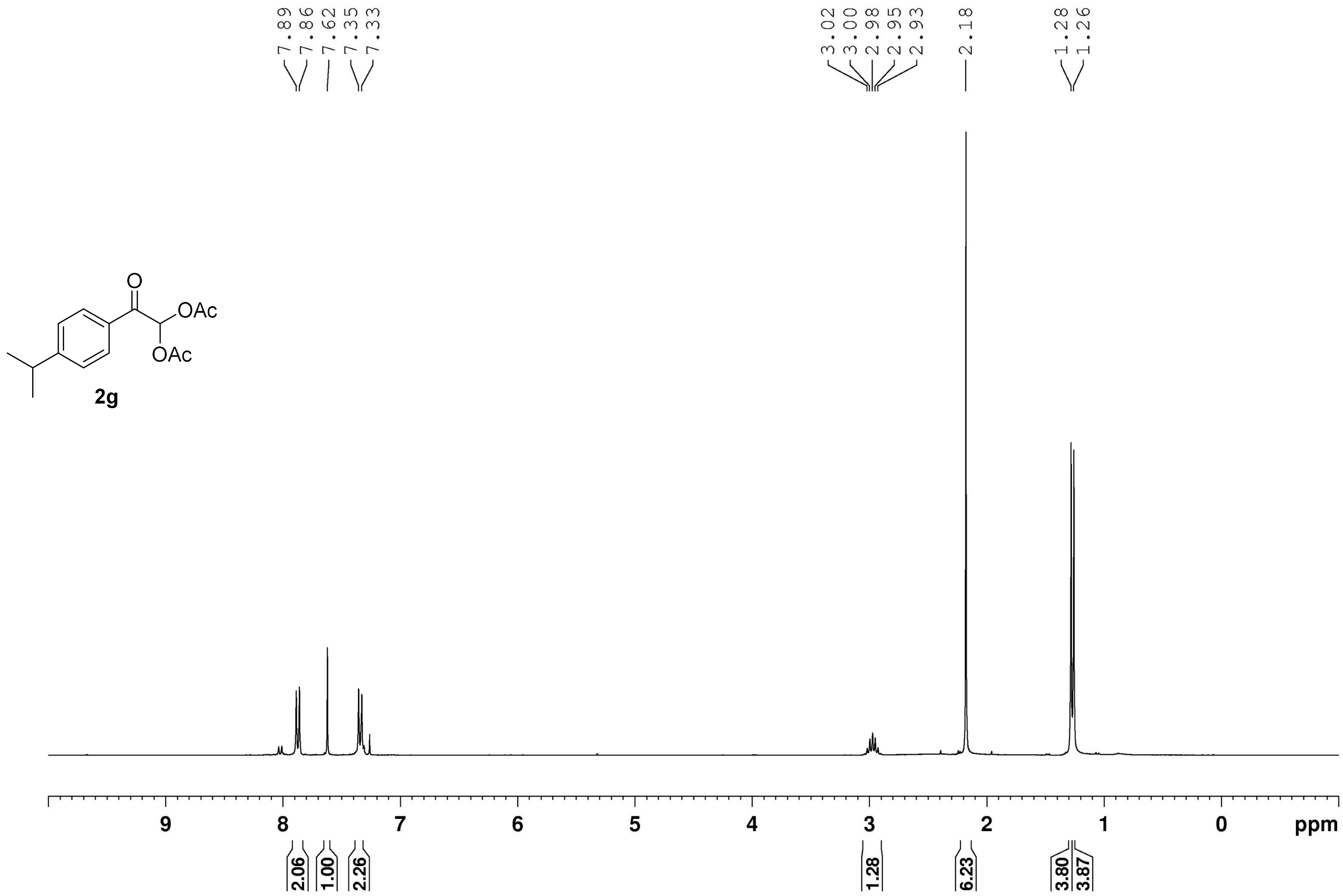

^{1}H NMR of compound **2g** (300 MHz, $CDCl_3$)

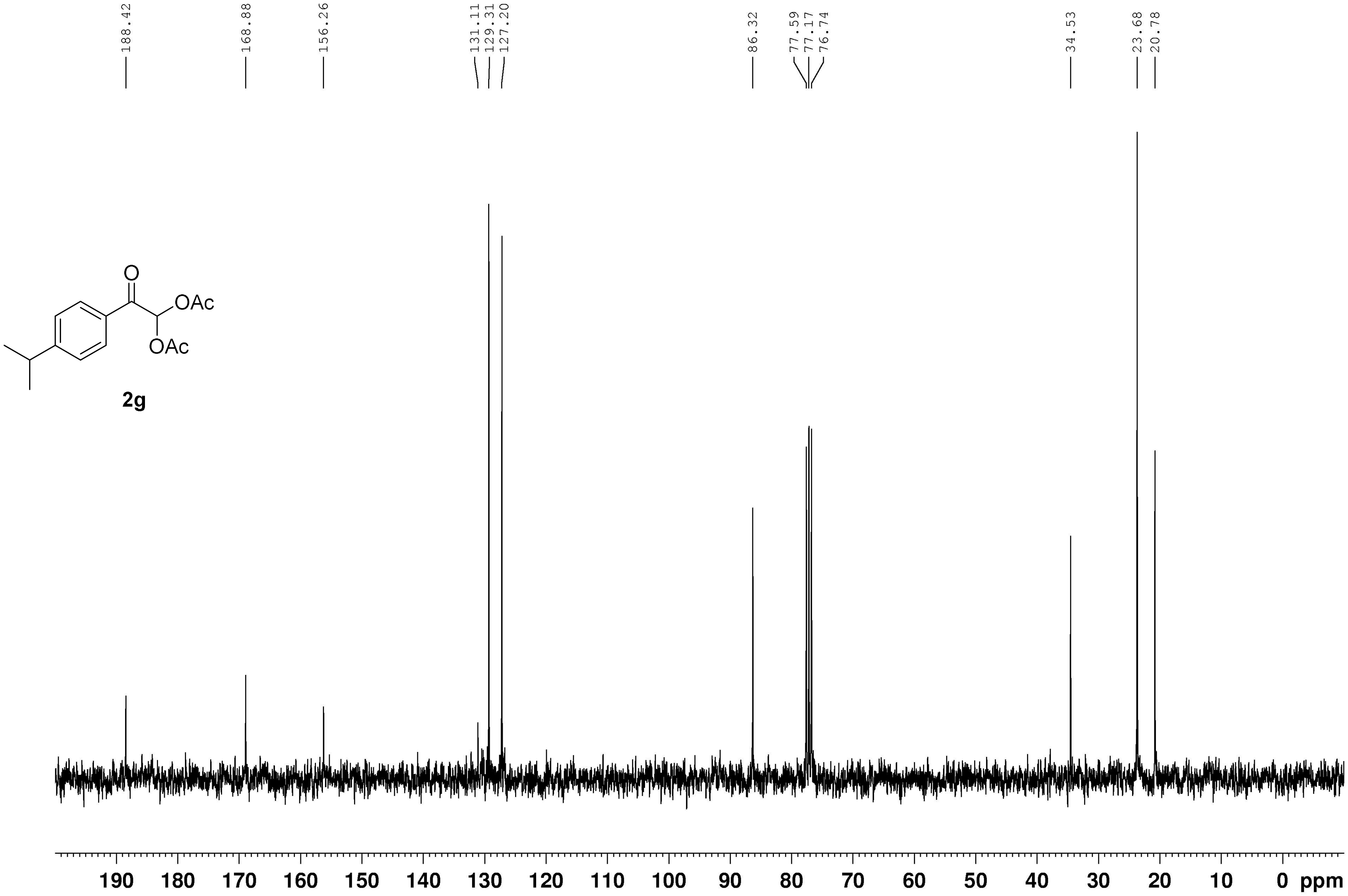

$^{13}C\{^1H\}$ NMR of compound **2g** (75 MHz, $CDCl_3$)

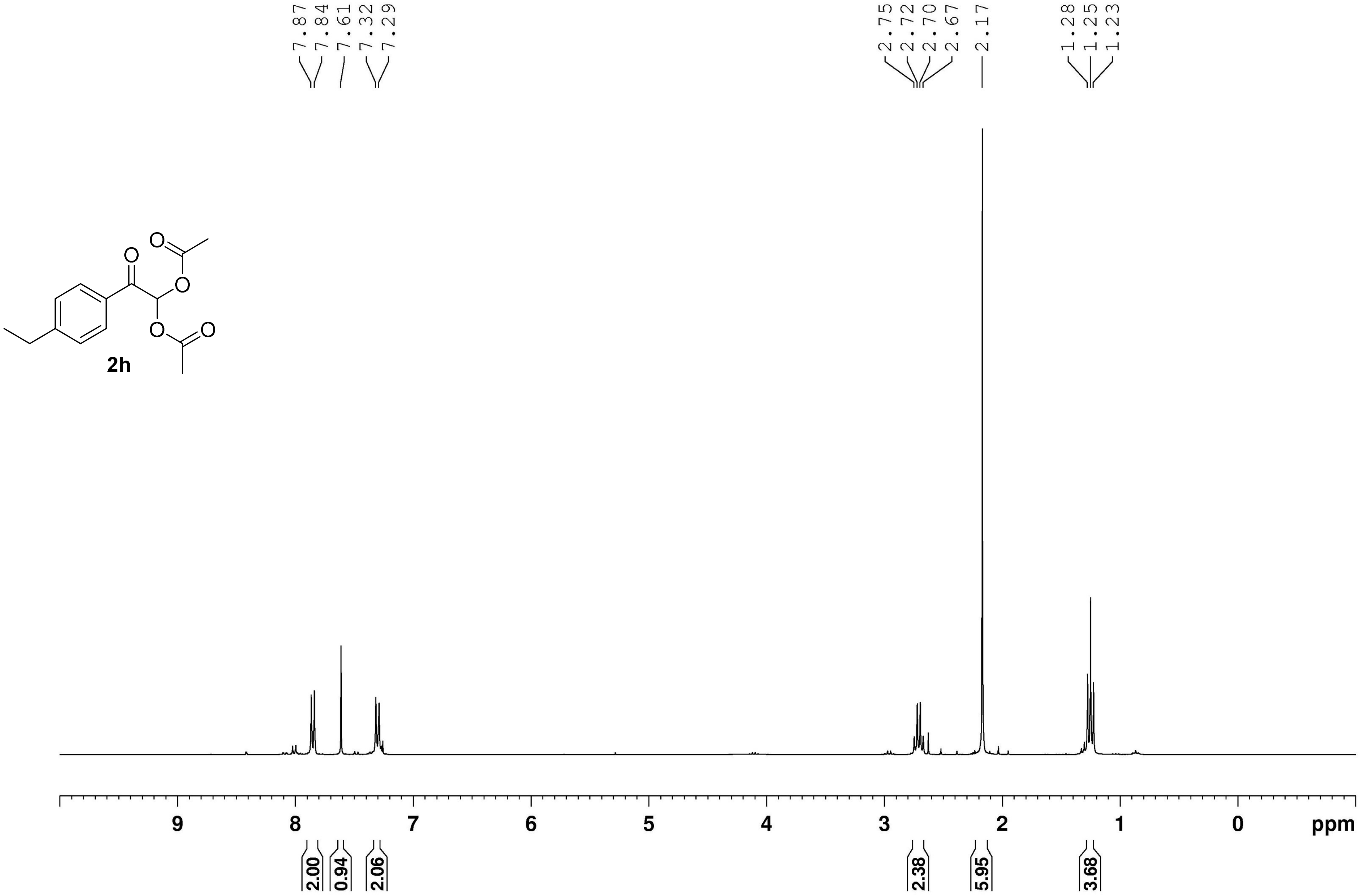

^{1}H NMR of compound **2h** (300 MHz, $CDCl_3$)

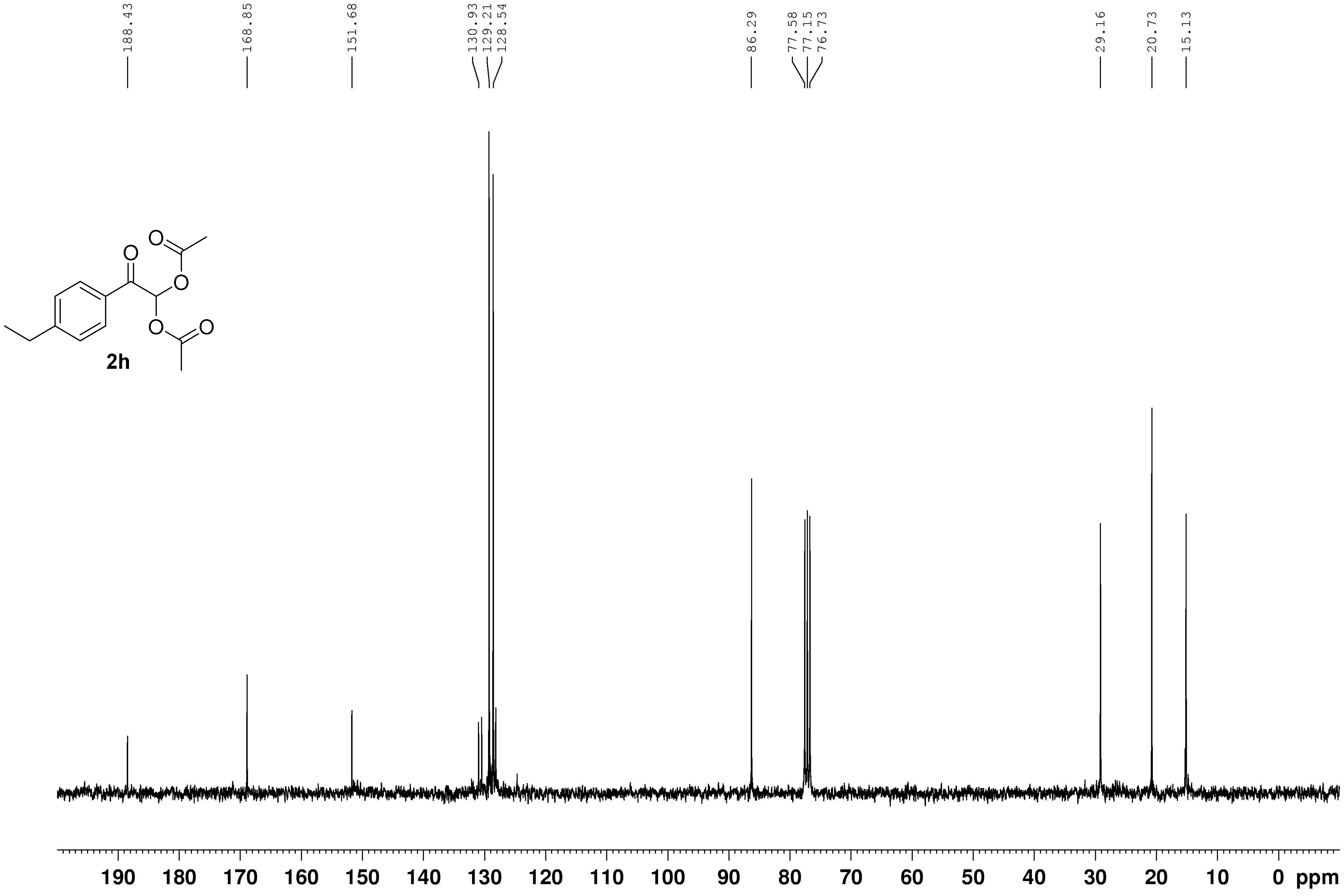

$^{13}C\{^{1}H\}$ NMR of compound **2h** (75 MHz, $CDCl_3$)

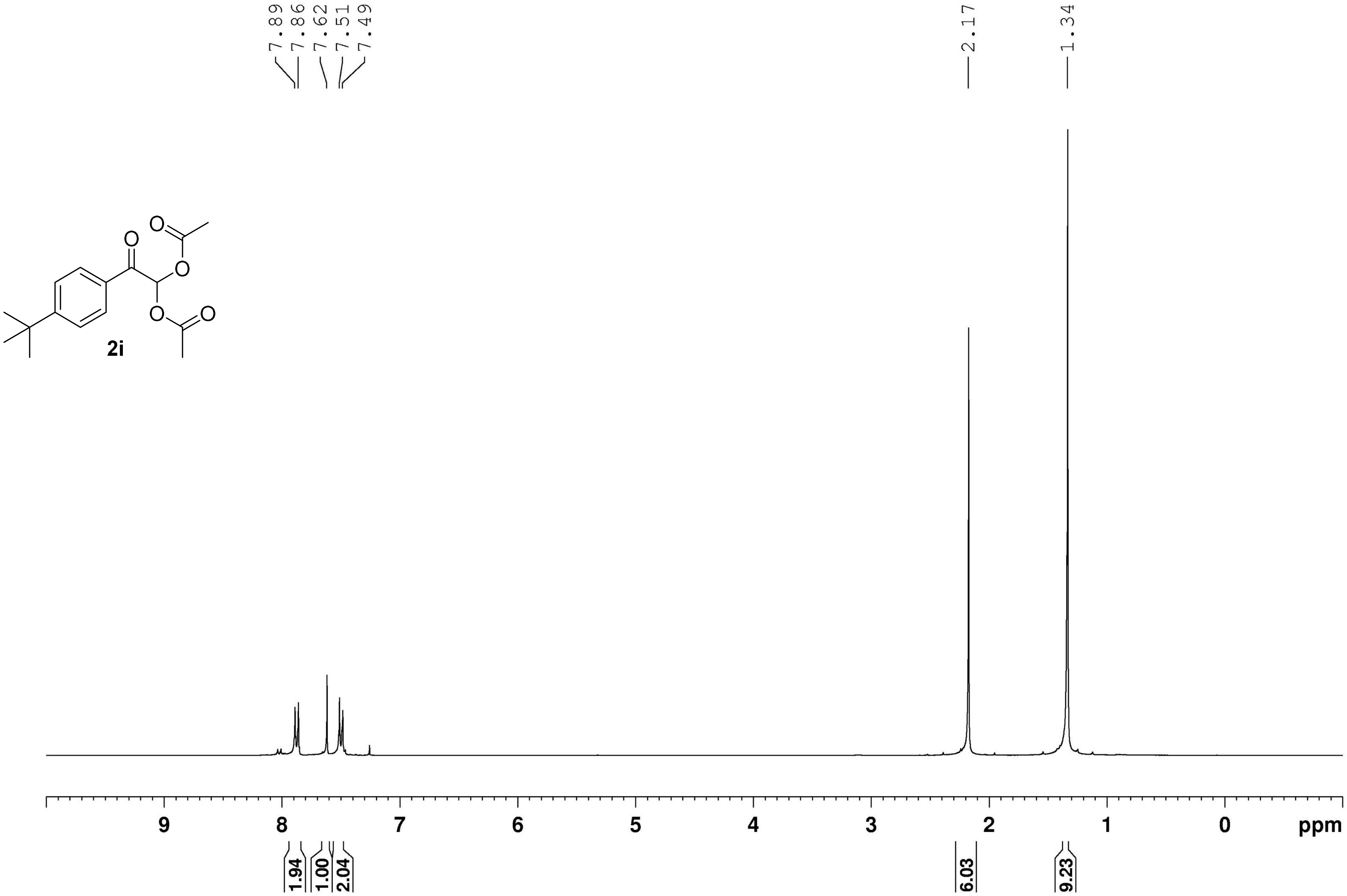

^{1}H NMR of compound **2i** (300 MHz, $CDCl_3$)

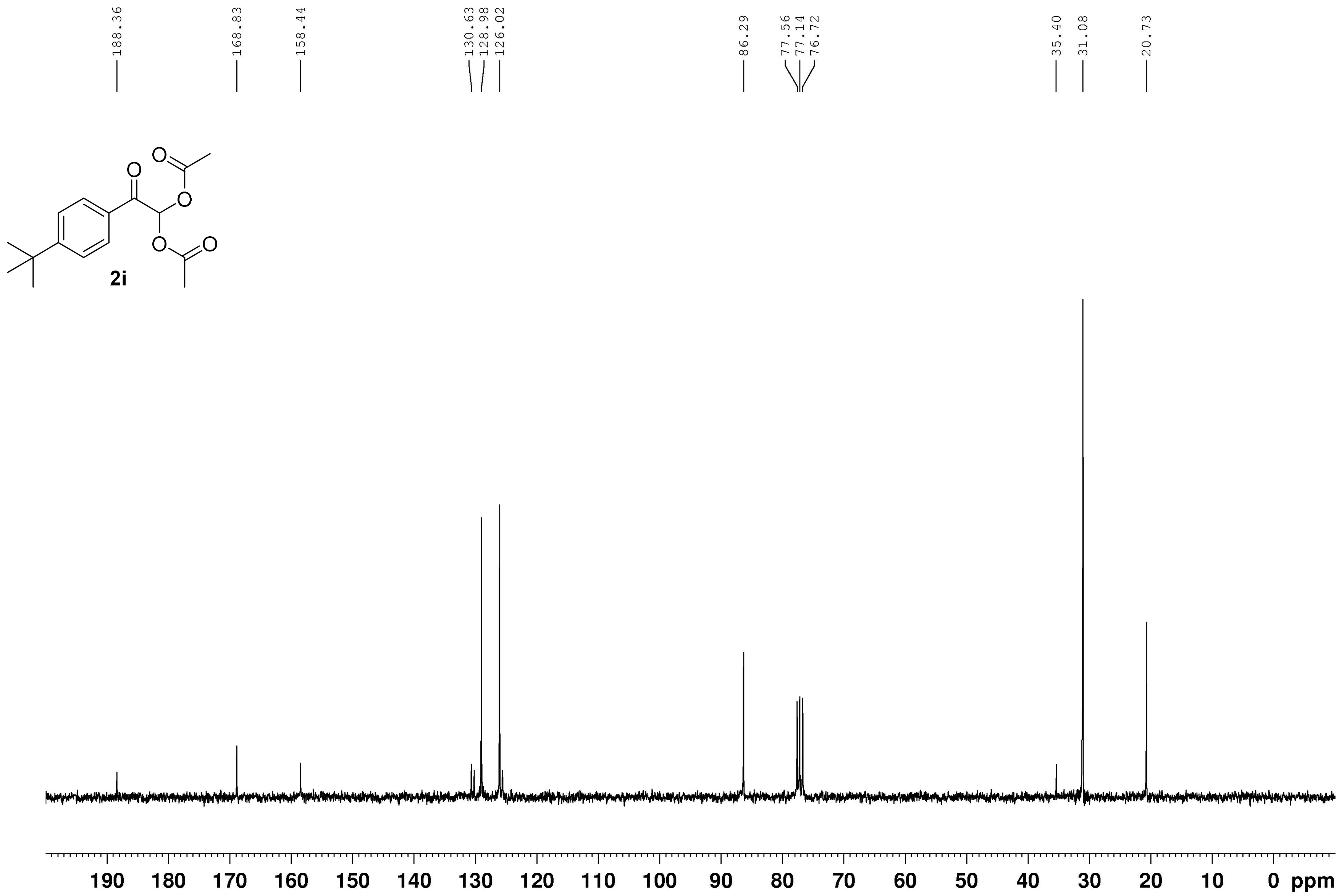

$^{13}C\{^1H\}$ NMR of compound **2i** (75 MHz, $CDCl_3$)

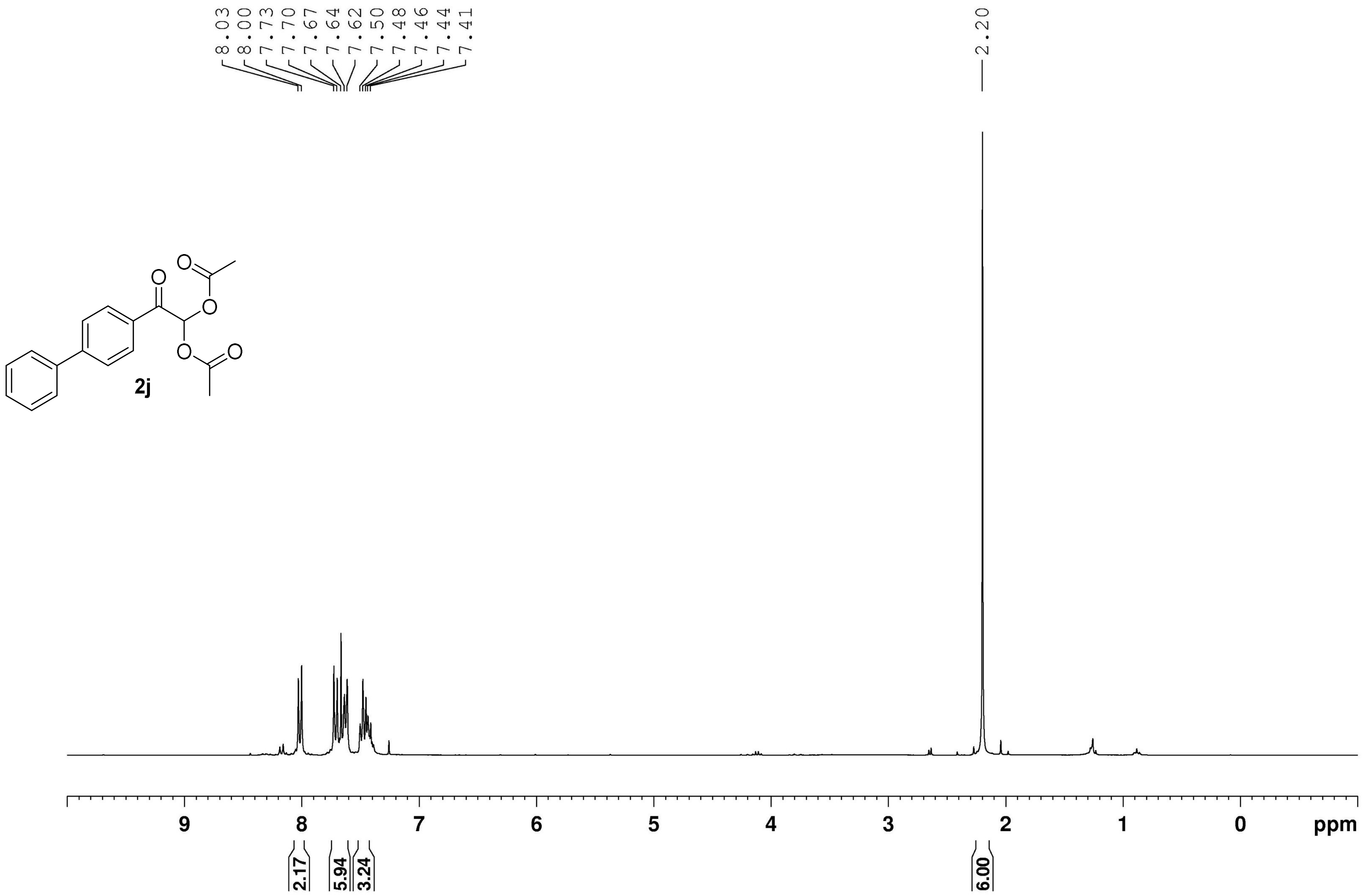

^{1}H NMR of compound **2j** (300 MHz, $CDCl_3$)

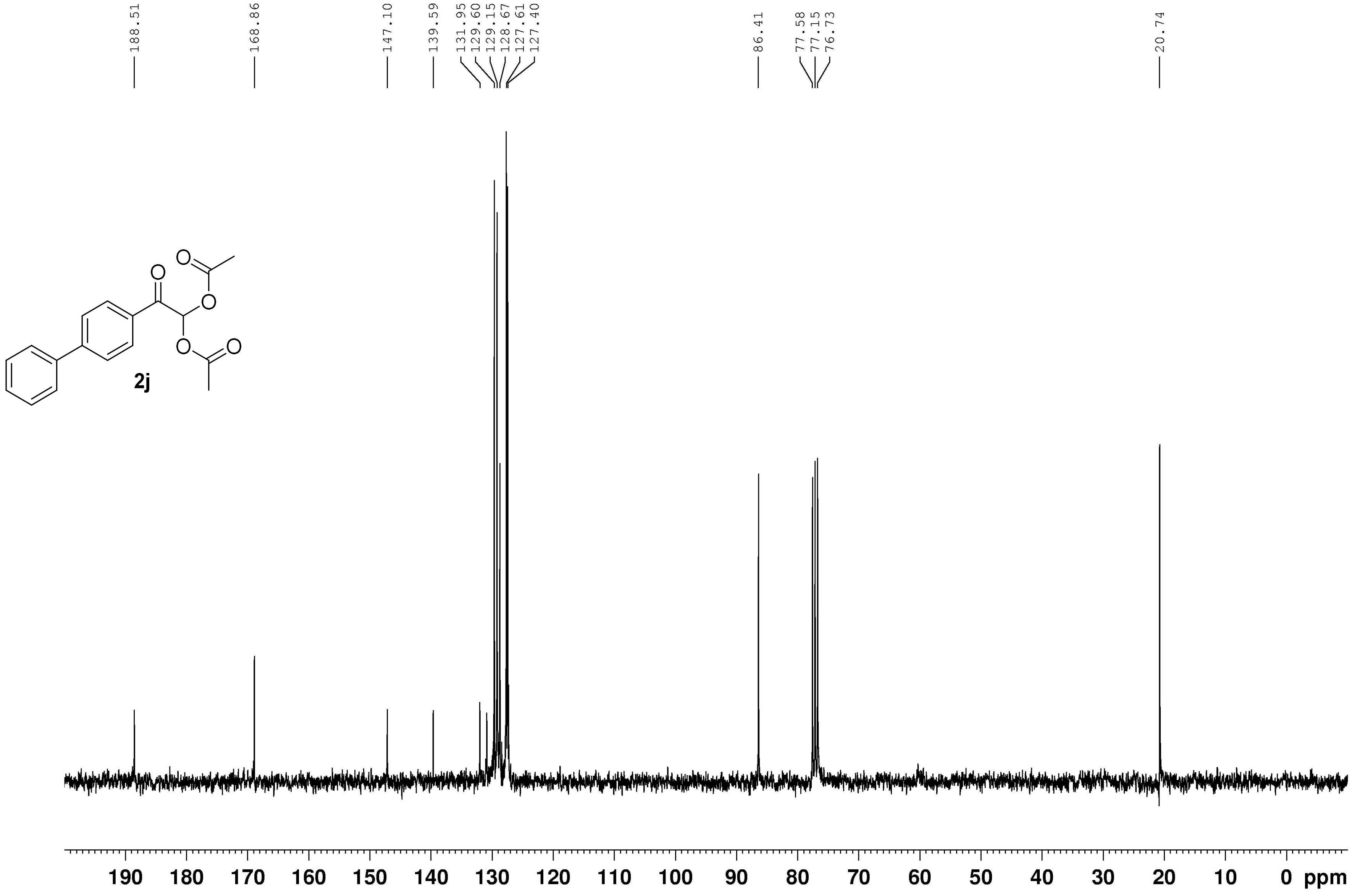

$^{13}C\{^{1}H\}$ NMR of compound **2j** (75 MHz, $CDCl_3$)

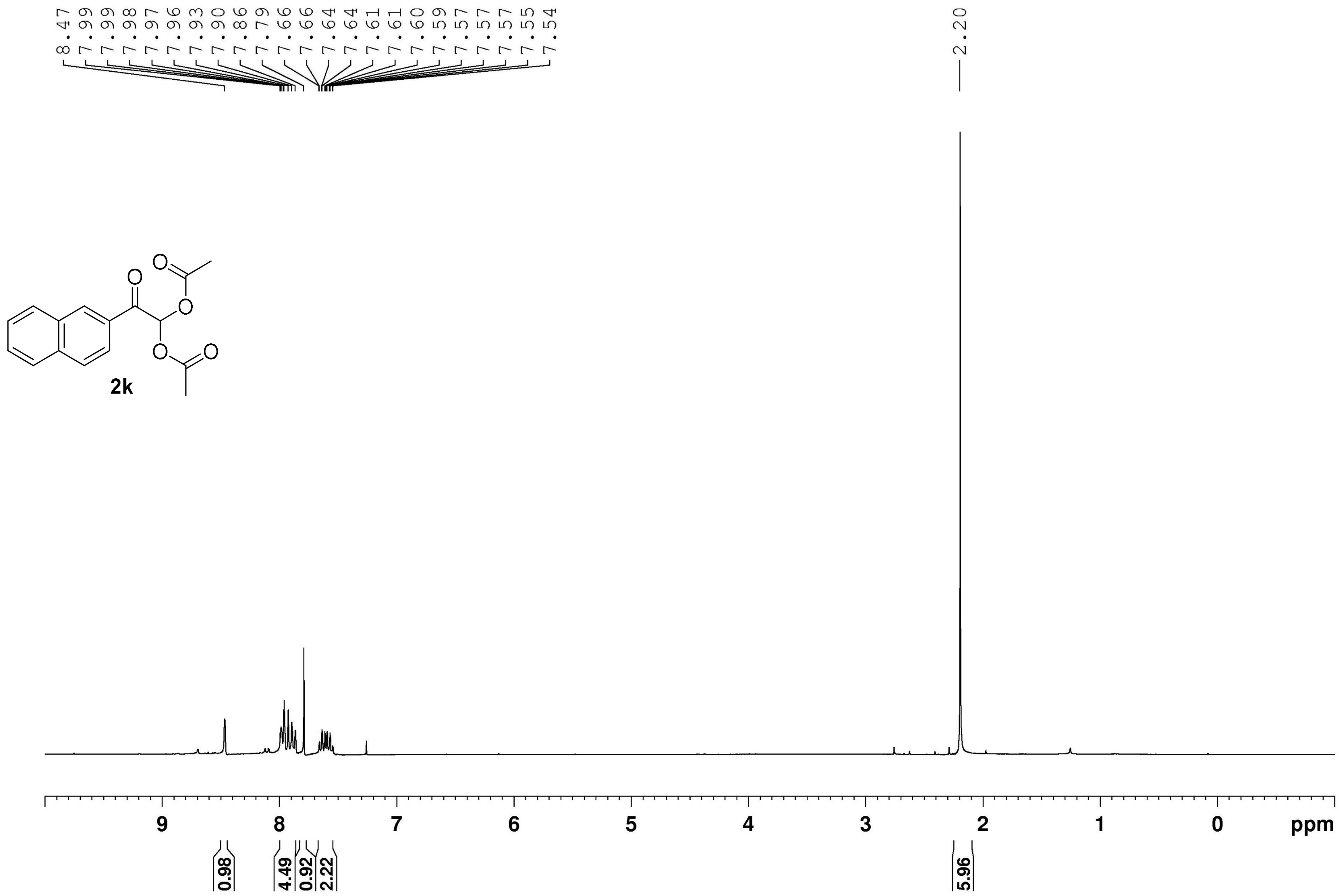

^{1}H NMR of compound **2k** (300 MHz, $CDCl_3$)

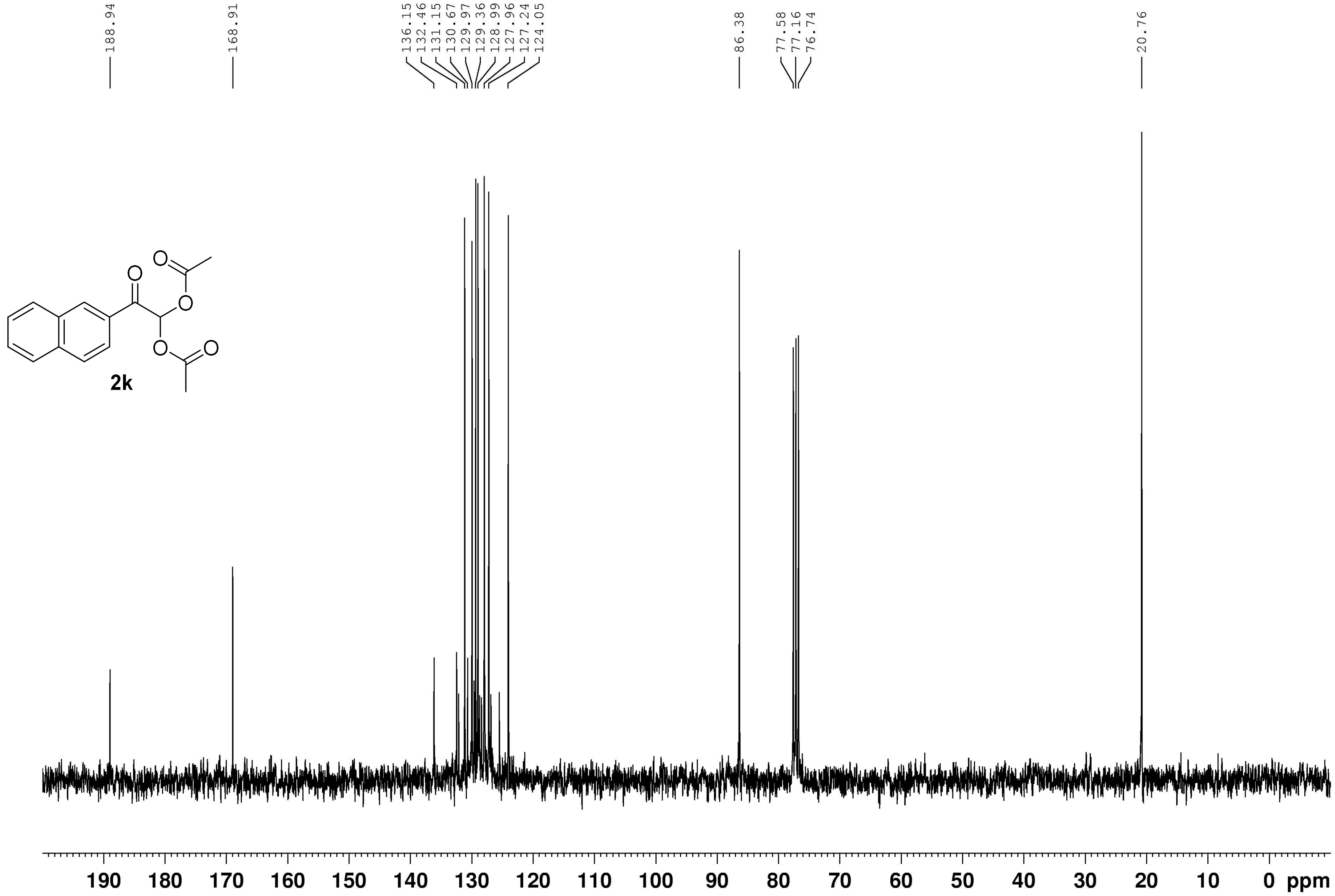

$^{13}C\{^{1}H\}$ NMR of compound **2k** (75 MHz, $CDCl_3$)

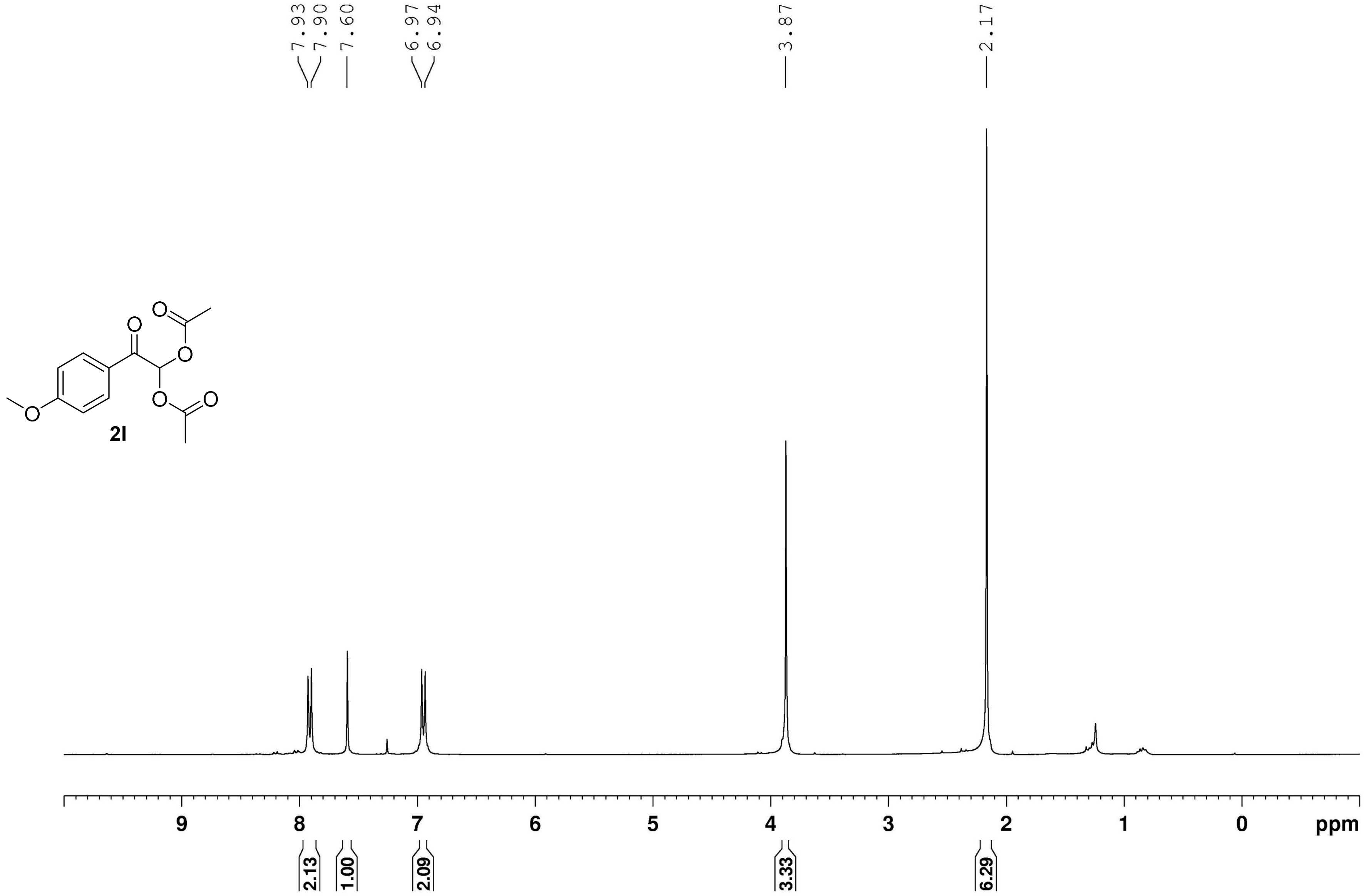

^{1}H NMR of compound **2l** (300 MHz, $CDCl_3$)

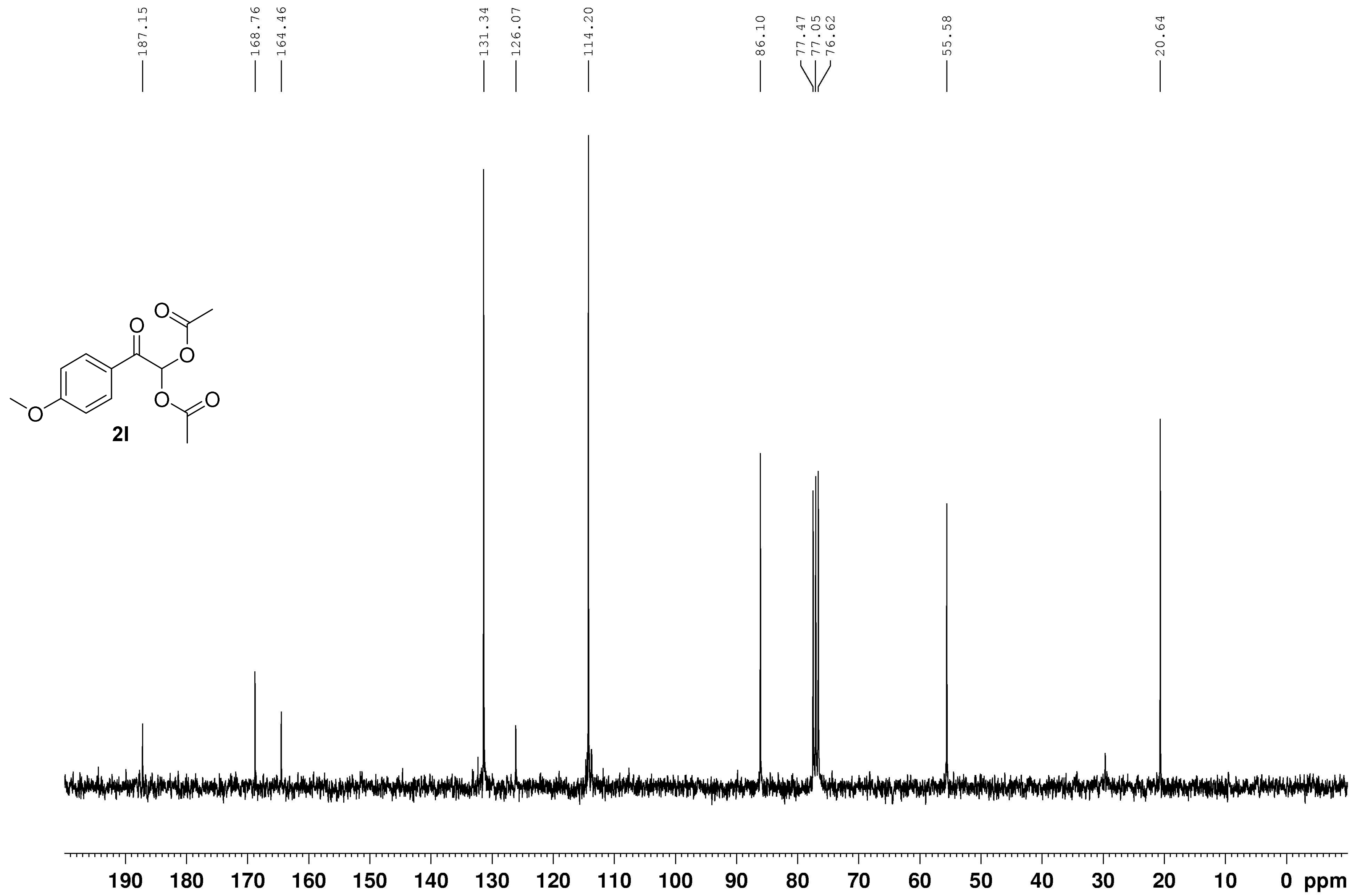

$^{13}C\{^{1}H\}$ NMR of compound **2l** (75 MHz, $CDCl_3$)

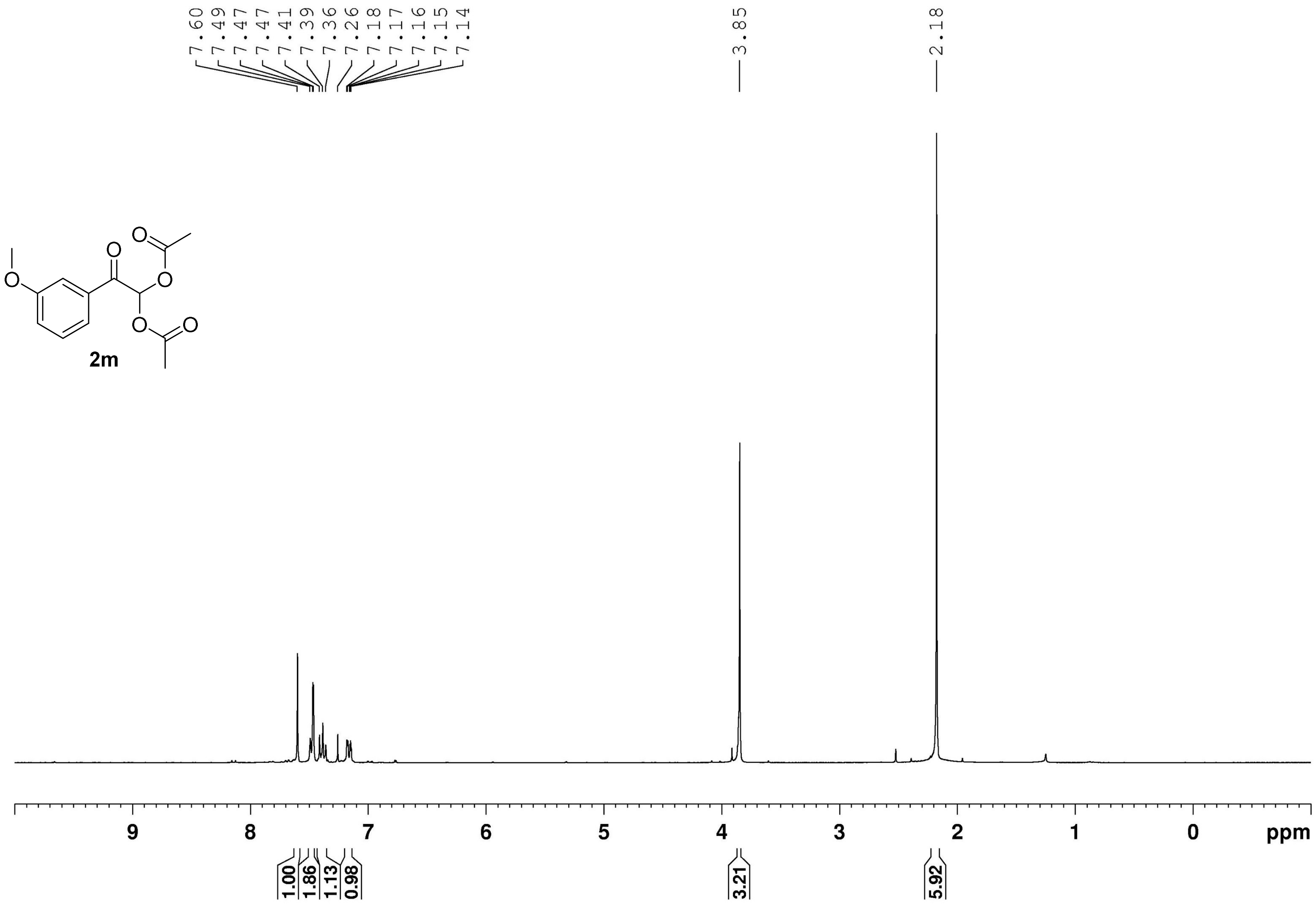

^{1}H NMR of compound **2m** (300 MHz, $CDCl_3$)

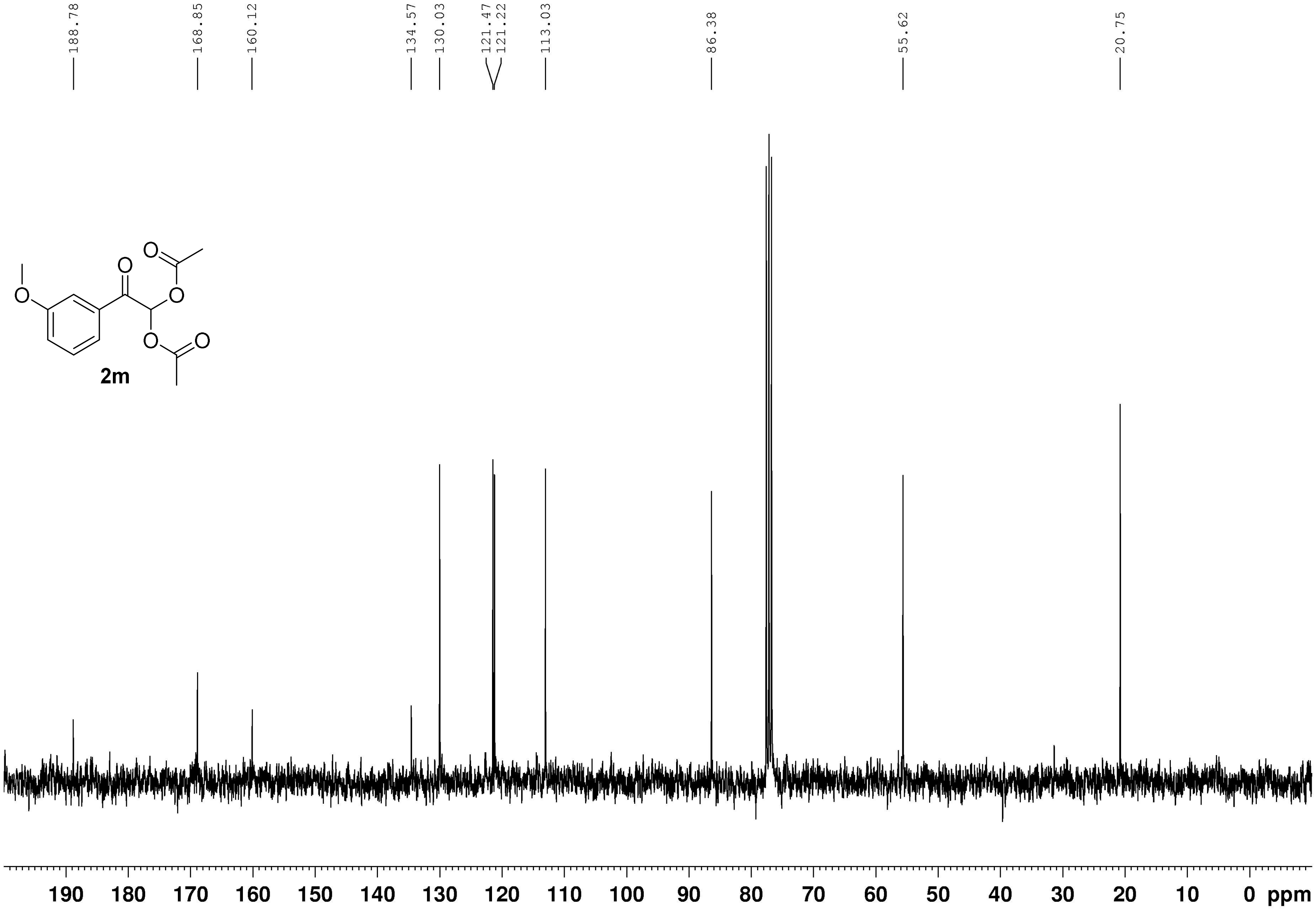

$^{13}C\{^{1}H\}$ NMR of compound **2m** (75 MHz, $CDCl_3$)

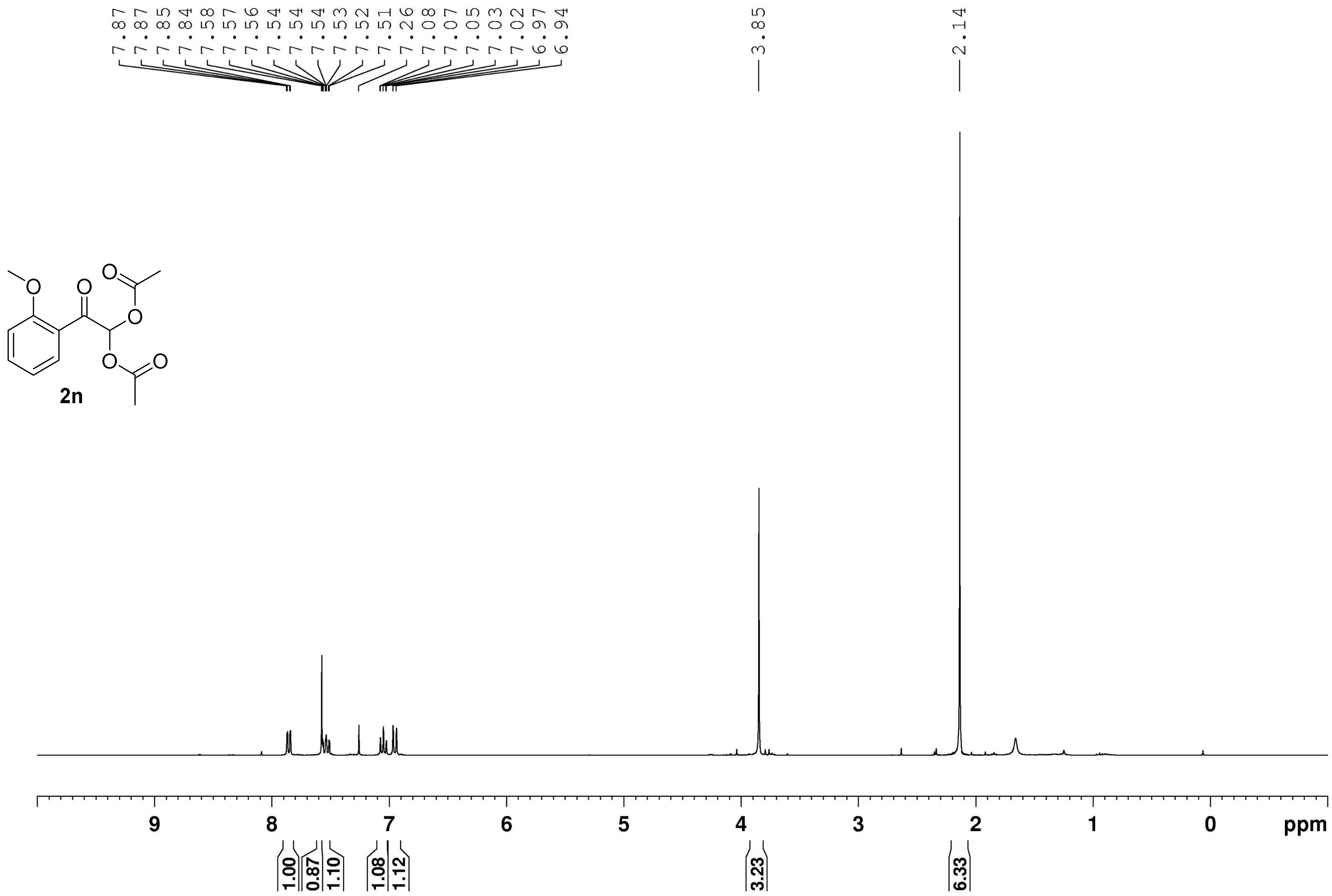

^{1}H NMR of compound **2n** (300 MHz, $CDCl_3$)

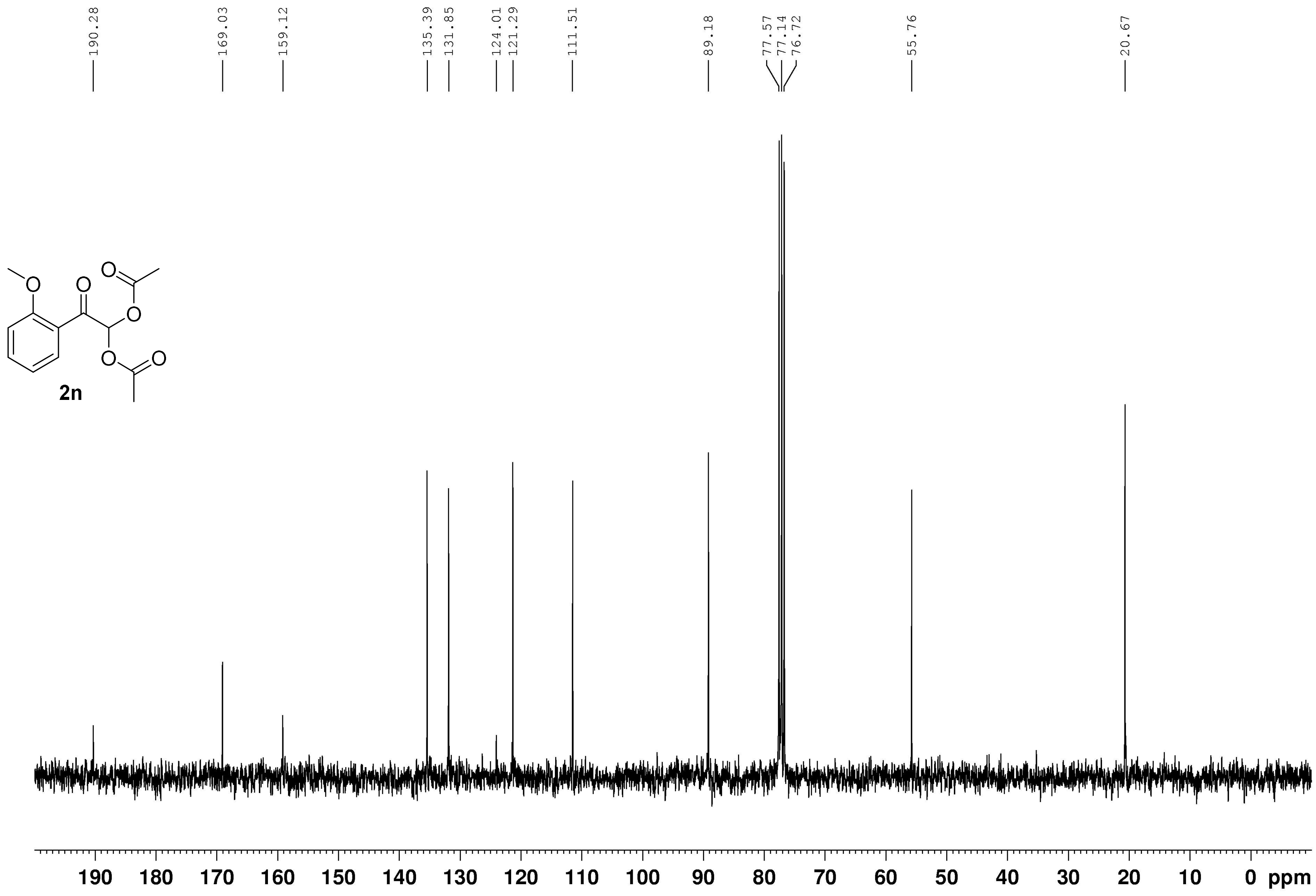

$^{13}C\{^1H\}$ NMR of compound **2n** (75 MHz, $CDCl_3$)

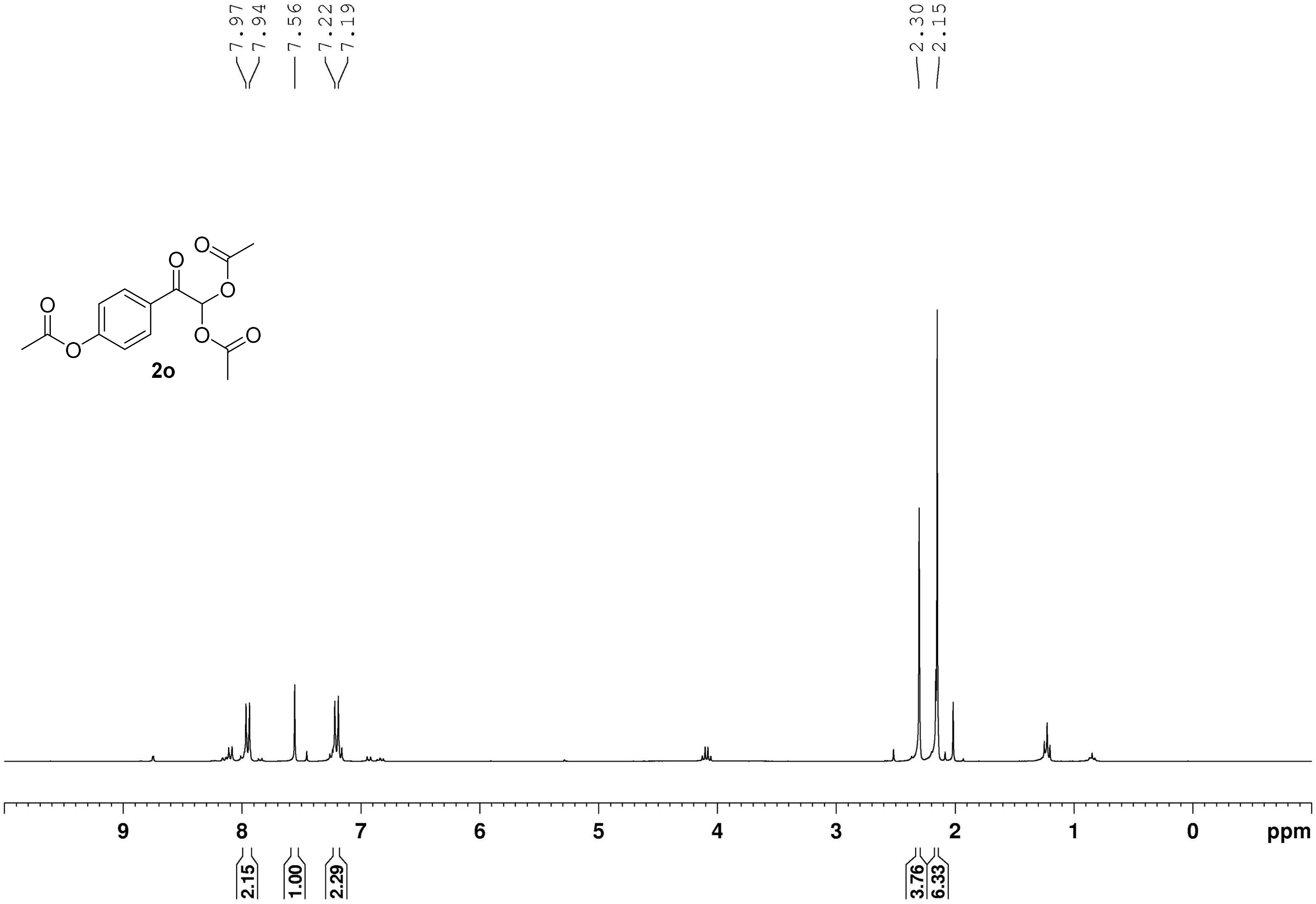

^{1}H NMR of compound **2o** (300 MHz, $CDCl_3$)

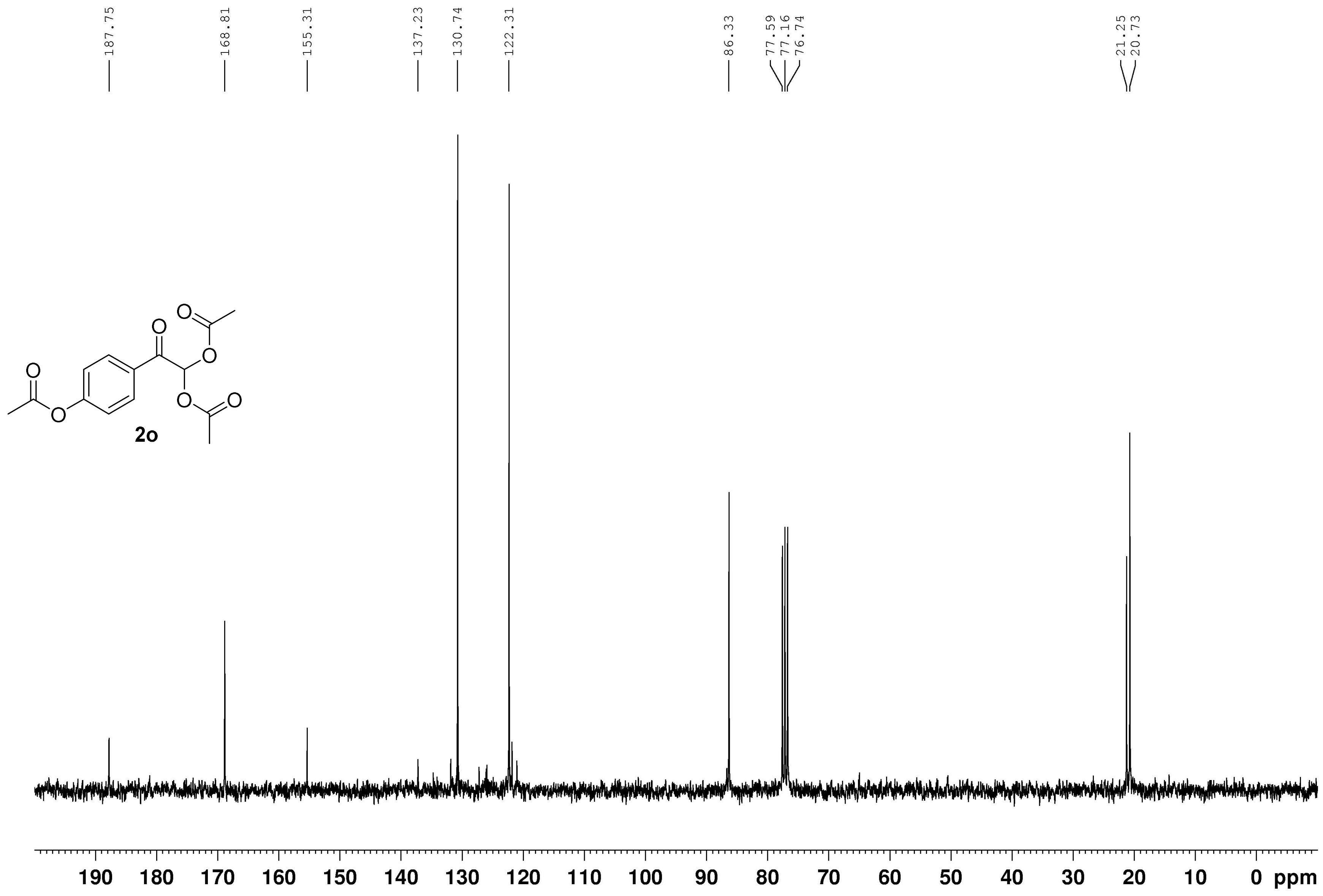

$^{13}C\{^{1}H\}$ NMR of compound **2o** (75 MHz, $CDCl_3$)

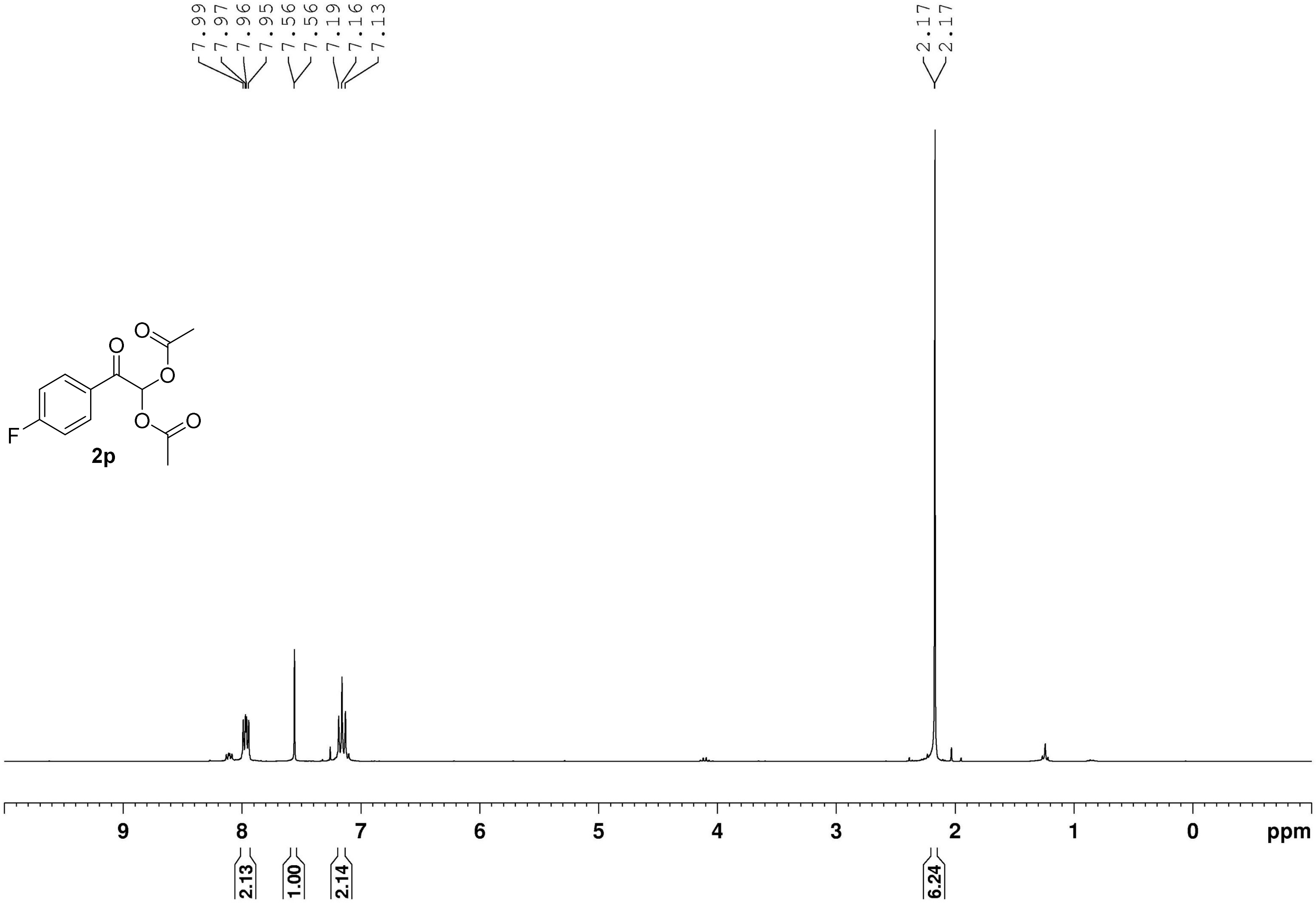

^{1}H NMR of compound **2p** (300 MHz, $CDCl_3$)

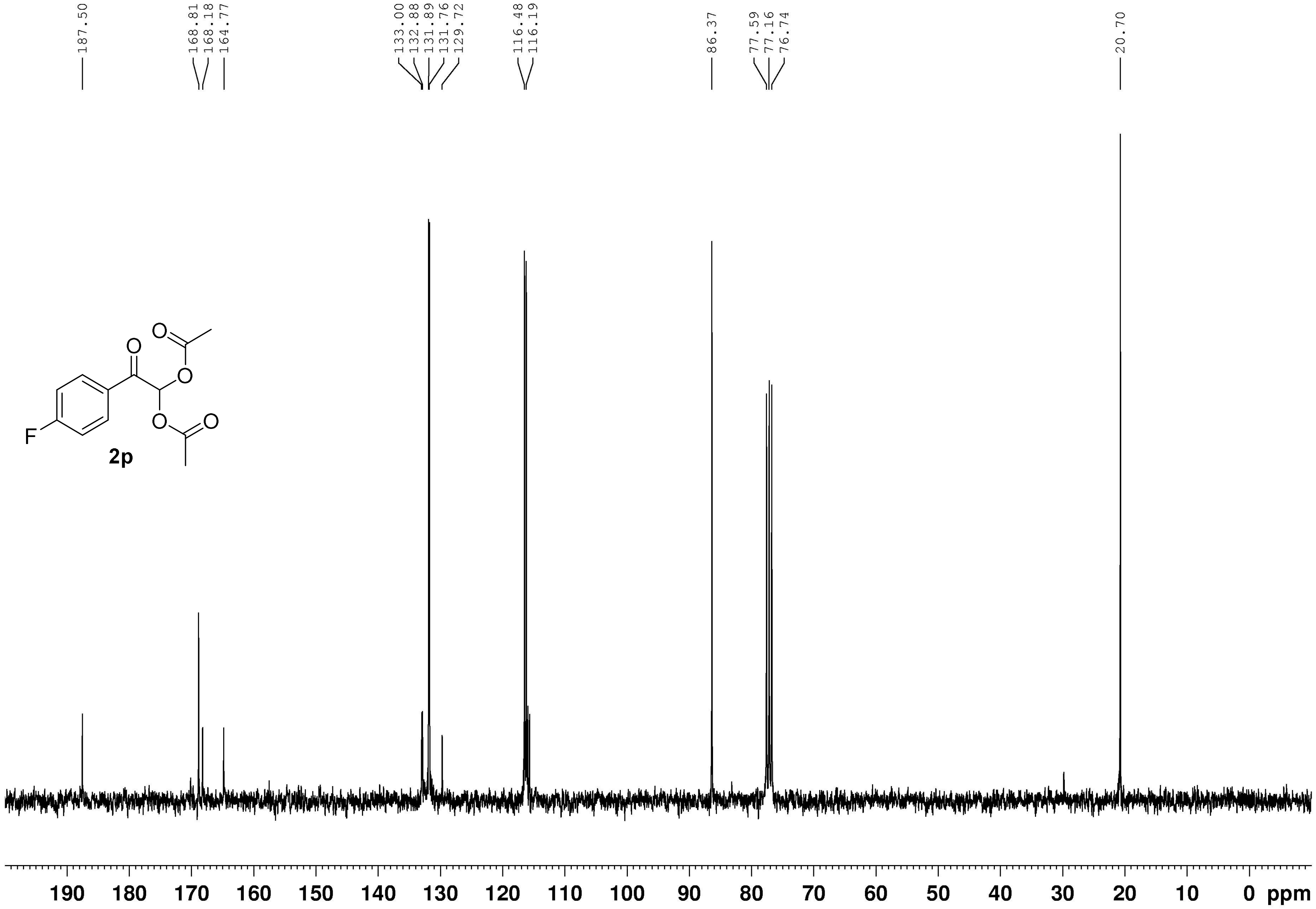

$^{13}C\{^{1}H\}$ NMR of compound **2p** (75 MHz, $CDCl_3$)

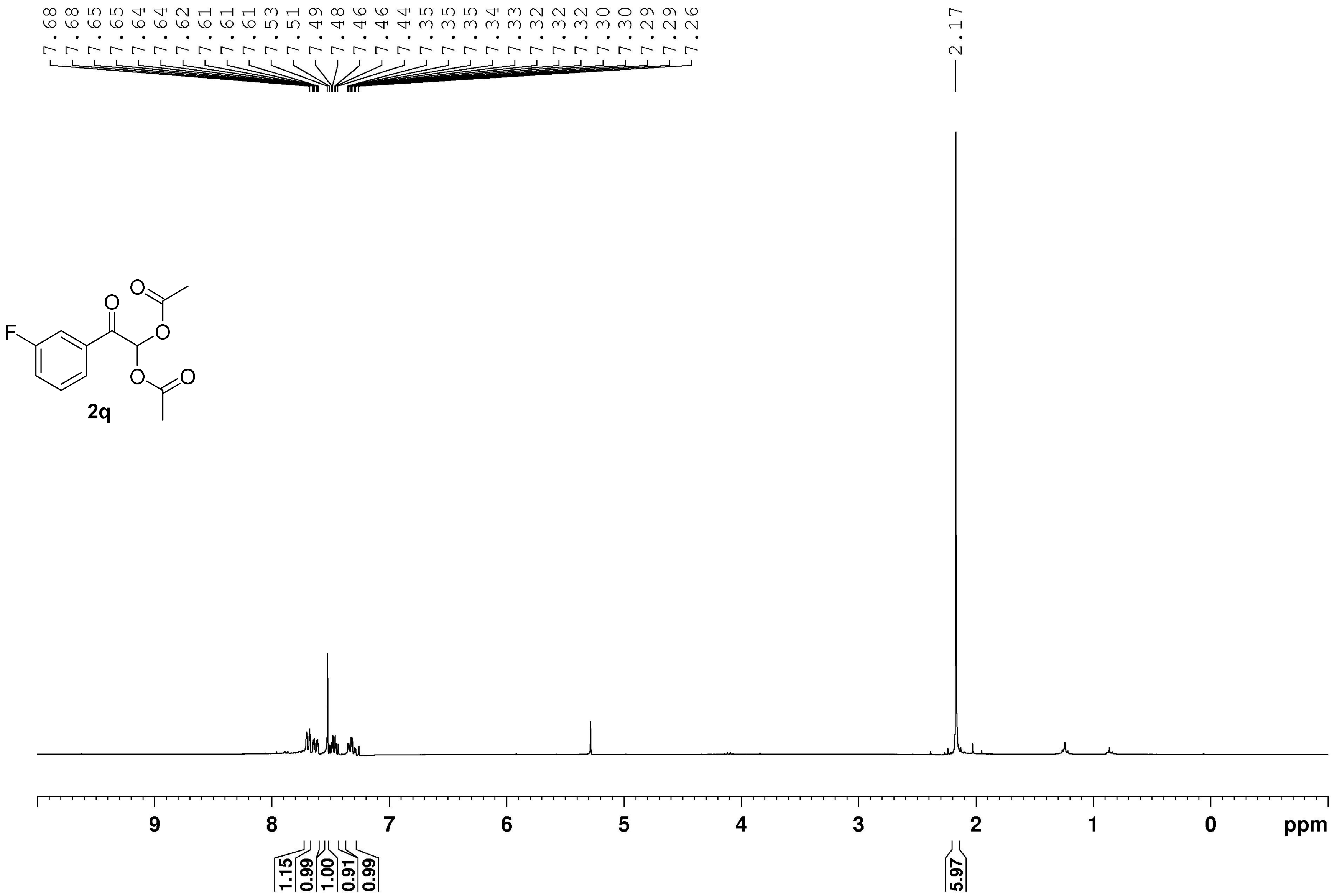

^{1}H NMR of compound **2q** (300 MHz, $CDCl_3$)

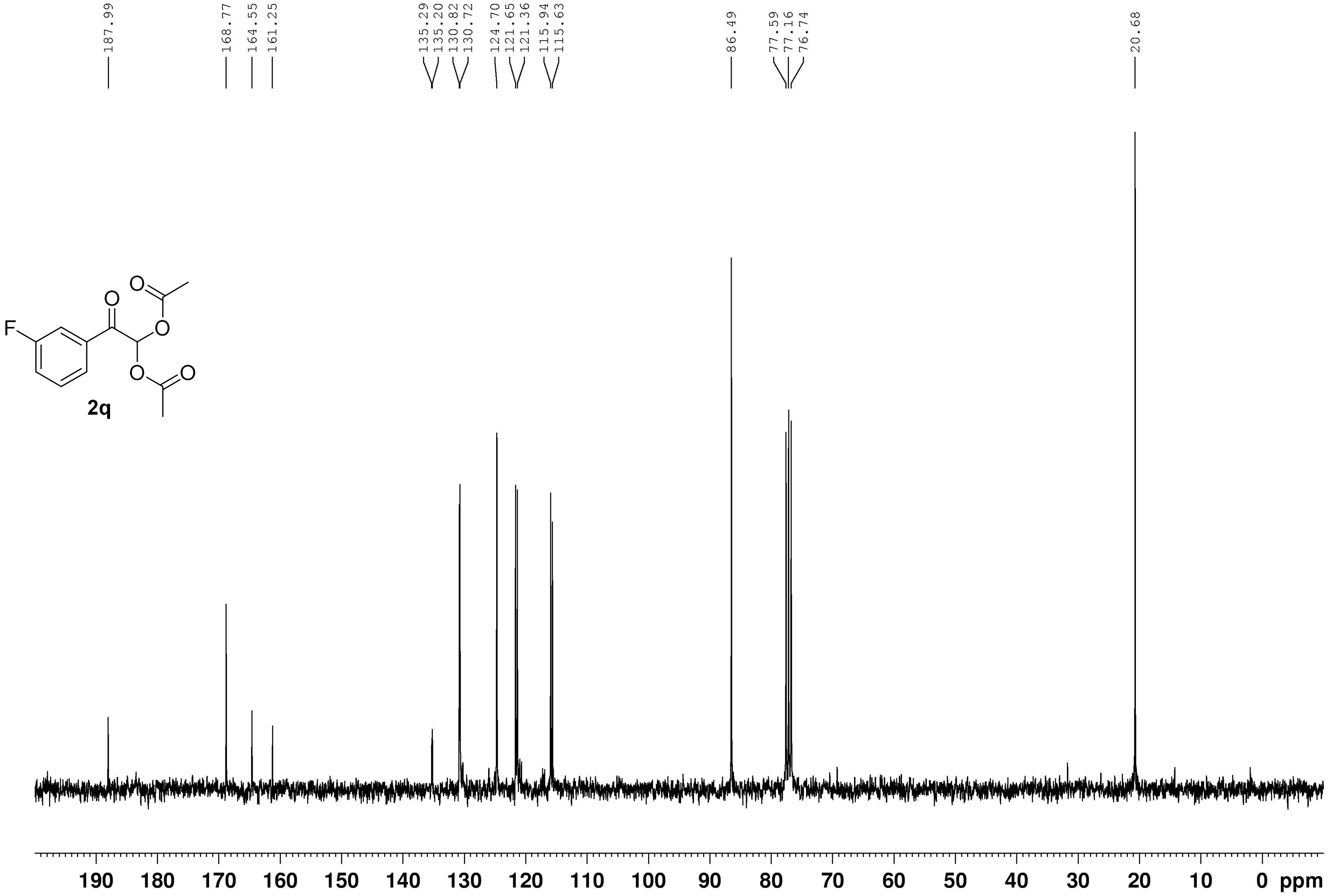

$^{13}C\{^{1}H\}$ NMR of compound **2q** (75 MHz, $CDCl_3$)

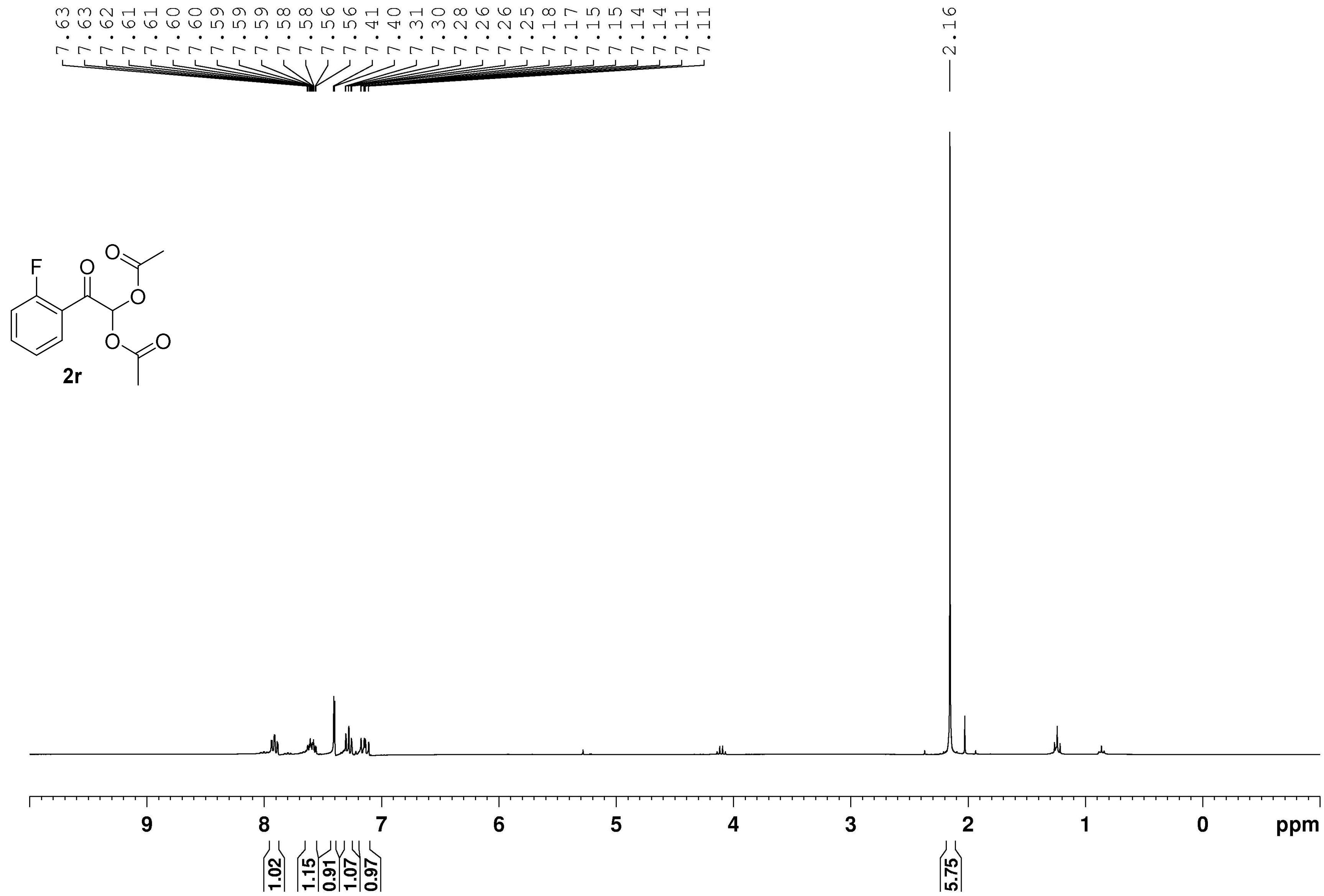

^{1}H NMR of compound **2r** (300 MHz, $CDCl_3$)

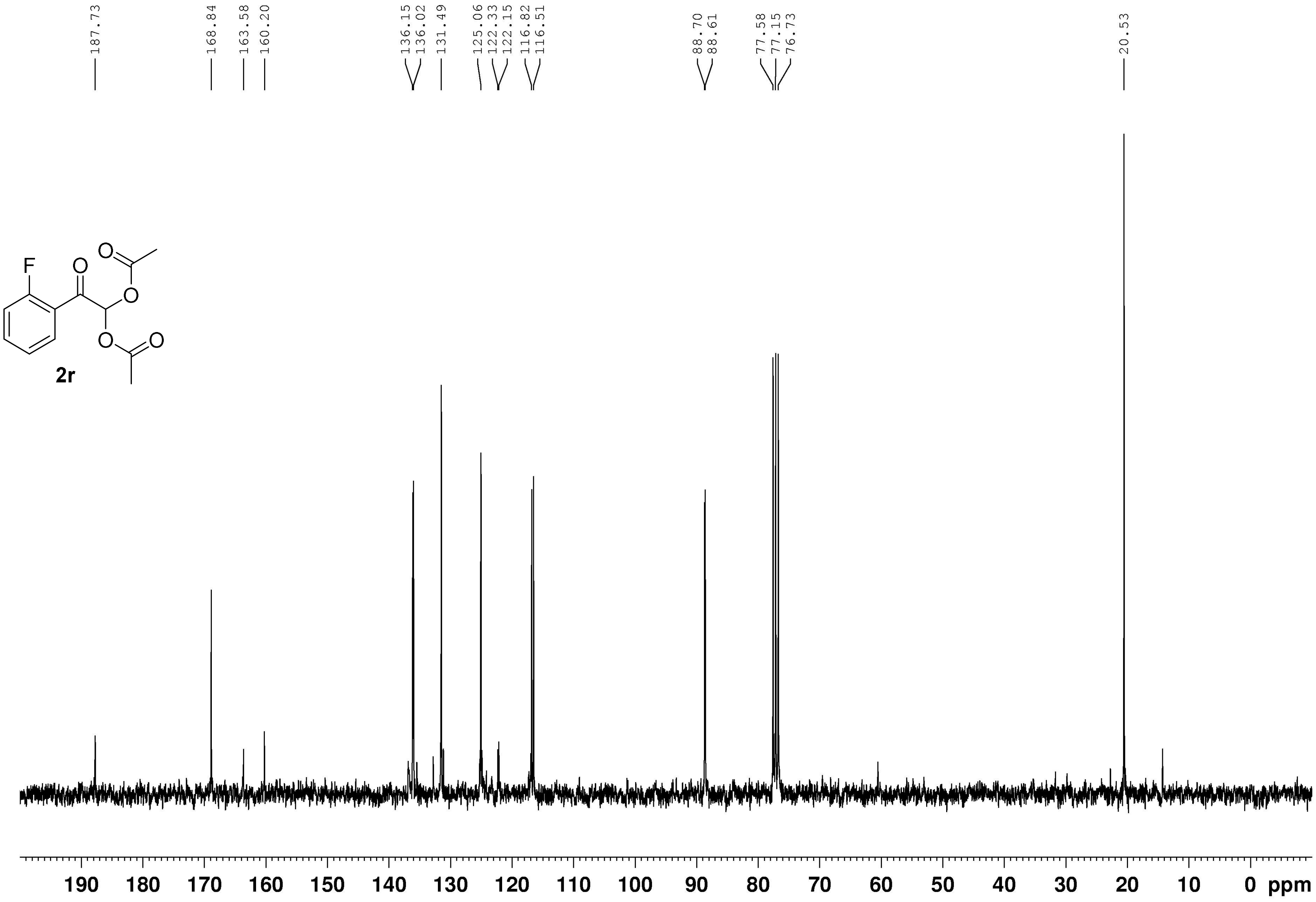

$^{13}C\{^{1}H\}$ NMR of compound **2r** (75 MHz, $CDCl_3$)

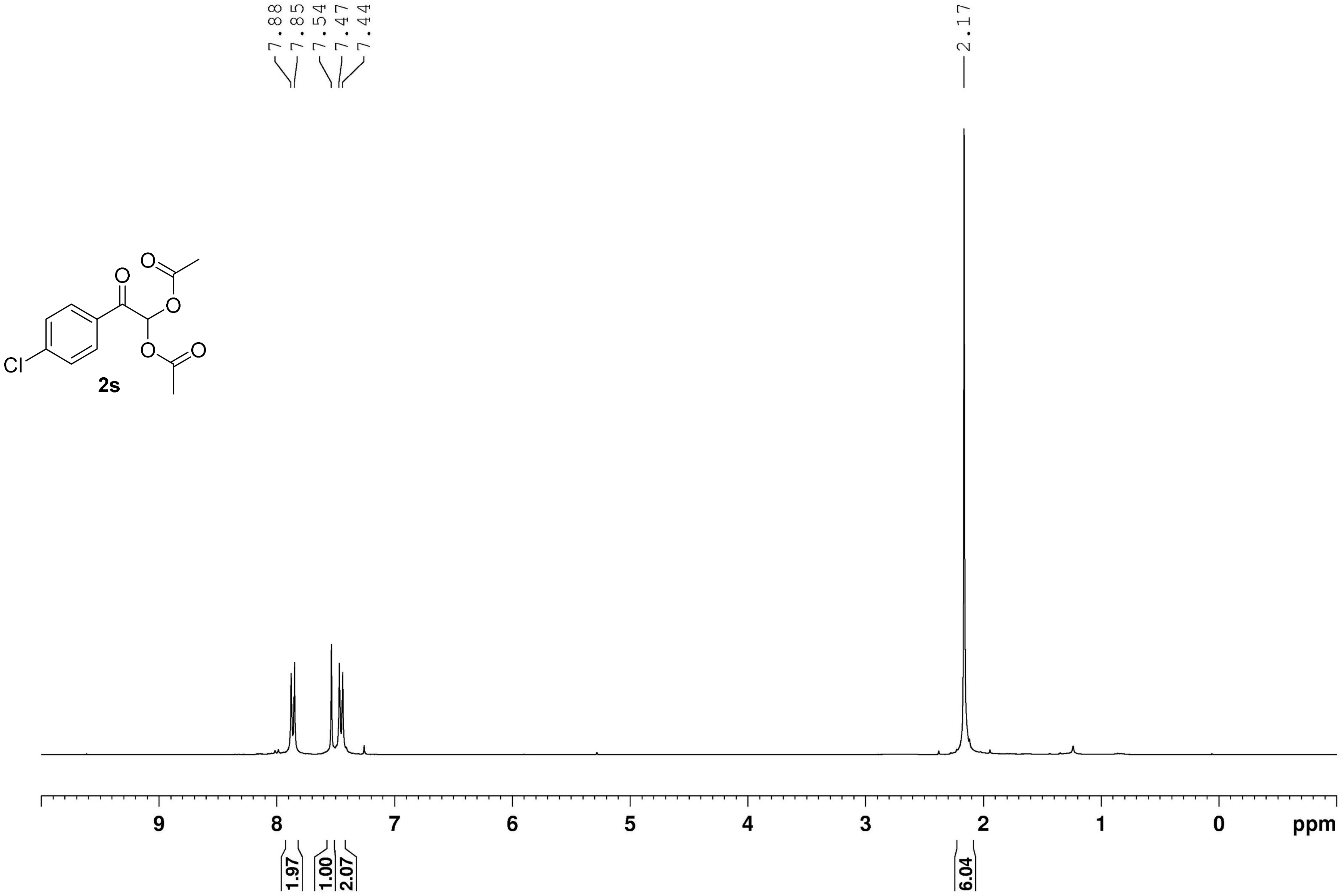

^{1}H NMR of compound **2s** (300 MHz, $CDCl_3$)

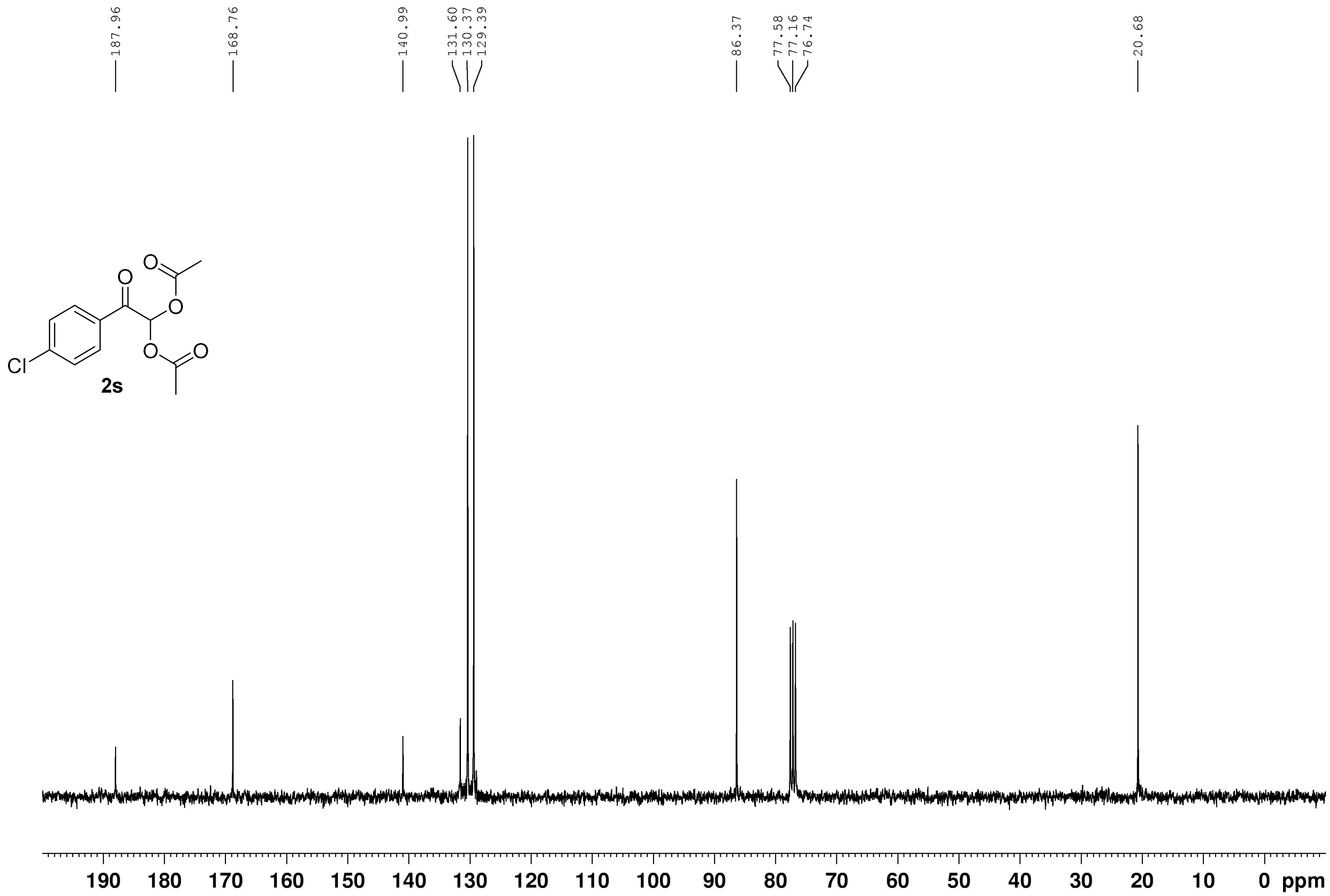

$^{13}C\{^{1}H\}$ NMR of compound **2s** (75 MHz, $CDCl_3$)

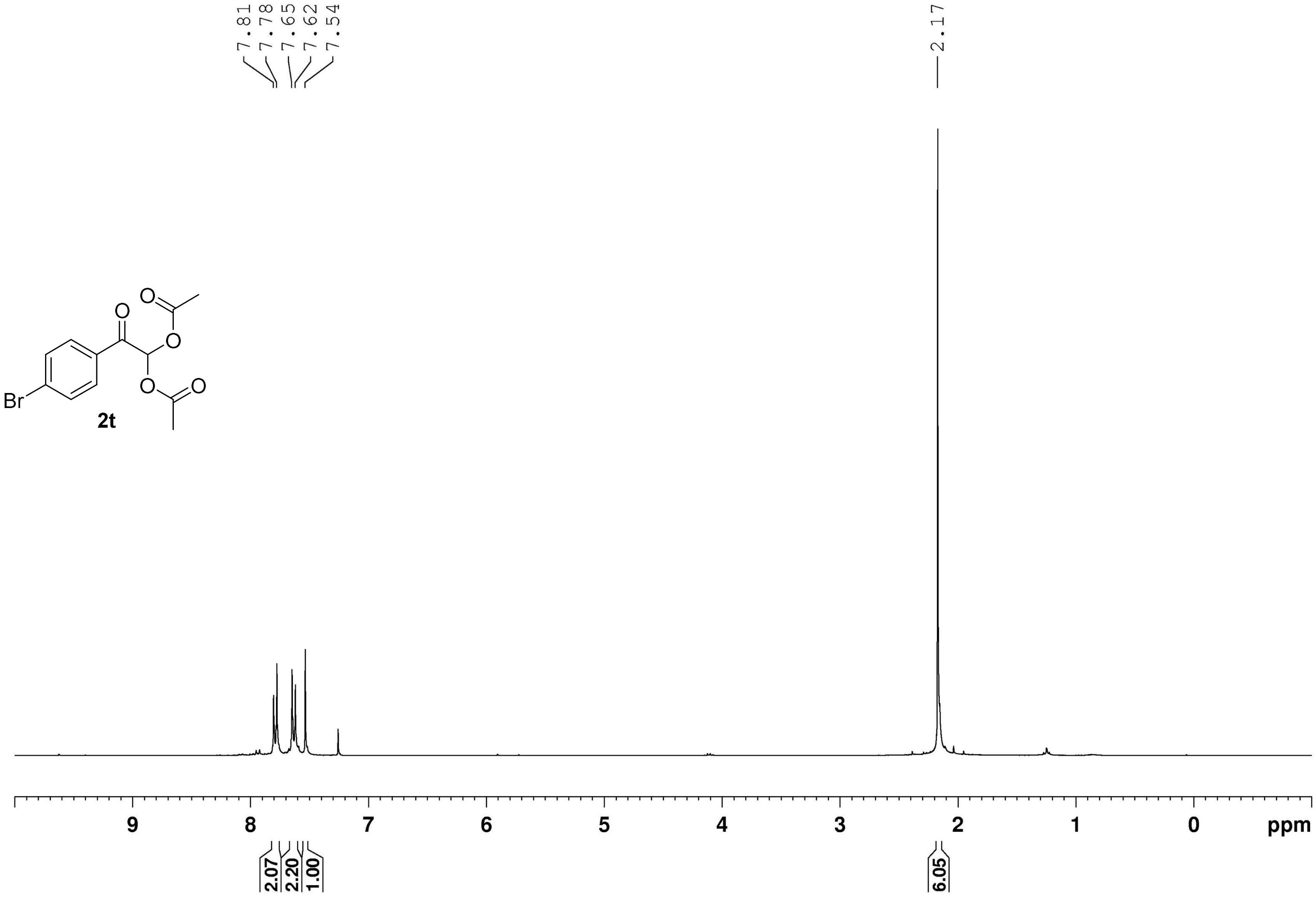

^{1}H NMR of compound **2t** (300 MHz, $CDCl_3$)

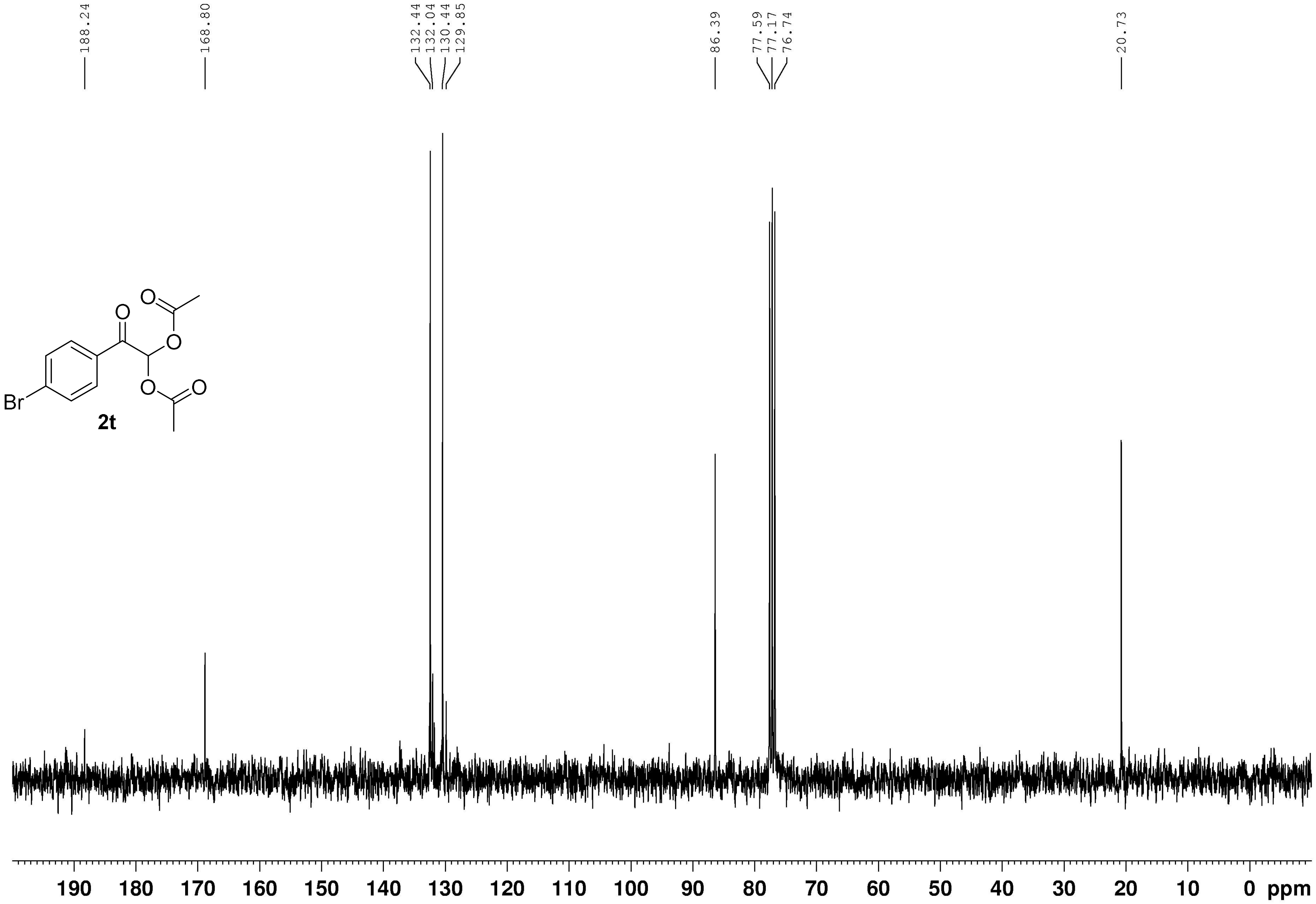

$^{13}C\{^{1}H\}$ NMR of compound **2t** (75 MHz, $CDCl_3$)

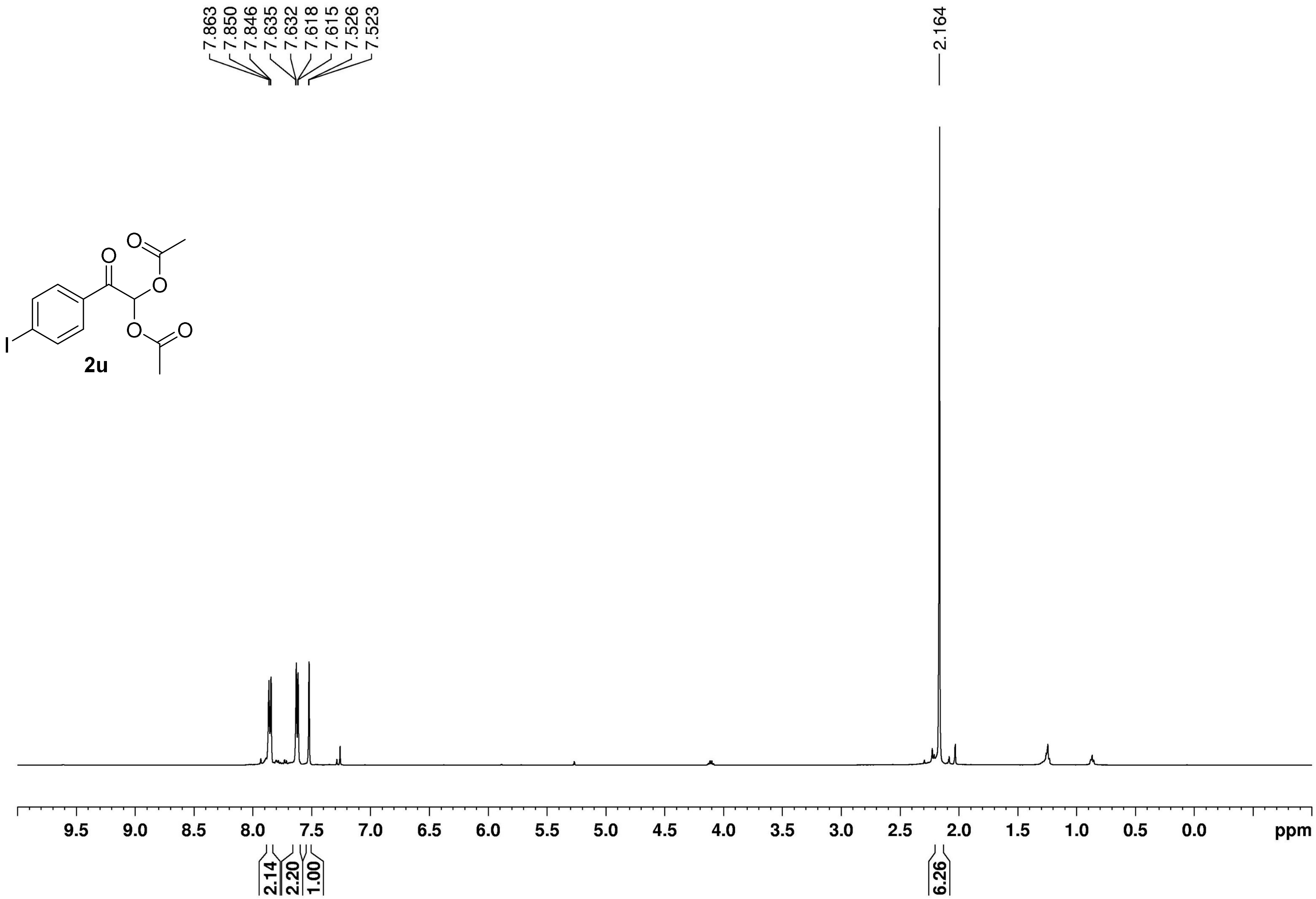

^{1}H NMR of compound **2u** (500 MHz, $CDCl_3$)

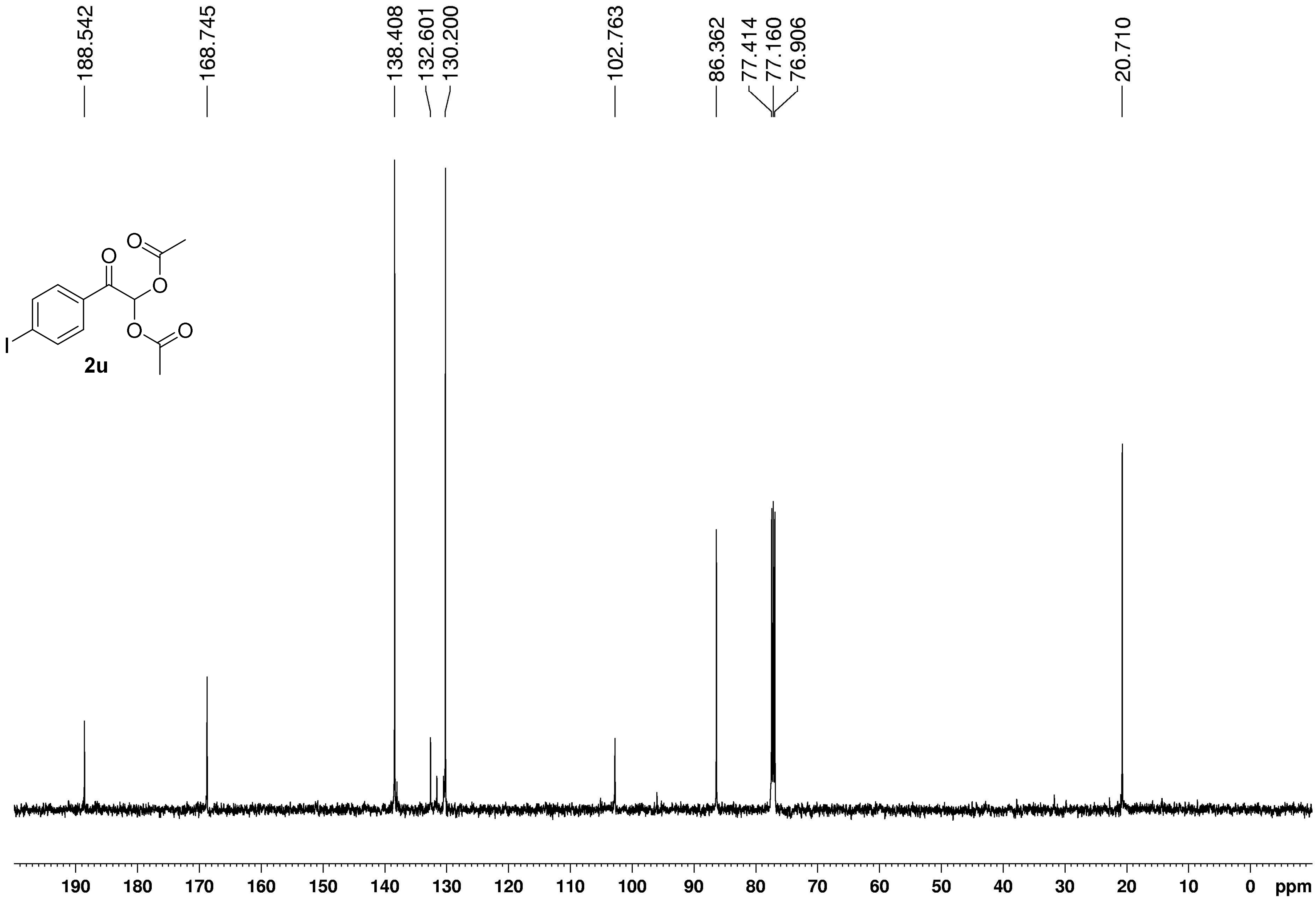

$^{13}C\{^1H\}$ NMR of compound **2u** (126 MHz, $CDCl_3$)

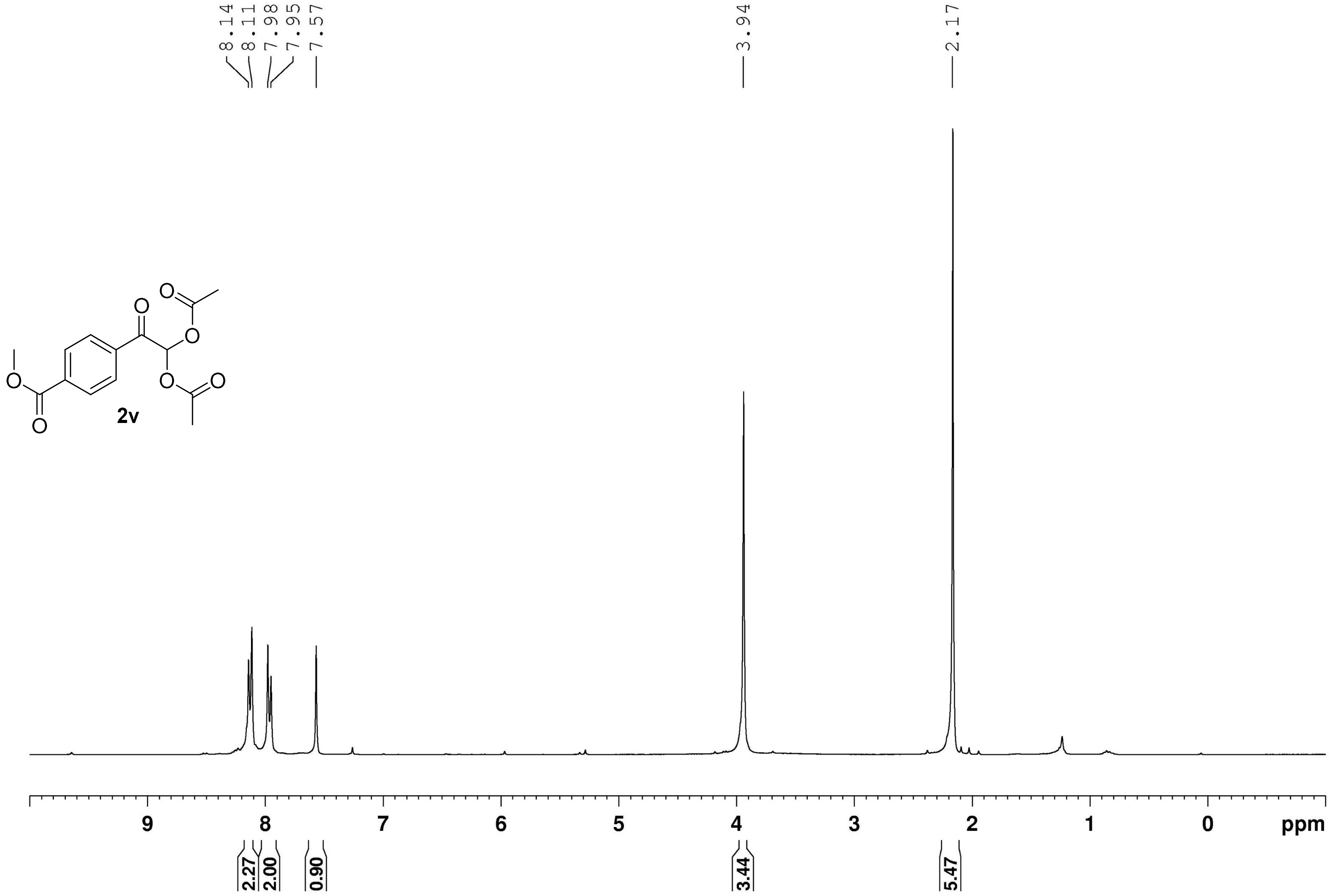

^{1}H NMR of compound **2v** (300 MHz, $CDCl_3$)

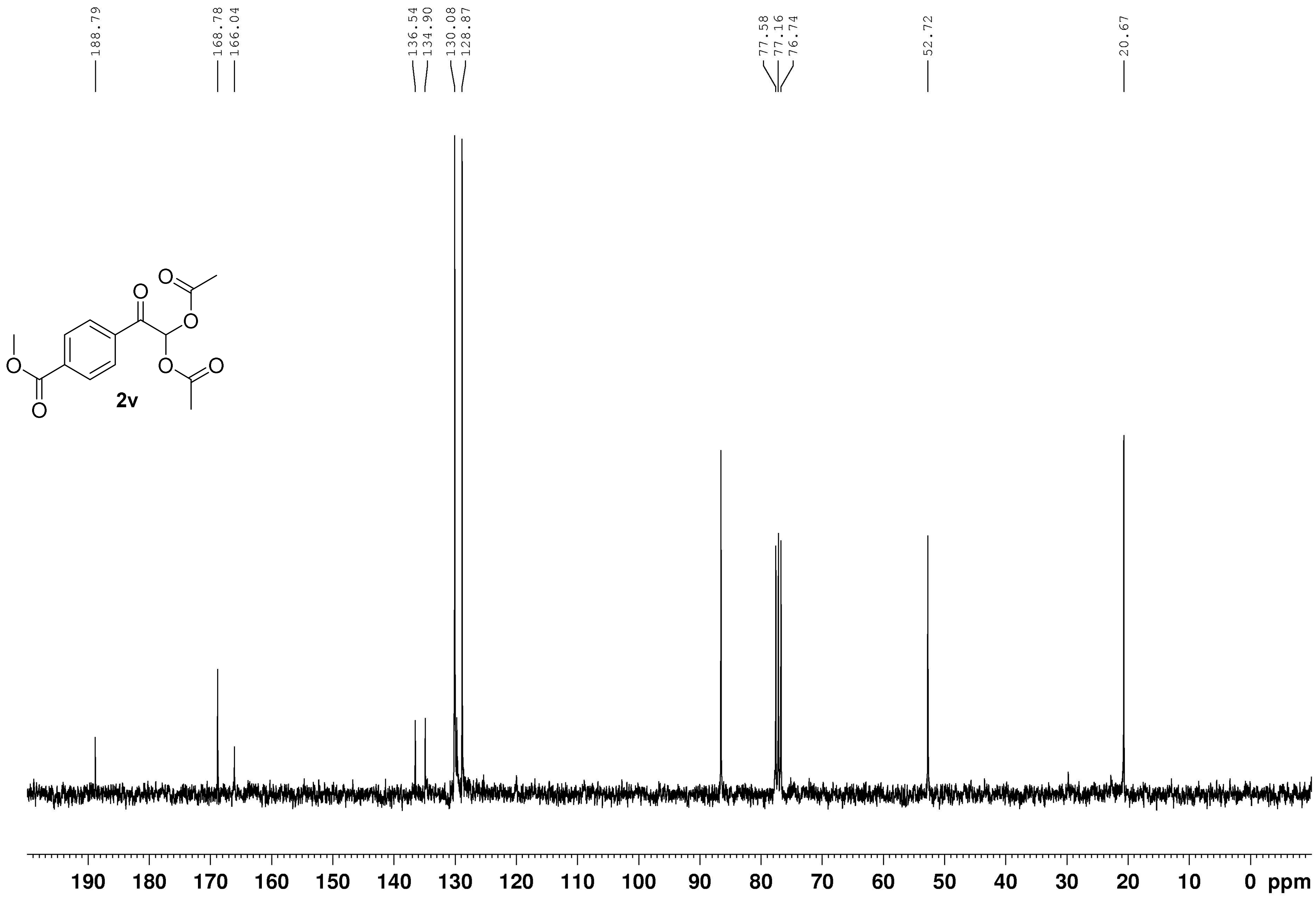

$^{13}C\{^{1}H\}$ NMR of compound **2v** (75 MHz, $CDCl_3$)

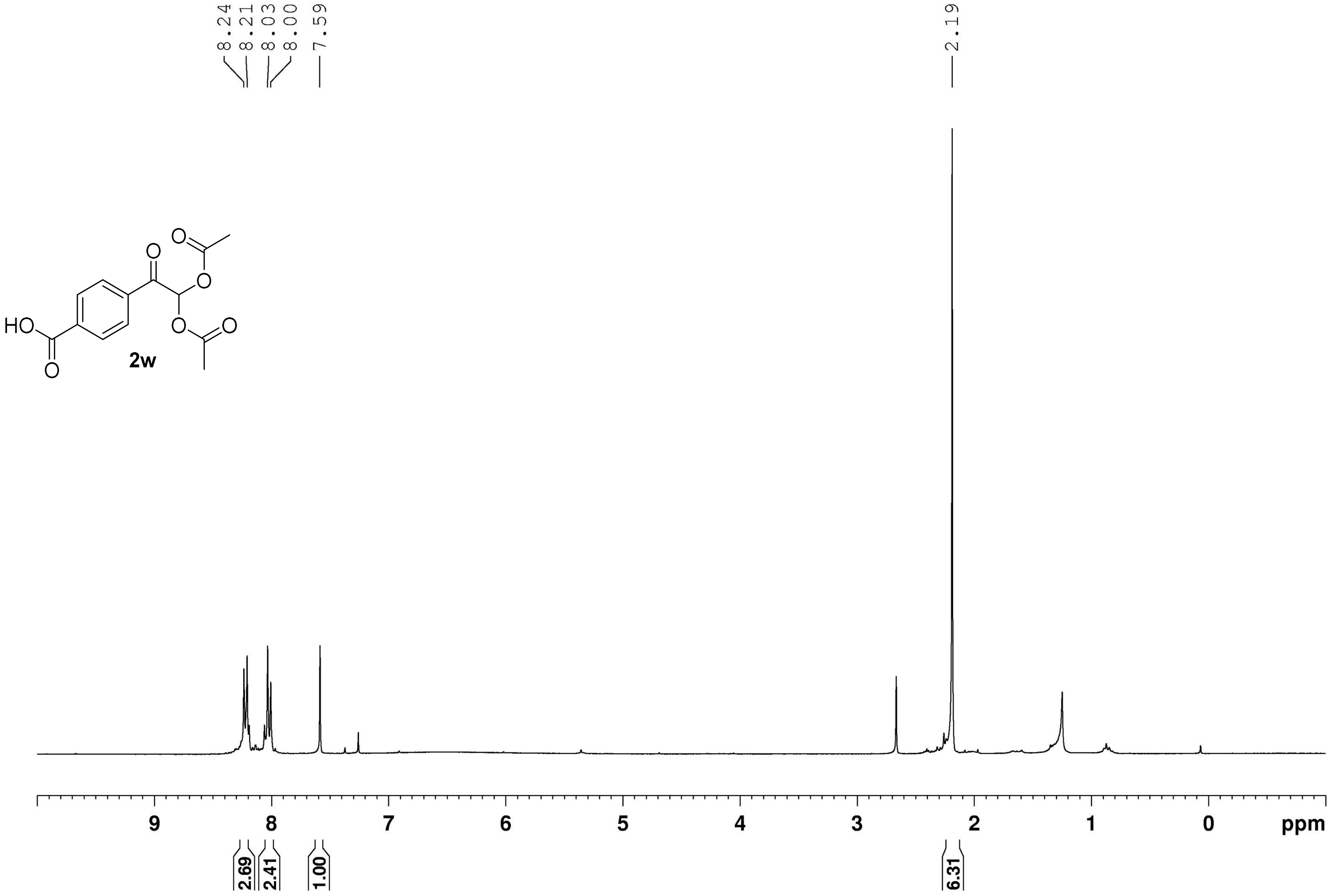

^{1}H NMR of compound **2w** (300 MHz, $CDCl_3$)

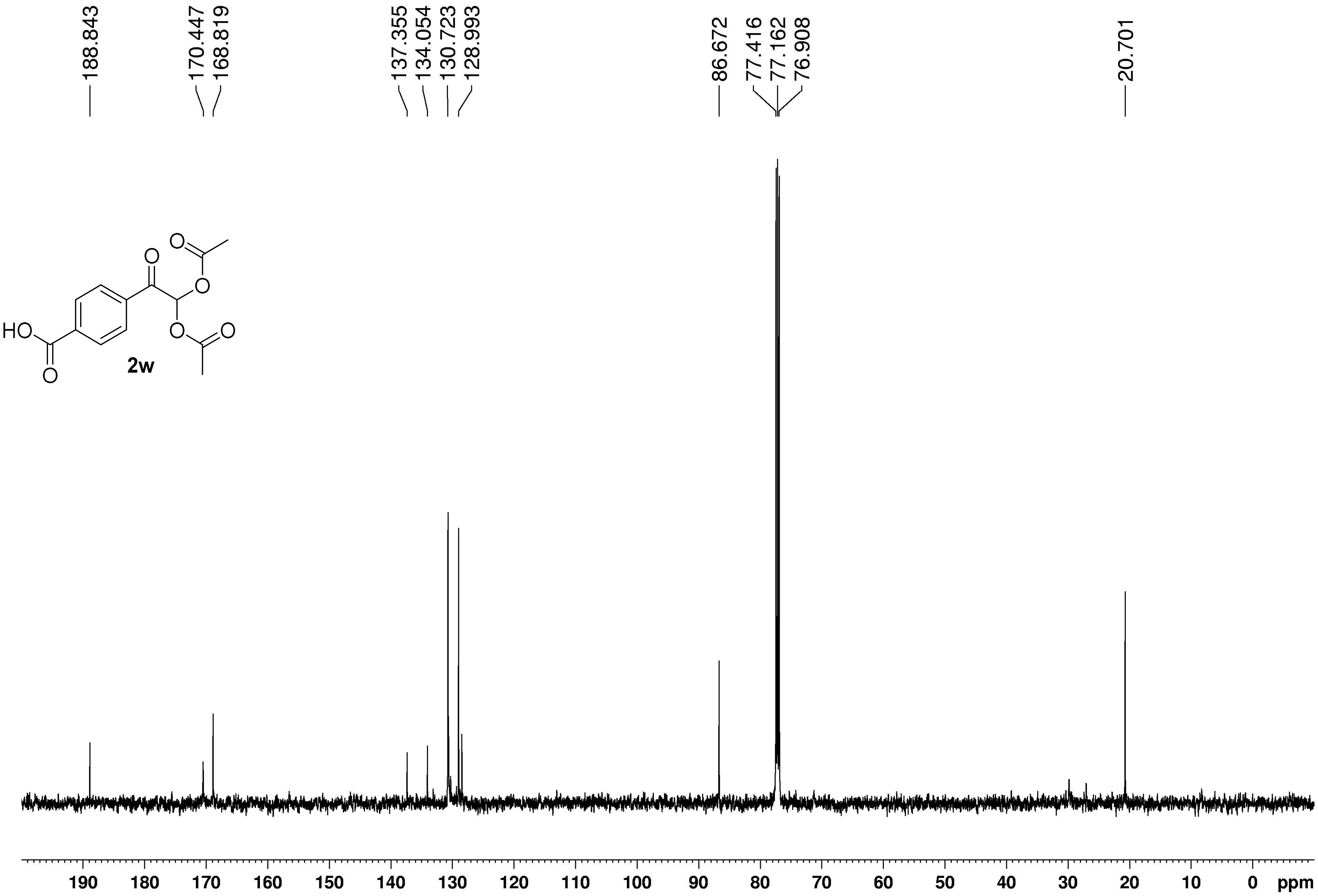

$^{13}C\{^{1}H\}$ NMR of compound **2w** (126 MHz, $CDCl_3$)

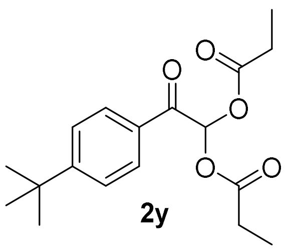
2y

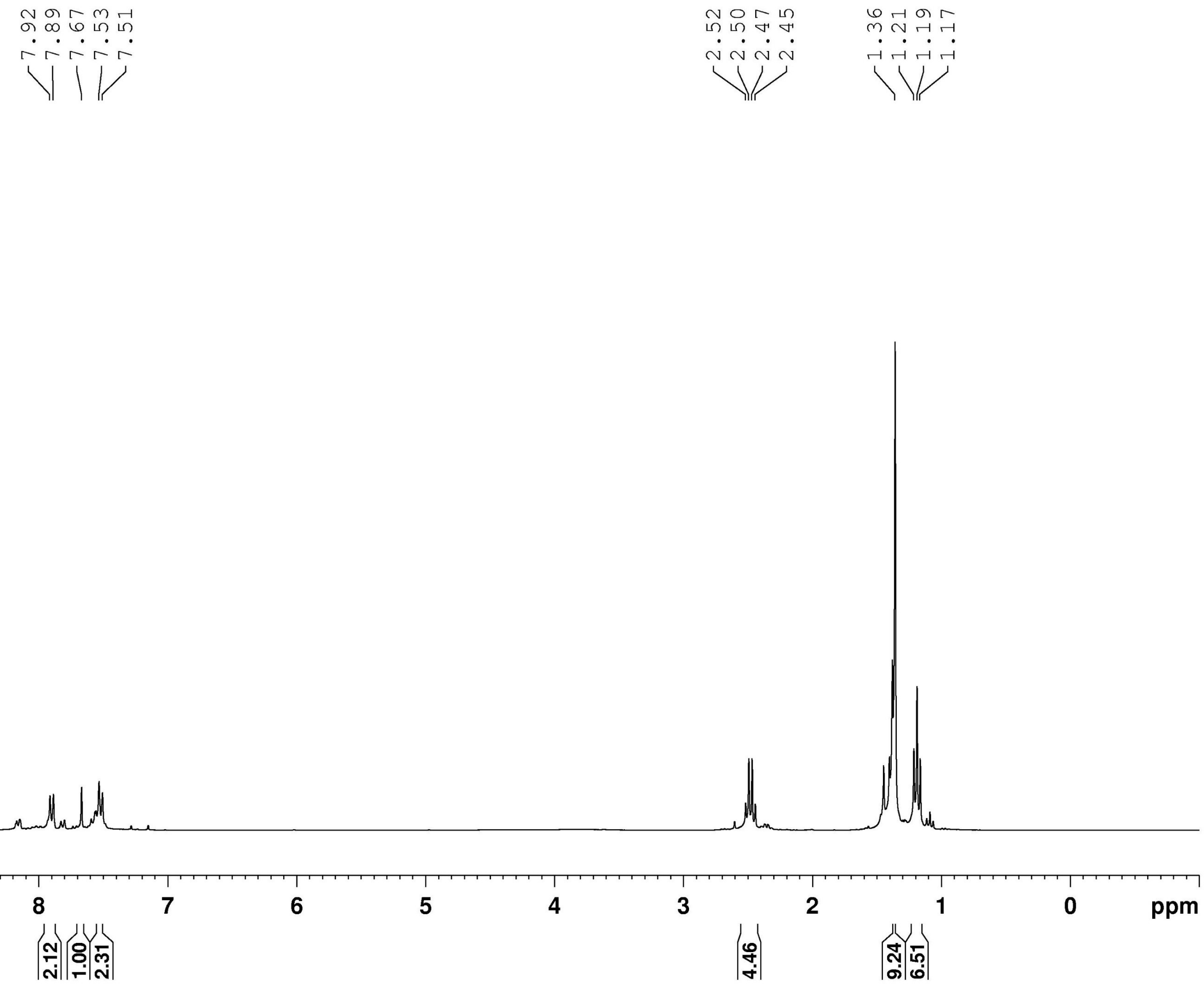

^{1}H NMR of compound **2y** (300 MHz, $CDCl_3$)

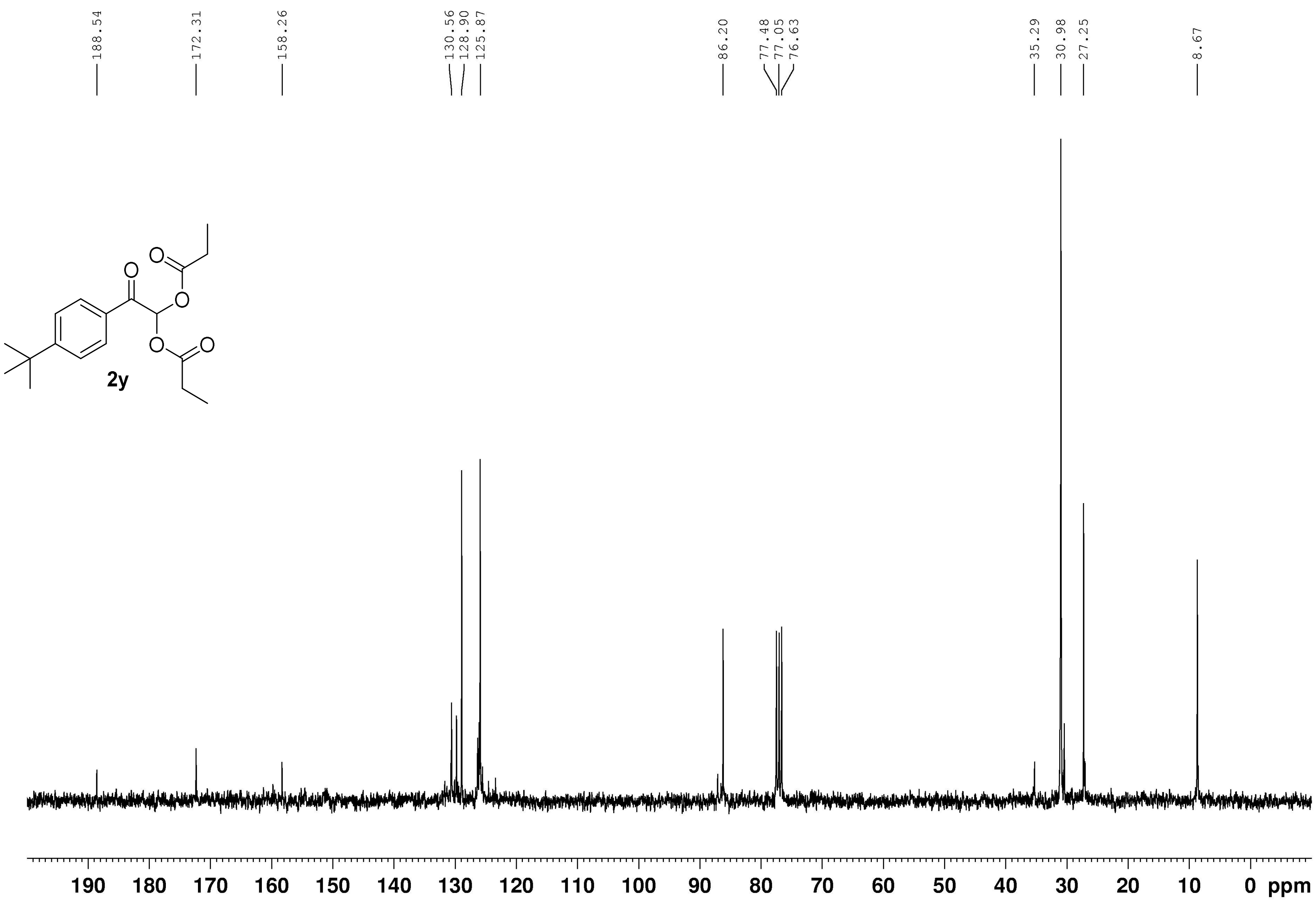

$^{13}C\{^{1}H\}$ NMR of compound **2y** (75 MHz, $CDCl_3$)

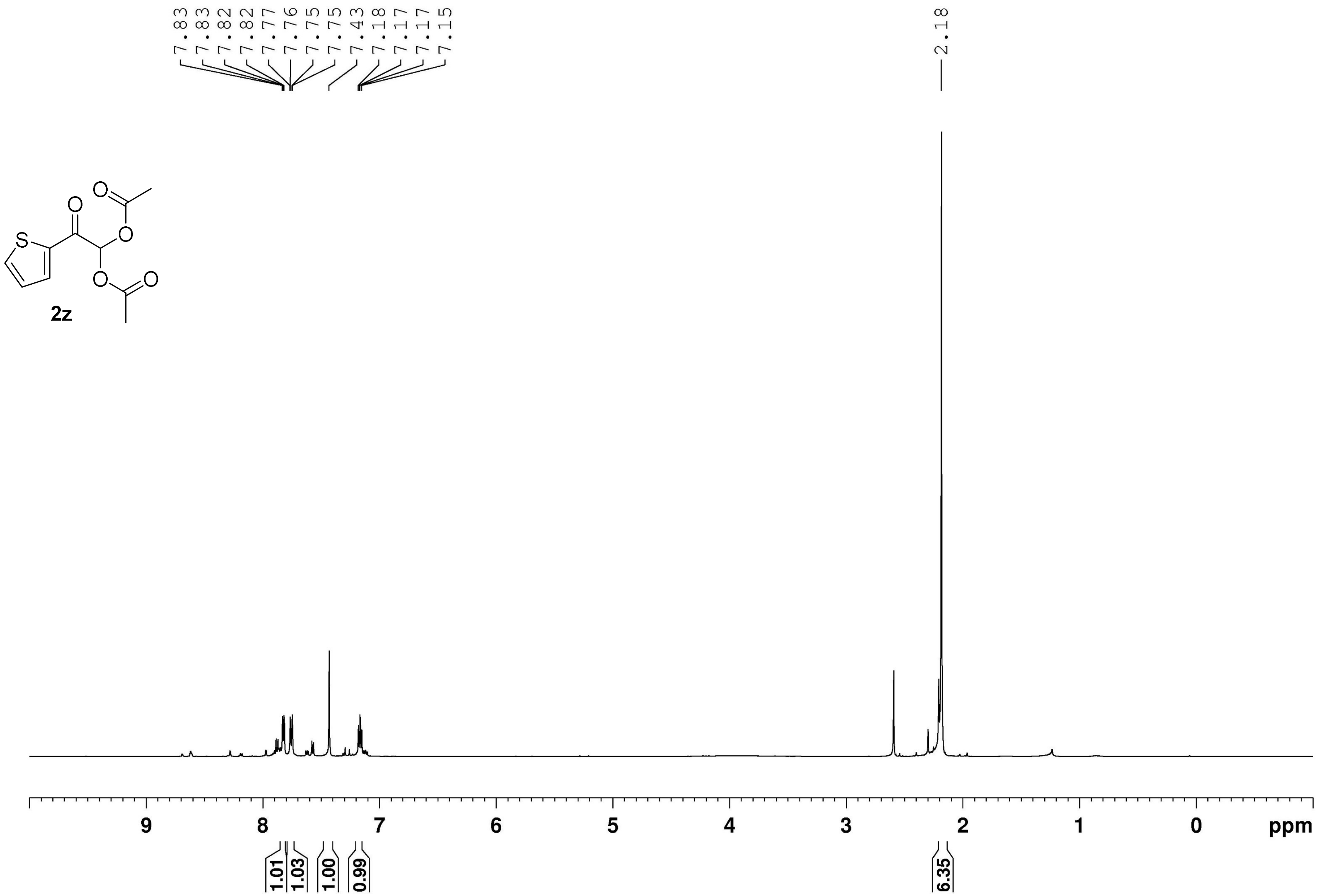

^{1}H NMR of compound **2z** (300 MHz, $CDCl_3$)

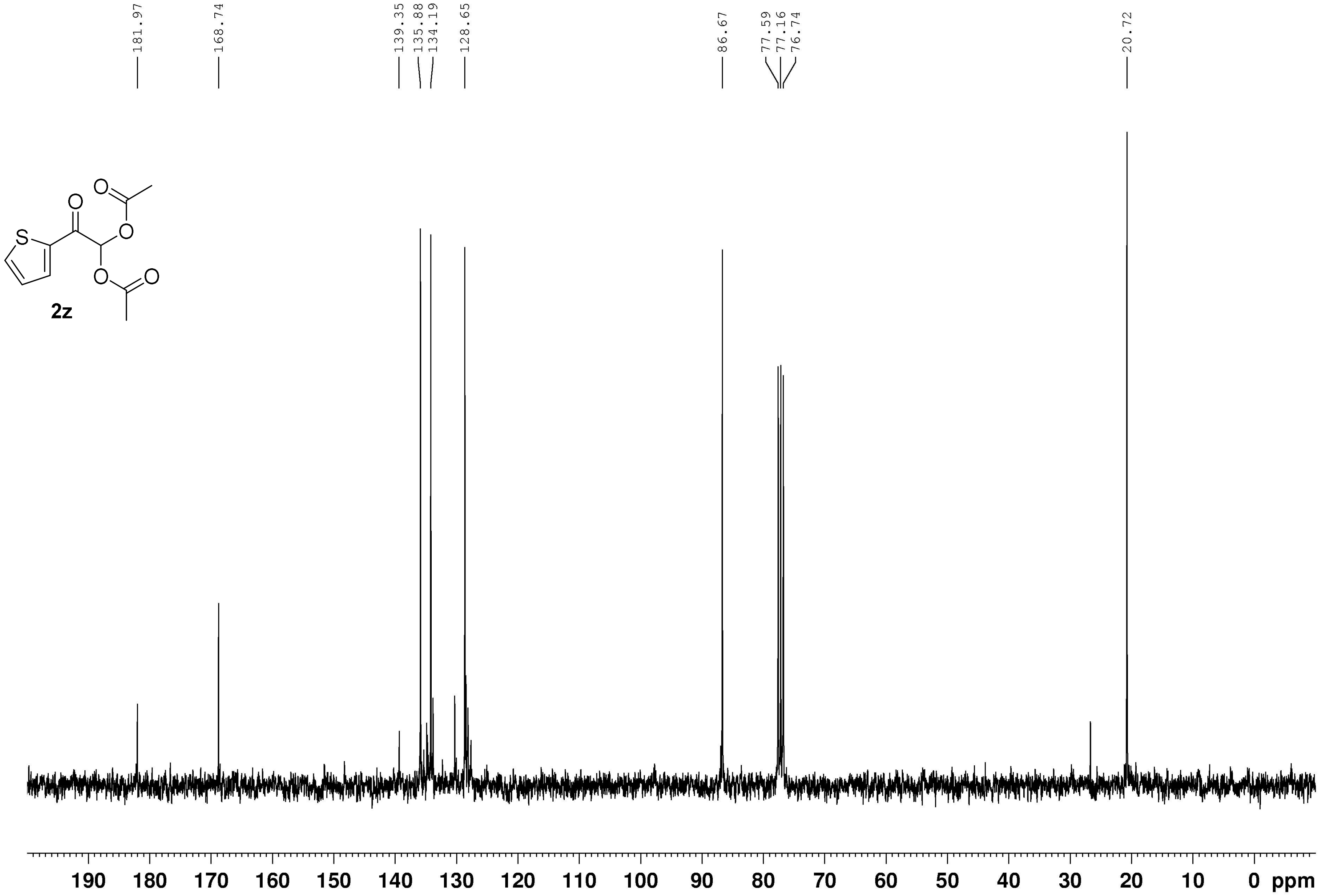

$^{13}C\{^{1}H\}$ NMR of compound **2z** (75 MHz, $CDCl_3$)

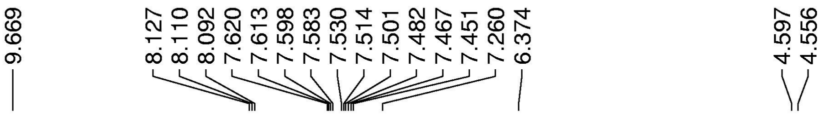

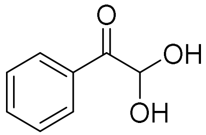

3

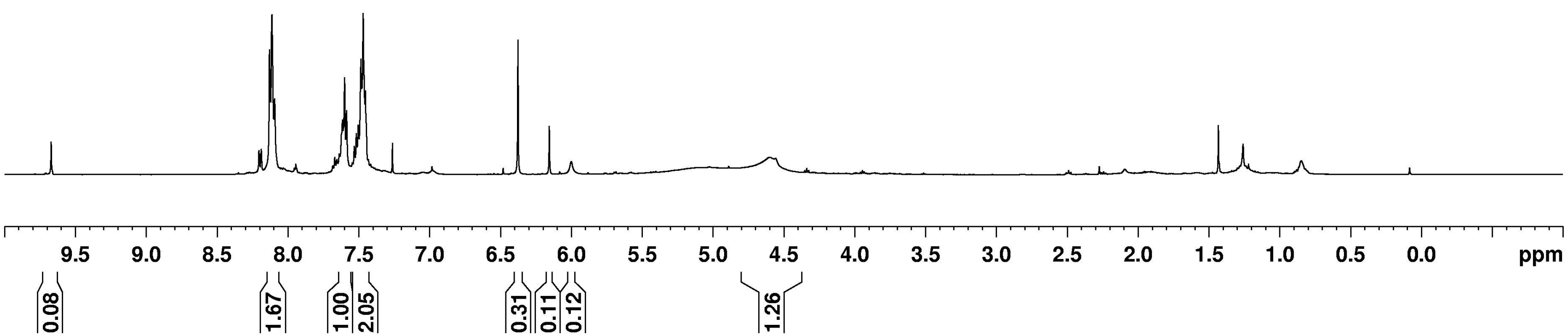

^{1}H NMR of compound **3** (300 MHz, $CDCl_3$)

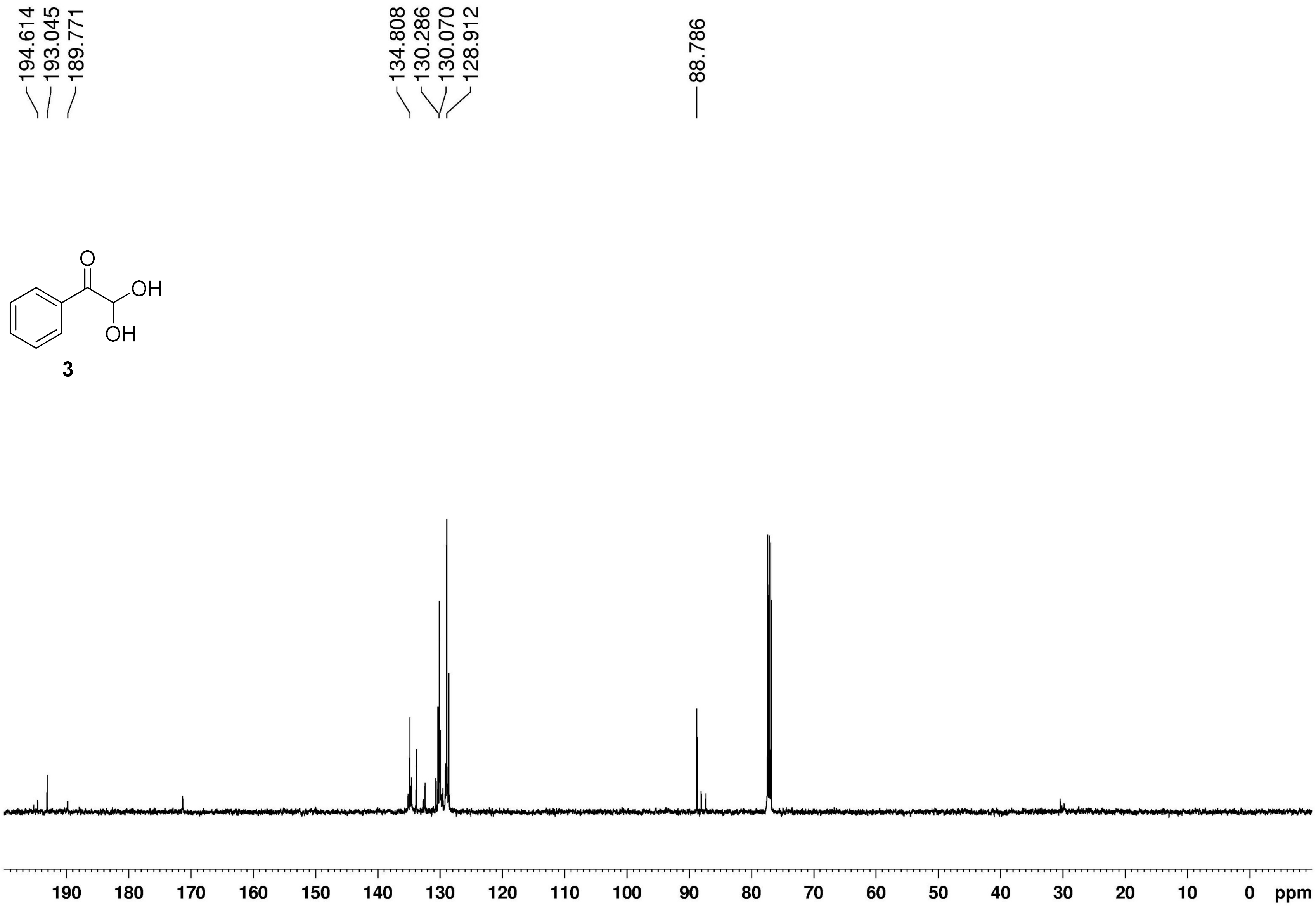

$^{13}C\{^{1}H\}$ of compound **3** NMR (75 MHz, $CDCl_3$)

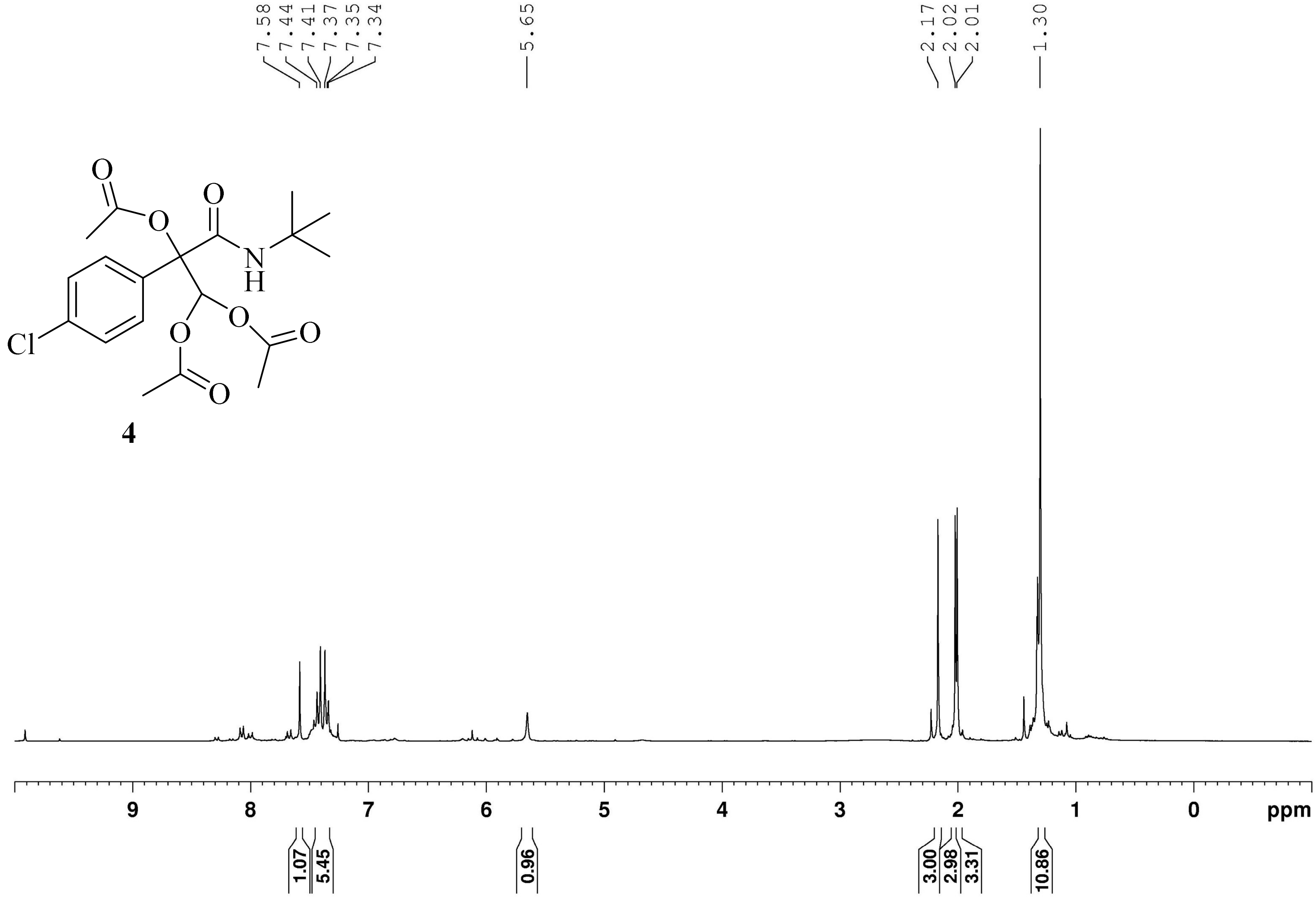

^{1}H NMR of compound **4** (300 MHz, $CDCl_3$)

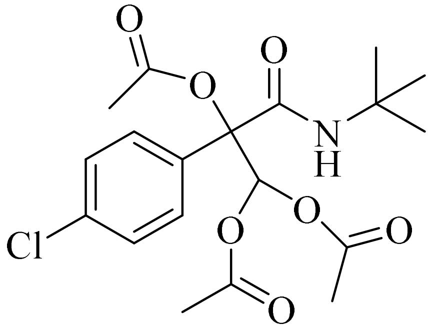

4

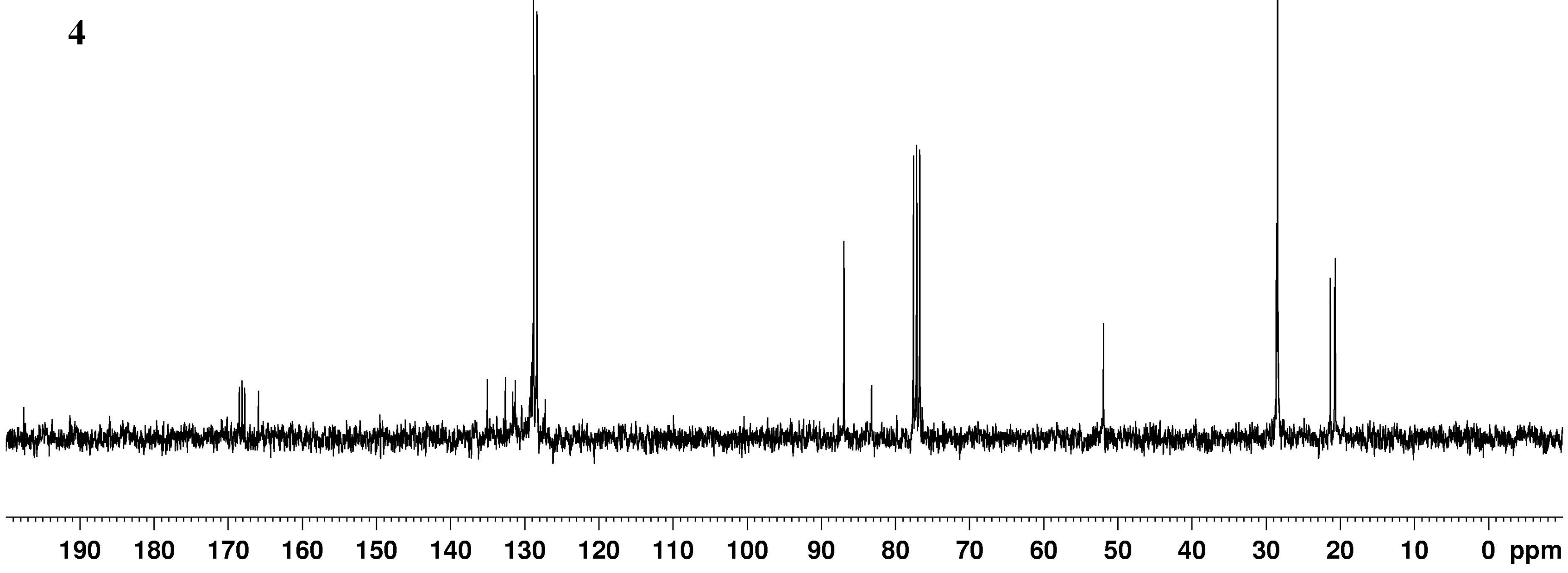

$^{13}C\{^{1}H\}$ NMR of compound **4** (75 MHz, $CDCl_3$)
